# Gene expression responses of threespine stickleback to salinity: implications for salt-sensitive hypertension

**DOI:** 10.3389/fgene.2014.00312

**Published:** 2014-09-11

**Authors:** Gang Wang, Ence Yang, Kerri J. Smith, Yong Zeng, Guoli Ji, Richard Connon, Nann A. Fangue, James J. Cai

**Affiliations:** ^1^Department of Veterinary Integrative Biosciences, Texas A&M UniversityCollege Station, TX, USA; ^2^Interdisciplinary Program in Genetics, Texas A&M UniversityCollege Station, TX, USA; ^3^Department of Automation, Xiamen UniversityXiamen, China; ^4^Department of Anatomy, Physiology and Cell Biology, School of Veterinary Medicine, University of CaliforniaDavis, CA, USA; ^5^Department of Wildlife, Fish, and Conservation Biology, University of CaliforniaDavis, CA, USA

**Keywords:** salt handling, salt-responsive gene, differential expression, gene-environment interaction, mRNA sequencing

## Abstract

Despite recent success with genome-wide association studies (GWAS), identifying hypertension (HTN)-susceptibility loci in the general population remains difficult. Here, we present a novel strategy to address this challenge by studying salinity adaptation in the threespine stickleback, a fish species with diverse salt-handling ecotypes. We acclimated native freshwater (FW) and anadromous saltwater (SW) threespine sticklebacks to fresh, brackish, and sea water for 30 days, and applied RNA sequencing to determine the gene expression in fish kidneys. We identified 1844 salt-responsive genes that were differentially expressed between FW sticklebacks acclimated to different salinities and/or between SW and FW sticklebacks acclimated to full-strength sea water. Significant overlap between stickleback salt-responsive genes and human genes implicated in HTN was detected (*P* < 10^−7^, hypergeometric test), suggesting a possible similarity in genetic mechanisms of salt handling between threespine sticklebacks and humans. The overlapping genes included a newly discovered HTN gene—*MAP3K15*, whose expression in FW stickleback kidneys decreases with salinity. These also included genes located in the GWAS loci such as *AGTRAP*-*PLOD1* and *CYP1A1*-*ULK3*, which contain multiple potentially causative genes contributing to HTN susceptibility that need to be prioritized for study. Taken together, we show that stickleback salt-responsive genes provide valuable information facilitating the identification of human HTN genes. Thus, threespine sticklebacks may be used as a model, complementary to existing animal models, in human HTN research.

## Introduction

Hypertension (HTN), or the chronic elevation of blood pressure (BP), is a major human health problem. The pathophysiology of HTN is complex, and multiple potential mechanisms are likely to contribute to the development of higher BP. Identifying genetic loci associated with HTN or BP regulation in the general population has proved to be challenging (Dominiczak and Munroe, 2010; Hastie et al., 2010; Padmanabhan et al., 2012). Genome-wide association studies (GWAS) of HTN have had varying degrees of success (Levy et al., 2009; Newton-Cheh et al., 2009; International Consortium for Blood Pressure Genome-Wide Association Studies, 2011). Genetic variants identified so far explain only a small portion of the heritability of susceptibility to HTN.

Dietary salt appears to be an important environmental factor in raising BP. The association between a high salt intake and high BP has long been known (Guyton et al., 1972; Luft and Weinberger, 1982; Haddy and Pamnani, 1995; Frisoli et al., 2012). Modern humans have the tendency to consume salt in excess—today, almost unanimously worldwide, the average individual's dietary salt intake largely exceeds its physiological need (McCarron et al., 2013). Chronic excess salt intake results in the development of HTN in the general human population (Kotchen et al., 2013). Numerous epidemiologic, clinical, and experimental studies have shown that a reduction in dietary salt intake lowers BP (Luft and Weinberger, 1982; Haddy and Pamnani, 1995; Frisoli et al., 2012; Kotchen et al., 2013). In some populations with very low salt intake, such as Papua New Guineans and Yanomamo Indians in the Amazon region, HTN and age-related increases in BP are virtually absent (Denton, 1982).

A general increase in BP in response to salt intake is almost assured, whilst the degree of the response is quite variable among individuals. This variation (or salt sensitivity) depends on the functional interactions among genes that play a role in salt handling (Meneton et al., 2005). Many of salt handling genes, such as those encoding molecules that control the ability of the kidney to maintain salt balance, are implicated in HTN (Lifton et al., 2001). Thus, a better understanding of the genetics of salt handling will facilitate identification of HTN and/or BP-regulating (HTN/BP) genes. To this end, we present a new strategy that involves the use of the threespine stickleback, *Gasterosteus aculeatus*, to identify salt handling genes. Threespine stickleback is a short-lived fish species with diverse salt-handling ecotypes, commonly found off the Atlantic and Pacific coasts of North America. The retreat of glaciers at the end of the last Ice Age resulted in a large number of new freshwater lakes and streams throughout the Northern hemisphere. Marine sticklebacks colonized and adapted to these newly formed freshwater habitats (Jones et al., 2012). Owing to its well-studied natural history and extensive genetic resources, threespine stickleback has been a powerful model for studying genotype-environment (GxE) interactions and adaptive evolution (McCairns and Bernatchez, 2010; Barrett et al., 2011; Grøtan et al., 2012; Jones et al., 2012).

The rationale for our strategy is based on extensive previous findings in fish genetics research. Salinity tolerance—critical for aquatic organisms to manage the osmotic challenges of the medium—is genetically determined (Rengmark et al., 2007; McCairns and Bernatchez, 2010; Le Bras et al., 2011; Norman et al., 2011). Gene expression changes contribute to the adaptation of fish to different environmental salinity (Scott et al., 2004; Bystriansky et al., 2006; Niu et al., 2008; Whitehead et al., 2012; Kozak et al., 2014). Interestingly, *ATP2B1* (encoding a plasma membrane calcium-transporting ATPase), is known to be involved in salinity adaptation in fish (Rengmark et al., 2007). It is also the first gene identified in GWAS to be associated with HTN susceptibility in humans (Levy et al., 2009). Furthermore, genetic mechanisms underlying kit ligand (*Kitlg*) expression and ectodysplasin (*EDA*) signaling in threespine sticklebacks are highly similar to those in humans (Colosimo et al., 2005; Miller et al., 2007).

In the present study, we used RNA sequencing to determine gene expression levels in threespine sticklebacks acclimated to various salinities. We identified salt-responsive genes whose expression levels vary significantly across samples among different salinity treatment groups. We also showed evidence that these salt-responsive genes provide valuable information facilitating identification of putative human HTN/BP genes.

## Materials and methods

### Fish collection and acclimation

**Figure 1:**
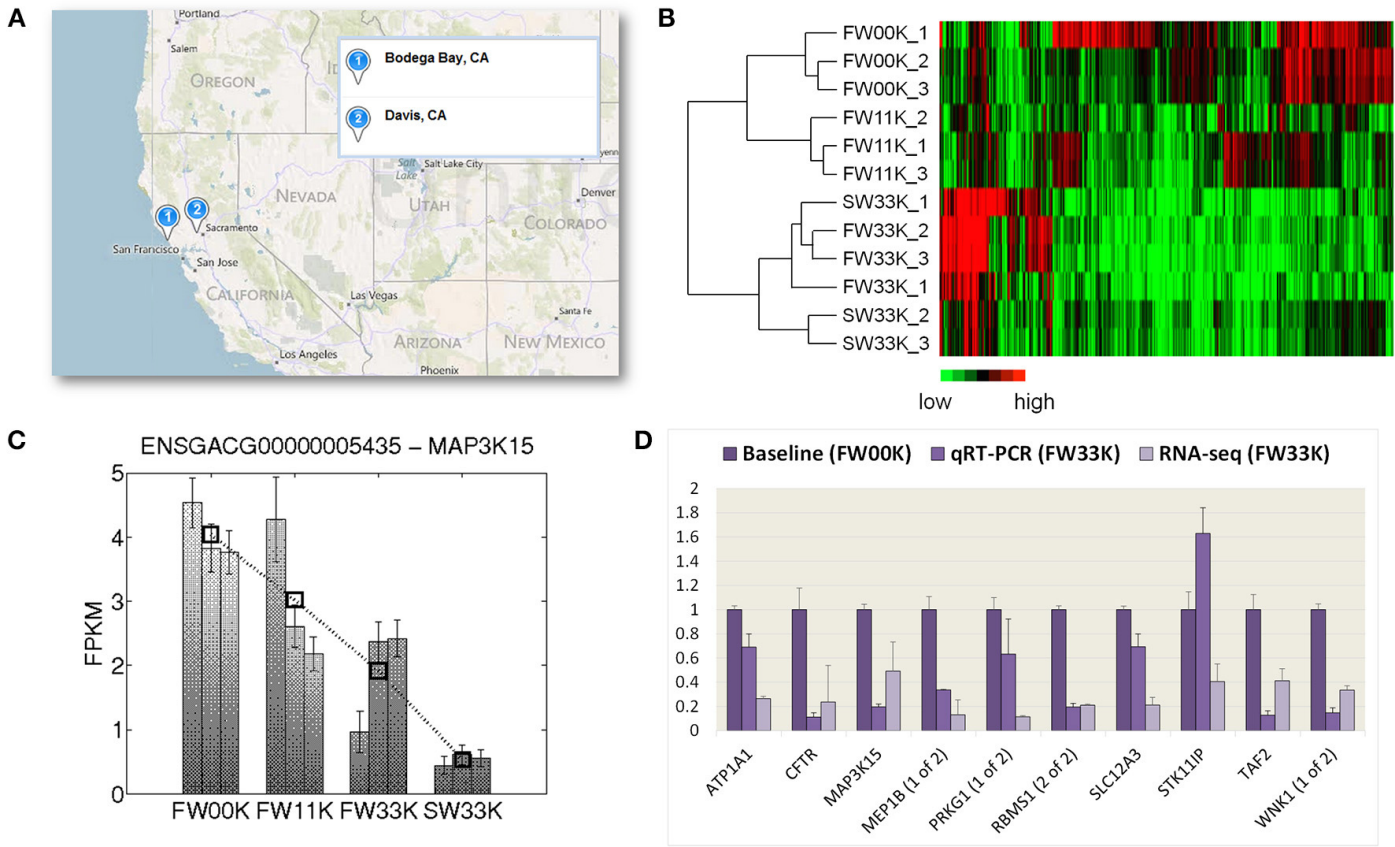
**Measuring gene expression in threespine stickleback kidneys**. **(A)** Sampling locations in Bodega Bay and Davis, California, USA. **(B)** Hierarchical clustering of expression profiles for 2000 most variably expressed genes in samples, with a heat map chart showing 500 genes. **(C)** Expression profile of *MAP3K15*. The expression levels of *MAP3K15* under the four acclimation conditions: FW00K, FW11K, FW33K, and SW33K, each with three replicates, are shown. The y-axis indicates the value of FPKM represented as the means ± s.e.m. The open square symbols indicate the mean FPKM across three replicates in same condition. The dash line links highlight the change in expression pattern of FPKM values across acclimation conditions. **(D)** RNA-seq and qRT-PCR results of relative expression differences (i.e., fold changes) between FW00K and FW33K for 10 select genes.

In spring 2012, freshwater (FW) and saltwater (SW) threespine sticklebacks were collected using Frabill galvanized minnow traps from a freshwater lake in Davis, CA (38°32′N, 122°12′W) and Bodega Bay, CA (38°19′N, 123°3′W), respectively (Figure 1A). Fish were transported in temperature-controlled coolers equipped with aeration devices to maintain oxygen levels at >95% saturation. Thirty adult females were randomly selected from each population and assigned into three groups. One group from each population was acclimated to fresh water (“zero salinity” treatment), the second group was acclimated to a salinity of 11 g/L (“mid salinity” treatment), and the third group was acclimated to a salinity of 33 g/L (“high salinity” treatment). All fish were acclimated for 30 days with no mortality. Male fish were excluded from experiments as their kidneys may undergo variable structural transformation during the breeding season (Ruiter and Bonga, 1985). To achieve the desired salinities, synthetic sea salt (Instant Ocean) was mixed with aerated well water; tank salinities were monitored with a calibrated light refractometer (Vita Sine) and adjusted daily to within 1 g/L of the desired level. Water temperature was held at 11 ± 0.5°C, the photoperiod set to 12 h day: 12 h night, and all fish were fed daily to satiation with frozen brine shrimp. Water quality parameters such as oxygen, ammonia, nitrate, and nitrite were monitored daily and adjusted through water changes to keep these parameters within optimal husbandry limits for all fish. All fish were treated in accordance with UC Davis Institutional Animal Care and Use Committee guidelines (protocol #16474). At the end of acclimation, fish were anesthetized with a lethal dose of buffered tricaine mesylate (MS-222). Kidneys were dissected following standard sampling techniques for fish gene expression studies and immediately frozen at −80°C. We processed kidney tissues obtained from four (out of six) treatments: (1–3) FW fish acclimated to fresh, brackish, and sea waters, labeled as FW00K, FW11K, and FW33K, respectively, and (4) SW fish acclimated to sea water, SW33K. Three individual fish (biological replicates) were included per treatment.

### RNA extraction and sequencing

Total RNA was extracted from each fish's kidneys using the column-based RNA extraction kit (Qiagen, Venlo, Netherlands). RNA integrity was assessed by Agilent Bioanalyser 2100 and RNA Nano 6000 Labchip kit (Agilent Technologies, Palo Alto, USA). All samples were concentrated and cleaned using the RNAeasy MiniElute Cleanup kit (Qiagen) obtaining final concentrations ~500 ng/μl. Sequencing libraries were produced using the Illumina mRNA sequencing sample preparation kit (Illumina, San Diego, USA), following the manufacturer's instructions. Briefly, 4 μg of total RNA was used as input for poly A+ selection, followed by metal-catalyzed fragmentation of the selected mRNA (peak of size distribution at ~240 nt). After reverse transcription to cDNA using random hexamer primers, end-repair and A-tailing of the double stranded cDNA was performed. The cDNA was then ligated to indexed pairs of adapters. The cDNA was size selected on a 2% agarose gel, and fragments corresponding to an insert size of 150 nucleotides were excised from the gel. The cDNA was recovered from the gel slice using a QIAquick gel extraction kit (Qiagen). Thereafter, the libraries were amplified in 10 cycles of PCR, quantified using Taqman, and adjusted at a concentration of 10 pM. The sequencing of 100 bp paired-end reads was carried out on the Illumina HiSeq 2000 platform at the Texas A&M AgriLife Genomics and Bioinformatics Services. All cDNA samples were individually barcoded, and every four samples were pooled and sequenced on the same sequencing lane.

### Identification of salt-responsive genes

The reference sequences of the stickleback genome (BROADS1) and the gene annotation were downloaded from the Ensembl database (Flicek et al., 2014). The annotation information included the stickleback gene set built using a modified version of the standard Ensembl genebuild pipeline, and the stickleback-human orthologs predicted using a phylogenetic approach (Vilella et al., 2009). Prior to the mapping, we processed the reference genome by masking all nucleotides at positions known to be polymorphic in threespine sticklebacks. More specifically, we replaced nucleotides of the reference genome sequences at 5.9 million polymorphic sites discovered by Jones et al. (2012) with “N” (indicating any nucleotides).

The quality control analysis on raw sequence data was done by using FastQC (http://www.bioinformatics.babraham.ac.uk/projects/fastqc/). To clean the raw sequences, we removed low-quality reads that contained base(s) with a quality score less than 20. We then trimmed all remaining reads using the FASTX toolkit (http://hannonlab.cshl.edu/fastx_toolkit/). The bases at positions 1–10 and 86–100 were trimmed, leaving 75 base pairs for each read. The clean reads were aligned to the processed reference genome using TopHat v2.0.3 (Trapnell et al., 2012). The default set of TopHat options was used, except that read-mismatches (mismatches allowed in final read alignments) was set to 2 and 3 for FW and SW sticklebacks, respectively. Cufflinks v2.0.2 was used to estimate the expression levels of annotated stickleback genes in fragments per kilobase of exon model per million mapped reads (FPKM). Cufflinks option GTF-guide was switched on to allow the algorithm to use the supplied reference annotation to guide assembly and make the output include all reference transcripts. SAMMate (Xu et al., 2011) was used to obtain the count of reads mapped onto each gene from the SAM files generated by TopHat. Three R packages for differential expression analysis: edgeR (Robinson et al., 2010), DESeq (Anders and Huber, 2010), and baySeq (Hardcastle and Kelly, 2010), were applied independently to the same input file containing the number of mapped reads for all genes. The results were jointly evaluated to identify differentially expressed genes. More specifically, we considered a gene to be significantly differentially expressed between any two treatments only when all three programs reported that the expression difference was significant at the threshold of FDR < 0.01.

### Quantitative RT-PCR

RNA was extracted using Trizol solution (Life Technologies, Waltham, USA), treated with DNaseI using the DNA-free kit (AMS Biotechnology) and quantified spectrometrically. The cDNA was synthesized from 3 μg of RNA using the AffinityScript QPCR cDNA Synthesis Kit (Agilent Technologies) according to the manufacturer's instructions. Briefly, the required amount of RNA (up to 3 μg) was diluted in RNase-free water (up to 7 μl final volume) and mixed on ice with 1 × cDNA Synthesis master mix (10 μl), random primers:oligo-dT primers (3:1) [total, 2 μl (200 ng)], and either 1 μl of reverse transcriptase/RNase block enzyme mixture for reverse transcription reactions or 1 μl of water for control reactions. Reaction mixtures were mixed and spun down and incubated for 2 min at 25°C, 40 min at 42°C, and 5 min at 95°C. cDNA was stored at −20°C. Dilutions of this cDNA were used subsequent real-time PCR reactions. Quantitative RT-PCR reactions were performed with designed exonic primers for selected stickleback genes (Supplementary Table 1) and the amount of cDNA was quantified using SYBR Green real-time PCR (Life Technologies) on a 7900HT Fast real time PCR system (Life Technologies). The PCR 2× master mix was based on AmpliTaq Gold DNA polymerase (Life Technologies). In the same reaction, cDNA samples (5 μl for a total volume of 25 μl per reaction) were analyzed, cycle temperatures and times were according to the manufacturers' protocols (Life Technologies). Data was analyzed using ΔΔC_T_ method implemented in the software qbasePLUS (Biogazelle, Zwijnaarde, Belgium). All reactions were run in triplicate and normalized by comparisons to the reference gene *GAPDH* (ENSGACG00000010219) (Pei et al., 2007).

### Compiling list of HTN/BP genes

We compiled a list of HTN/BP genes identified using non-GWAS approaches. The sources of this gene list included the literature review by Hancock et al. (2008) and the Genetic Association Database (Becker et al., 2004). We also compiled a list of HTN/BP genes identified using the GWAS approach through searching the Catalog of Published GWAS (http://www.genome.gov/gwastudies/) and the original literature (Levy et al., 2009; Newton-Cheh et al., 2009; Ho et al., 2011; International Consortium for Blood Pressure Genome-Wide Association Studies, 2011; Kato et al., 2011; Wain et al., 2011).

### Testing significance of gene overlap

To assess the significance of overlap between stickleback salt-responsive genes and human HTN/BP genes, we focused on 15,029 stickleback protein-coding genes, of which human orthologs exist. The probability of overlap was calculated with the hypergeometric probability density function $f(k)\;=\;\frac{\left(\begin{array}{c} m\\ k \end{array}\right)\left(\begin{array}{c} N-m\\ n-k \end{array}\right)}{\left(\begin{array}{c} N\\ n \end{array}\right)}$, where *N* (=15,029) is the number of all stickleback genes considered, *m* (=1302) is the number of salt-responsive stickleback genes whose human orthologs exist, and *n* (=455) is the number of stickleback genes whose human orthologs are HTN/BP genes, and *k* (=75) is the number of salt-responsive stickleback genes whose human orthologs are HTN/BP genes. The computation was done using 1-hygecdf(*k*,*N*,*m*,*n*) in Matlab.

### Data accessibility

Expression and sequence data have been deposited at the Gene Expression Omnibus (GEO) under accession GSE58447 and Sequence Read Archive (SRA) under accession SRP043184.

## Results

Using high-throughput sequencing, we obtained over 25 million paired-end 100-bp reads for each RNA sample of the four acclimation treatments: FW00K, FW11K, FW33K, and SW33K (Supplementary Table 2). The expression levels for all annotated stickleback protein-coding genes were quantified, and a hierarchical clustering analysis was performed with the 2000 most variably expressed genes across treatments, showing that expression profiles of these genes were largely clustered by acclimation treatment (Figure 1B). To identify differentially expressed genes, we applied a rather conservative criterion, which was based on completely cross-validated results from three different statistical tests, to assess the significance of gene expression differences between acclimation treatments. A gene was considered to be salt-responsive when the expression of the gene is significantly different between FW treatments (e.g., FW00K vs. FW11K or FW00K vs. FW33K) or between FW33K and SW33K. Using this definition, we identified 1844 stickleback salt-responsive genes (Supplementary Table 3), of which 1302 have human orthologs. This list contains a number of genes such as ion transporting ATPases (*ATP1A1, ATP1A2, ATP1B1, ATP2A2*, and *ATP5B*), aquaporin (*AQP4*), and transmembrane emp24 domain trafficking protein 2 (*TMED2*), which are known to be involved in the salinity response and osmoregulation in other fish species (Rengmark et al., 2007; Whitehead et al., 2011; Lamichhaney et al., 2012). An online interactive resource (http://stickleback.genomezoo.net) was created to allow easy navigation of the expression profiles of all genes. The expression levels of *MAP3K15* in four acclimation treatments is shown in Figure 1C as an example. Finally, for 9 out of 10 selected genes, the expression level in FW33K relative to FW00K was confirmed by quantitative RT-PCR (Figure 1D).

### Significant overlap between salt-responsive genes and HTN/BP genes

To examine the overlap between stickleback salt-responsive genes and human HTN/BP genes, we compiled the list of HTN/BP genes including 560 identified using non-GWAS approaches (e.g., the single association analyses, physiology studies, and animal model studies) (Supplementary Table 4) and 108 identified using GWAS approach (Supplementary Table 5) (Levy et al., 2009; Newton-Cheh et al., 2009; Ho et al., 2011; International Consortium for Blood Pressure Genome-Wide Association Studies, 2011; Kato et al., 2011; Wain et al., 2011). Overlap analysis identified 75 stickleback salt-responsive genes whose human orthologs are HTN/BP genes. This overlap is significantly higher than expected by random chance (hypergeometric test: *P* = 1.6×10^−8^), suggesting a striking similarity in the genetics of salt handling between threespine sticklebacks and humans. The 75 overlapping genes included 65 non-GWAS and 10 GWAS HTN/BP genes (Tables 1, 2).

**Table 1:** **Representative stickleback salt-responsive genes whose human orthologs are HTN/BP genes identified by non-GWAS approaches**.

**Ensembl ID**	**Gene symbol**	Type of study^*^			**Expression level (FPKM)**			
		**1**	**2**	**3**	**FW00K**	**FW11K**	**FW33K**	**SW33K**
ENSGACG00000009898	*ACE*	X			46.83	29.60	6.49	15.55
ENSGACG00000002433	*APOE* (2 of 2)	X			0.35	0.81	8.42	1.22
ENSGACG00000018525	*AR* (2 of 2)		X		48.81	58.37	84.95	163.23
ENSGACG00000012346	*ARG2*	X			21.34	16.39	6.91	2.41
ENSGACG00000008681	*AVPR2* (1 of 2)		X		3.86	2.42	24.89	7.65
ENSGACG00000006921	*CXCL12* (2 of 2)	X			1143.59	1247.81	304.94	42.77
ENSGACG00000015943	*CYBA*	X			178.56	362.39	53.47	19.60
ENSGACG00000014669	*CYP4F2*	X		X	116.70	144.07	42.90	10.55
ENSGACG00000007514	*ESR2*	X			6.71	11.00	22.97	18.01
ENSGACG00000018868	*GCGR* (2 of 2)		X		6.30	1.00	0.51	0.42
ENSGACG00000006771	*HMOX1* (2 of 2)		X		32.18	37.77	3.98	3.80
ENSGACG00000004566	*MACROD2*		X		19.45	18.99	33.28	32.25
ENSGACG00000008374	*MAT2A* (1 of 2)			X	57.55	80.23	813.40	244.27
ENSGACG00000015329	*MTR*	X	X		7.78	5.95	5.83	26.86
ENSGACG00000008228	*P2RY2* (2 of 2)		X		7.58	9.33	11.29	16.24
ENSGACG00000008313	*PDGFB* (1 of 2)	X		X	1.82	1.45	23.24	8.56
ENSGACG00000003693	*PLOD2*			X	9.96	5.64	1.62	12.76
ENSGACG00000018958	*PPARA* (2 of 2)	X			36.07	14.97	5.86	4.21
ENSGACG00000009151	*PRKG1*	X		X	17.21	5.22	1.97	2.91
ENSGACG00000014838	*PTK2B* (2 of 2)		X		18.05	24.92	5.91	3.01
ENSGACG00000019365	*SHBG*			X	9.60	1.01	0.59	1.08
ENSGACG00000007570	*SLC6A19* (1 of 3)		X		26.92	13.19	1.03	14.09
ENSGACG00000020600	*SLC7A1* (1 of 2)		X		13.95	4.66	1.03	2.62

*Ensembl gene identifiers of stickleback genes and HGNC approved symbols of human orthologous genes are given. “X” indicates what type of study has been performed and discovered the corresponding HTN/BP gene. The types of studies are categorized based on the methodology applied to the research*.

* *Explanation coding: 1-physiology or drug target study; 2-single or meta-association analysis for common phenotype; 3-animal model study*.

**Table 2:** **Stickleback salt-responsive genes whose human orthologs are HTN/BP genes identified by GWAS**.

**Ensembl ID**	**Gene symbol**	**GWAS locus**	**Expression level (FPKM)**			
			**FW00K**	**FW11K**	**FW33K**	**SW33K**
ENSGACG00000014674	*CYP1A2*	*CYP1A1-ULK3*	32.57	5.55	2.73	20.30
ENSGACG00000001963	*ENPEP*	*–*	24.80	10.19	53.79	91.52
ENSGACG00000011478	*FURIN* (2 of 2)	*FURIN-FES*	2.82	1.19	0.30	0.38
ENSGACG00000014177	*GOSR2*	*–*	32.18	59.34	97.71	84.12
ENSGACG00000004493	*JAG1* (1 of 2)	*–*	10.84	4.36	1.83	4.38
ENSGACG00000016429	*LMAN1L*	*CYP1A1-ULK3*	10.20	21.16	33.68	34.63
ENSGACG00000012633	*MTHFR*	*MTHFR(5′)-NPPB*	14.96	11.32	6.20	24.10
ENSGACG00000012162	*PGR*	*FLJ32810-TMEM133*	3.90	0.34	0.49	0.24
ENSGACG00000006801	*PLCE1*	*–*	5.20	1.15	0.84	1.79
ENSGACG00000015632	*PLEKHA7* (1 of 2)	*–*	7.54	3.09	5.70	3.62

### Prioritizing genes in GWAS locus with multiple potentially causative genes

Many genes identified in GWAS had no obvious mechanistic link with HTN or BP regulation. For example, despite being identified by GWAS, *GOSR2* had not been previously suspected to regulate BP (International Consortium for Blood Pressure Genome-Wide Association Studies, 2011). The result of gene overlap testing suggested that the stickleback salt-responsive genes might be used to prioritize HTN/BP genes. This will be extremely useful for prioritizing genes in GWAS loci that contain multiple potential causative genes contributing to the overall HTN susceptibility. For example, the *AGTRAP*-*PLOD1* locus has been associated with BP regulation in several independent studies (Levy et al., 2009; Newton-Cheh et al., 2009) (see Flister et al., 2013, for a complete list of related references). This locus with high genetic complexity contains six genes: *AGTRAP, MTHFR, CLCN6, NPPA, NPPB*, and *PLOD1*, among which three (*MTHFR, CLCN6, PLOD1*) have corresponding one-to-one orthologs between human and stickleback. Our results showed that *MTHFR* is a salt-responsive gene in stickleback, showing significant differential expression between FW33K and SW33K. The other two genes, *CLCN6* and *PLOD1*, albeit not classified as salt-responsive genes, also show a certain degree of differential expression across acclimation treatments (Supplementary Figure 1). In other words, the pattern of gene expression response to salinity suggested that the three genes may be implicated in HTN and BP regulation. Indeed, Flister et al. (2013) used the zinc-finger nuclease-based mutagenesis procedure to introduce damaged alleles into each of the six genes at the *AGTRAP*-*PLOD1* locus in a mice model of HTN. They compared the mutant mice with wild-type littermates, all on a high salt diet, and found that *CLCN6* and *PLOD1* mutants showed significantly higher BP, while *MTHFR* mutants showed increased urinary protein excretion, than the wild-type. Thus, all three genes have confirmed roles in the HTN pathogenesis.

**Figure 2:**
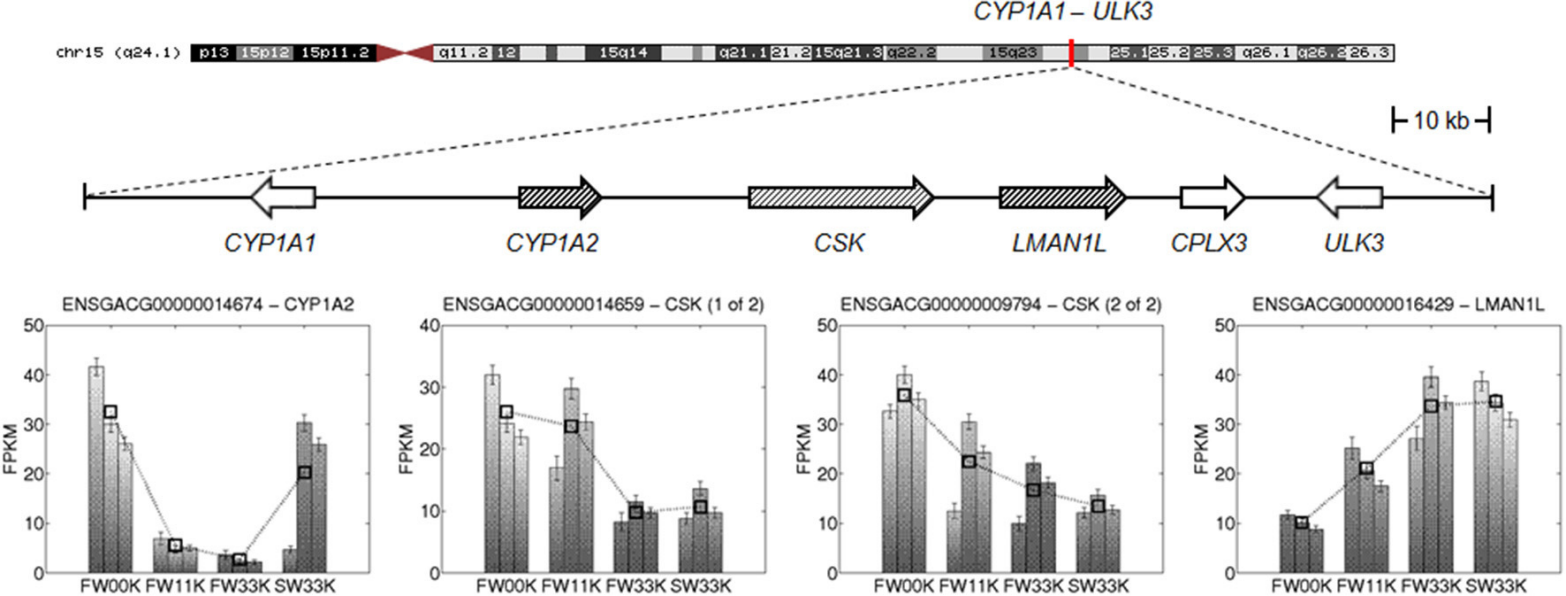
**Salt-responsive genes in a multigene locus**. *CYP1A1*-*ULK3* is a multigene locus associated with BP regulation, reported by GWAS (International Consortium for Blood Pressure Genome-Wide Association Studies, 2011). Two salt-responsive genes, *GYP1A2* and *LMAN1L*, are indicated with hashed arrows. *CSK*, whose expression apparently changes in response to salinity, is also highlighted. Expression profiles of genes are given in the bar plots.

In another example, genetic variants at the locus *CYP1A1*-*ULK3* in a genomic region spanning a 150-kb interval on chromo-some 15q24.1 have been associated with both systolic and diastolic BP (International Consortium for Blood Pressure Genome-Wide Association Studies, 2011). However, without any further information, it is difficult to predict which genes, among the total six within the region, are most relevant to BP regulation. We found that two genes in the region, *GYP1A2* and *LMAN1L*, are salt-responsive genes that showed decreased and increased gene expression with the increase of salinity, respectively (Figure 2). Thus, these two genes are more likely to be functionally implicated in HTN pathogenesis compared with other genes in the locus.

### Impact of salinity on BP regulatory genetic network

**Figure 3:**
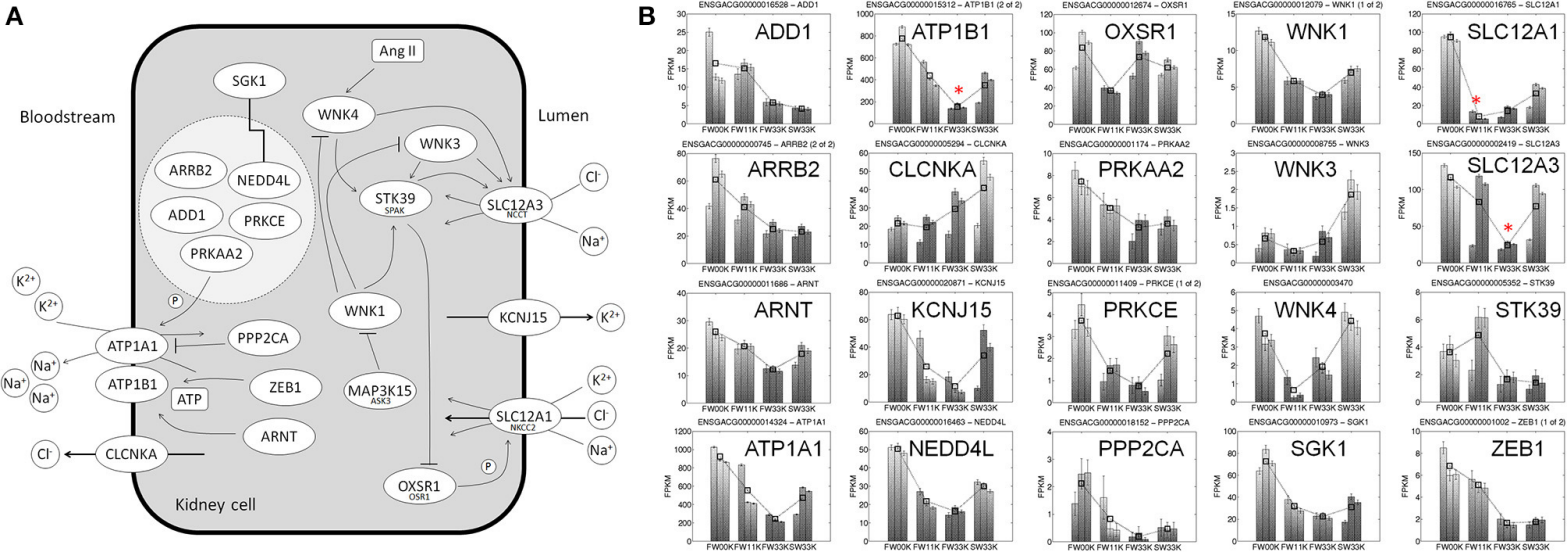
**Expression profiles for stickleback genes in diuretics pathway**. **(A)** Diagrammatic representation of the diuretic pathway. **(B)** Expression profiles of genes in the diuretic pathway. For each gene, expression levels of the gene under four acclimation treatments, FW00K, FW11K, FW33K, and SW33K, are shown in the bar plots.

BP is regulated by an intricate network of physiological and genetic pathways involving extracellular fluid volume homeostasis (Meneton et al., 2005). We found that the expressions of 20 stickleback genes mapped onto the same diuretic pathway were perturbed simultaneously by salinity. The expression changes of these genes were largely orchestrated in response to the salinity changes (Figure 3). Notably, the expressions of *SGK1* and *ATP1A1* were monotonically decreased with the increase of salinity. This is expected from the known function of these two genes: *SGK1* is a major kinase that regulates Na^+^ intake by phosphorylation of epithelial sodium channels (ENaCs) (Wulff et al., 2002), and *ATP1A1* encodes the catalytic subunit α1 of Na^+^/K^+^-ATPase. *ATP1A1* is known to be responsible for freshwater adaptation in threespine sticklebacks (McCairns and Bernatchez, 2010), as well as the pathogenesis of a common subtype of adrenal HTN in humans (Azizan et al., 2013). Several other examples suggest that some aspects of the effect of salinity on gene expression are transferable from sticklebacks to mammals. *WNK4* is a salt-responsive gene in sticklebacks, and the *WNK4* transgenic mice exhibit hypertensive phenotypes (Ohta et al., 2009). *WNK3* is not a salt-responsive gene in sticklebacks, and *WNK3* knockout (KO) mice do not exhibit hypertensive phenotypes (including normal expression of kidney epithelial Na^+^ channels, Na^+^-H^+^ exchangers, and urine Na^+^ and K^+^ excretion) (Oi et al., 2012). Furthermore, in mammals, *CLCNKA, SLC12A1*, and *SLC12A3* are known to be involved in regulating osmotic balance between blood and the lumen of renal corpuscle. In stickleback kidneys, we found that *CLCNKA* was down-regulated, and *SLC12A1* and *SLC12A3* up-regulated, with salinity. This difference in the direction of regulation may be due to the involvement of *CLCNKA* in reabsorption of Cl^−^ from kidney cells to blood, whereas *SLC12A1* and *SLC12A3* are involved in reabsorption of NaCl from blood to kidney cells (Figure 3).

### Candidate HTN/BP genes in the salt-responsive gene list

The association of many stickleback salt-responsive genes with HTN and BP regulation is currently unknown. The salt-responsive genes, however, are likely to be implicated in HTN and BP regulation. To this end, we used the gene *MAP3K15* to illustrate this point. *MAP3K15* is a newly characterized homolog of apoptosis signal-regulating kinase 1 and 2 (*MAP3K5* and *MAP3K6*). All three are members of the mitogen-activated protein kinase kinase kinase (MAP3K) family, broadly implicated in apoptotic cell death, stress responses, and various diseases. *MAP3K15* is predominantly expressed in mammalian kidneys. In stickleback kidneys, the gene expression of *MAP3K15* differed significantly between FW00K and FW33K (cuffdiff FDR < 0.01). Expression of *MAP3K15* in FW00K, FW11K, and FW33K showed a significant, negative linear relationship against salinity (*P* < 0.001, significant test for linear regression; Figure 1C). The molecular function of *MAP3K15* in stickleback is unknown, but we assumed that it is the same as it is in mammalian kidneys. Recently, Naguro et al. (2012) found that *MAP3K15* is expressed in the epithelium of the renal tubules in mouse kidney. They monitored the kinase activity of endogenous *MAP3K15* in human HEK293A cells after gradually altering the concentration of NaCl by 10–mM steps or mannitol by 20–mM steps in extracellular solutions. They found that the kinase activity of *MAP3K15* was affected in both directions by a 10–20 mOsm change around the isotonic condition. They also found that knockdown of *MAP3K15*, by short interfering RNA, enhanced the activation of the WNK1-SPAK/OSR1 signaling pathway. Moreover, *MAP3K15* KO mice exhibited a hypertensive phenotype: the systolic BP of KO mice increased significantly along with aging (Naguro et al., 2012).

## Discussion

It has long been suggested that studying adaptive traits of threespine sticklebacks may be informative to understanding general metabolism and physiology in other animals including humans (Kingsley, 2003). In the present study, we adopt the idea of using threespine sticklebacks as a GxE animal model to facilitate the search of genes underlying human susceptibility to HTN and BP regulation. Despite the fact that humans and threespine sticklebacks come from different phylogenetic lineages, genetic mechanisms for coping with similar physiological challenges are likely to be shared (Kingsley, 2003; Jones et al., 2012). Indeed our results showed that there is a considerable degree of similarity in genetics of salt handling between threespine sticklebacks and humans.

### Threespine sticklebacks as a model complementary to rodent models of HTN

The genetic similarities between human and stickleback and the osmoregulationary mechanisms broadly conserved in vertebrates have prompted us to suggest that threespine sticklebacks may be used as a complementary model for HTN research. Today, the spontaneously hypertensive rat (SHR) and the Dahl salt-sensitive rat (DSR) are among the most extensively used animal models for HTN (Rapp, 1982; Pravenec and Kurtz, 2010). However, there are inherent difficulties of identifying causative mutations in both SHR and DSR systems (Pravenec and Kurtz, 2010). Several distinctive features make threespine sticklebacks a valuable model complementary to SHR and DSR. First, owing to the existence of SW and FW subspecies of threespine sticklebacks, it is possible to investigate genetic effects through contrasting long-term evolution and shorter-term adaptation. To be specific, FW threespine sticklebacks are derived from SW ancestors, which had been subject to selection over recent evolutionary timescales (e.g., ~10,000–12,000 years ago) (Jones et al., 2012). A large number of derived mutations with functional implications have become fixed in FW threespine sticklebacks. These genetic variants can be identified by comparative genomic studies (Jones et al., 2012) and the impact of these genetic variants on gene expression can be studied through comparative transcriptomics. The differences in gene expression between FW and SW threespine stickleback subspecies acclimated under the same condition (e.g., gene expression differences between FW33K and SW33K) may be attributed to genetic diversity between the two subspecies. On the other hand, the difference in gene expression among individuals of the same subspecies acclimated to different salinities (e.g., FW00K vs. FW33K), may be attributed to short-term adaptive response, thus enabling the effect of an environmental factor to be dissected from the combined effect of both genetic and environmental factors. Second, compared with rodents, threespine sticklebacks are more distantly related to humans. Because of the phylogenetic distance, threespine sticklebacks can yield profound insight into biological processes involved in human diseases. Modeling diseases in distantly related organisms can reveal the complexity of human diseases and help uncover core defective processes (Lieschke and Currie, 2007; Albertson et al., 2009; McGurk and Bonini, 2011). Thus, our results may help to distinguish causal genetic variants from downstream complications through targeting the core problem that is more likely to arrest disease progression.

### Caveats and future directions

Additional measures could have been implemented to enhance our experimental results reported in the present paper. For example, using inbred fish lines might further reduce the influence of genetic diversity existing in the wild populations and mitigate residual environmental influence potentially acting on gene expression. More fish samples from the same location, or new fish samples from different geographic locations, could be included to generate greater statistical power in data analysis. Besides kidney, fish gut and gill also play dominant roles in osmotic and ionic regulation (Evans, 2008). Gene expression in gut and gill could be examined to give more comprehensive pictures of multi-tissue response to salinity. Nonetheless, our present study only focused on kidney because it plays a central role in maintaining appropriate sodium balance in humans and is critical for the determination of BP (O'Shaughnessy and Karet, 2006).

Although the majority of stickleback genes were not classified as salt-responsive genes by our definition, they should not be overlooked. Many of these “nonresponsive” genes in fact showed some degrees of expression changes in response to salinity. For example, neither copy of *NAFT5* (or *TonEBP*) was identified as salt-responsive gene; however, in FW stickleback kidneys, the expression of both copies decreased with salinity (Supplementary Figure 2). *NFAT5* is a Rel homology transcription factor classically known for its osmosensitive role in regulating cellular homeostasis during states of hypo- and hypertonic stress (Halterman et al., 2012; Cheung and Ko, 2013). Most recently, it was found that high-salt conditions activate the p38/MAPK pathway involving *NFAT5* and *SGK1* during cytokine-induced T_H_17 cell polarization, suggesting that dietary salt may influence autoimmune disease in humans through T-cell-induced production of IL-17 (Kleinewietfeld et al., 2013; O'Shea and Jones, 2013; Wu et al., 2013).

In conclusion, through examining the changes of threespine stickleback transcriptome in response to different salinities, we identified stickleback salt-responsive genes and detected significant overlap between these genes and human HTN/BP genes, suggesting the existence of common genetic regulatory mechanisms underlying fish salinity response and human HTN pathogenesis. Several new candidate genes for understanding HTN and BP regulation were identified along this line. Thus, we demonstrated that the salt-responsive genes identified through the stickleback acclimation experiments represent a valuable resource for fish genetics as well as human HTN research.

### Conflict of interest statement

The authors declare that the research was conducted in the absence of any commercial or financial relationships that could be construed as a potential conflict of interest.
