# Supplementary material for: Gene expression responses of threespine stickleback to salinity: implications for salt-sensitive hypertension
Source: Front Genet. 2014 Sep 11;5:312. doi: 10.3389/fgene.2014.00312 (PMC4160998; doi:10.3389/fgene.2014.00312)
Supplement: Supplementary file 1 [file DataSheet1.PDF]

Supplementary Figure 1.

Expression profiles of *CLCN6, MTHFR* and *PLOD1* in threespine stickleback kidneys in four acclimation treatments. These three genes have one-to-one orthologs between human and threespine stickleback. They are among the six genes at the *AGTRAP-PLOD1* locus (*AGTRAP, MTHFR, CLCN6, NPPA, NPPB,* and *PLOD1*) with high genetic complexity have been associated with BP and renal phenotypes by multiple independent study in the human population.

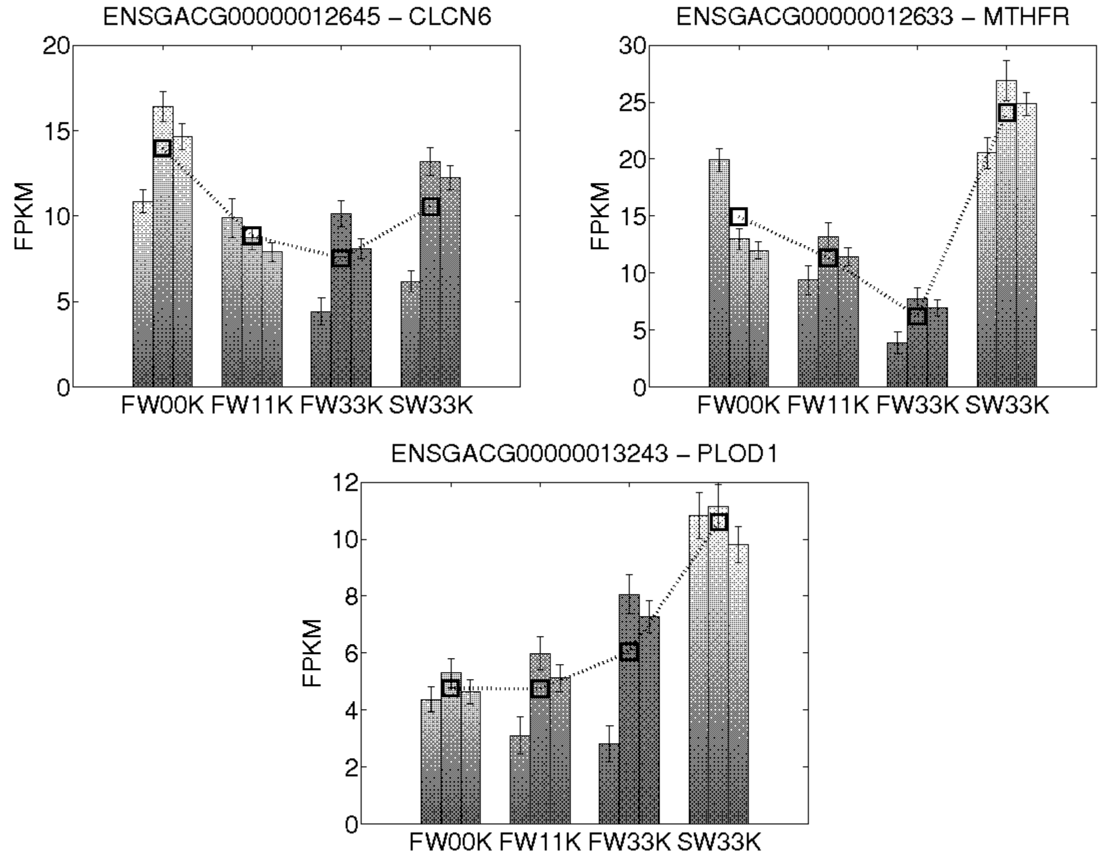

Supplementary Figure 2.

Expression profiles of *NFAT5* and *SGK1* in threespine stickleback kidneys in four acclimation treatments. Two paralogous threespine stickleback genes of *NFAT5* are shown. The schematic showing putative functional relations between 'NFAT5 gene' and 'Hypertensive disease' are produced using BioGraph (http://biograph.be).

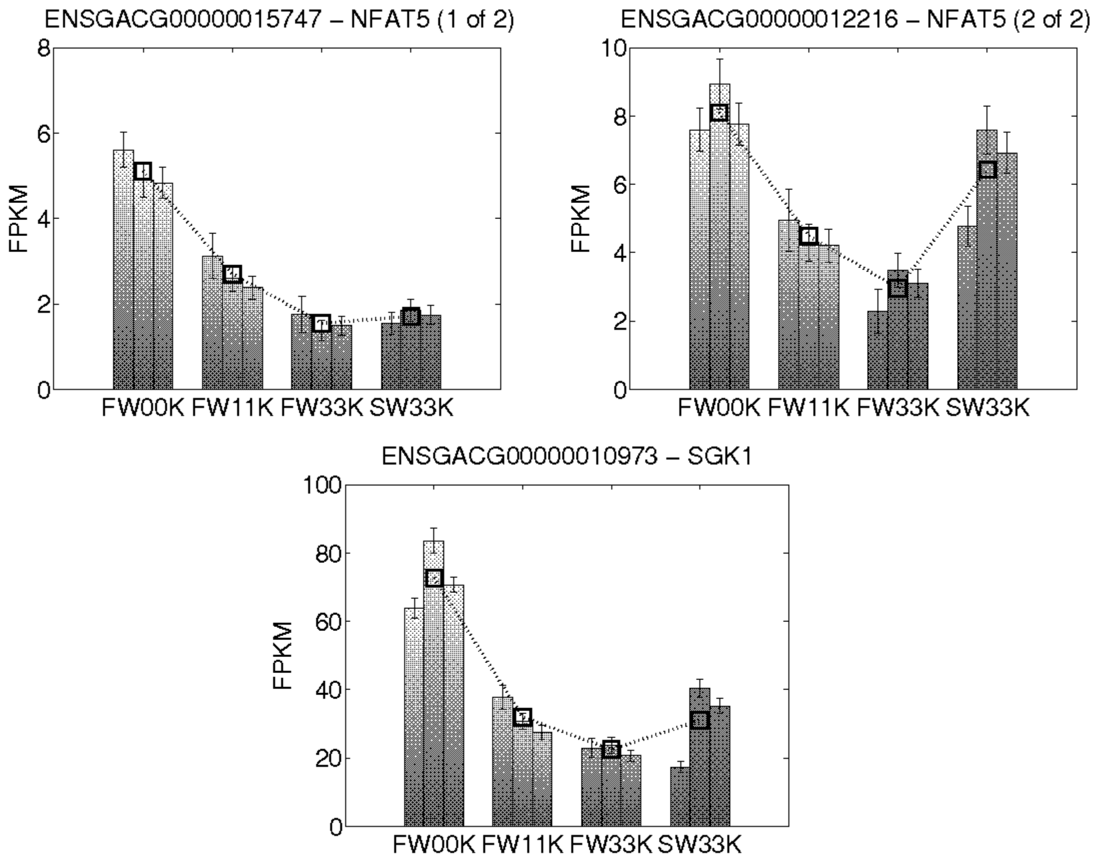

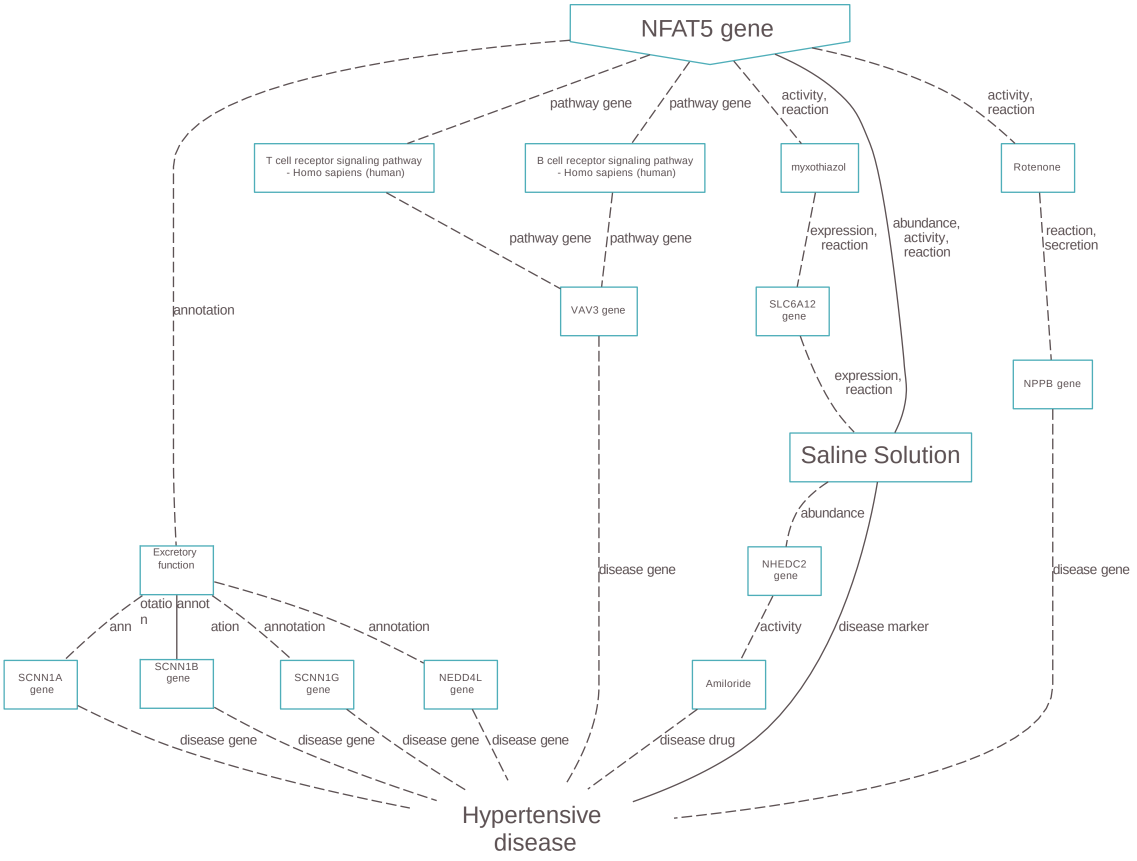
NFAT5 gene
pathway gene
pathway gene
activity, reaction
activity, reaction
T cell receptor signaling pathway - Homo sapiens (human)
B cell receptor signaling pathway - Homo sapiens (human)
myxothiazol
Rotenone
pathway gene
pathway gene
expression, reaction
abundance, activity, reaction
reaction, secretion
annotation
VAV3 gene
SLC6A12 gene
expression, reaction
NPPB gene
Saline Solution
abundance
Excretory function
disease gene
NHEDC2 gene
disease gene
otatio n
annot
ann
ation
annotation
annotation
activity
disease marker
SCNN1A gene
SCNN1B gene
SCNN1G gene
NEDD4L gene
Amiloride
disease gene
disease gene
disease gene
disease gene
disease drug
Hypertensive disease

Supplementary Table 1.
Oligonucleotide primers used for quantitative RT-PCR analysis.

Gene	Transcript Ensembl ID	Forward primer sequence (5'->3')	Reverse primer sequence (5'->3')
ATP1A1	ENSGACT00000018949	ACCGGCCAACGATAACTTGTACCT	AGAAGGCAATGTTCCTGGTCTCCA
CFTR	ENSGACT00000011967	AGAAGTTGGAGCTGACGGATGTGT	ACGCCTTCACAGAGTGGATGTTCT
MAP3K15	ENSGACT00000007208	GAGGCCAAGTCGTTCATCTT	CTTCTTGCCTTTGCTGTTCTG
MEP1B (1 of 2)	ENSGACT00000017226	ACGTTGGGAAGCAGAGATTG	GGGACTGTTCATGCCAGAAA
PRKG1 (1 of 2)	ENSGACT00000012119	TTCGCCAAGAAGATCGGATTC	GAGATGTCATGGCCCTTGTT
RBMS1 (2 of 2)	ENSGACT00000020055	ACGGCTTTGTGGACTTTGACAACC	ACACTGCTCTGTTGTGTCCATCCT
SLC12A3	ENSGACT00000003177	CTGTGTGGTCCTCATGTTCTT	GGCTTCTTGTAGACCGTGTATC
STK11IP	ENSGACT00000018903	GTGGGTGTGCTCTCTAACAAA	ACACCTCGCTTCTCCTCTTA
TAF2	ENSGACT00000005930	ACATGTGGAGCCAGATGCTTCTCT	TGAGGACGTCTTTGGGACAAAGGT
WNK1 (1 of 2)	ENSGACT00000016033	AGTTCAGGACGACATTGAAGAG	AAGGAGCCACGTCCAATTT

Supplementary Table 2.

Number of RNA-seq reads used in mapping. These reads are paired-end sequences. The number of read pairs is half of the number of reads provided.

FW00K	Reads #	FW11K	Reads #	FW33K	Reads #	SW33K	Reads #
replicate1	112,184,342	replicate1	27,195,630	replicate1	26,848,226	replicate1	91,504,316
replicate2	95,444,412	replicate2	86,453,850	replicate2	93,673,728	replicate2	86,944,694
replicate3	69,184,542	replicate3	75,757,730	replicate3	71,730,008	replicate3	85,728,644

Supplementary Table 3.

Threespine stickleback kidney salt-responsive genes (*n* = 1,844). The 'A', 'B', 'C', and 'D' symbols indicate that the corresponding significance of expression difference was detected or not between FW00K-FW11K, FW00K-FW33K, FW11K-FW33K, and FW33K-SW33K, respectively. The '1' indicates the expression difference was significant and '0' not significant. The average expression level (in FPKM) is given for each sample.

Ensembl ID	Gene symbol	A	B	C	D	FW00K	FW11K	FW33K	SW33K
ENSGACG00000000015	*CAND2*	0	0	0	1	2.67	1.21	1.16	4.99
ENSGACG00000000022	*MATN4*	0	0	0	1	0.30	0.25	2.31	0.08
ENSGACG00000000038	*TIMM8B*	0	1	0	0	96.60	208.01	136.48	151.17
ENSGACG00000000041	-	0	0	0	1	1.90	1.37	1.52	0.40
ENSGACG00000000043	*FAM189A2*	0	0	0	1	6.95	4.73	1.99	8.70
ENSGACG00000000048	-	0	1	1	0	27.53	16.18	47.78	33.60
ENSGACG00000000063	-	0	1	0	0	13.67	6.70	2.37	2.23
ENSGACG00000000093	*TAC1*	0	1	1	0	2.68	1.24	9.98	19.37
ENSGACG00000000113	*PSMB8 (2 of 10)*	0	0	1	0	10.43	25.95	2.31	5.45
ENSGACG00000000118	*PSMB8 (7 of 10)*	0	0	1	0	7.97	18.66	1.69	3.96
ENSGACG00000000121	*LGALS1 (1 of 3)*	0	1	0	0	219.06	289.86	521.93	386.69
ENSGACG00000000125	*TAPBP (6 of 7)*	0	0	0	1	8.29	12.78	2.63	7.34
ENSGACG00000000134	*TAPBP (3 of 7)*	0	0	1	1	4.85	12.16	1.95	5.78
ENSGACG00000000155	-	0	0	0	1	2.50	1.15	2.63	0.26
ENSGACG00000000160	*LGALSL*	0	0	0	1	2.08	1.24	5.73	0.31
ENSGACG00000000220	-	0	1	1	1	0.05	0.00	3.18	0.07
ENSGACG00000000224	*SSR1 (5 of 12)*	0	1	0	0	11.94	20.81	36.63	34.86
ENSGACG00000000228	*MAN2B1*	0	1	0	0	35.27	28.91	81.16	29.45
ENSGACG00000000248	-	0	0	0	1	11.31	10.22	9.58	3.77
ENSGACG00000000270	*PTPN7*	0	0	1	1	28.05	58.93	8.56	4.37
ENSGACG00000000271	*ENOX1*	0	1	0	0	3.77	12.35	359.03	18.65
ENSGACG00000000277	*SSR1 (3 of 12)*	0	1	0	0	7.24	13.72	25.16	26.57
ENSGACG00000000280	*PLXNA3*	0	1	0	0	2.40	0.52	0.24	1.18
ENSGACG00000000286	*SLC35A2*	0	1	0	0	26.31	26.40	44.07	33.13
ENSGACG00000000289	*TSPEAR (1 of 2)*	0	1	0	0	2.59	2.29	4.22	8.20
ENSGACG00000000291	*UBE2G2*	0	0	1	0	71.03	52.58	121.96	128.80
ENSGACG00000000303	-	0	1	0	0	41.85	133.20	70.72	41.37
ENSGACG00000000312	*GLT8D1*	0	1	1	0	7.24	10.88	26.55	15.94
ENSGACG00000000313	*SPCS1*	0	1	0	0	45.52	113.31	121.89	110.72
ENSGACG00000000340	*SORT1 (2 of 2)*	0	1	1	1	7.24	5.08	28.38	5.80
ENSGACG00000000367	*FOXP4*	0	1	0	0	5.54	2.32	0.69	1.07
ENSGACG00000000389	-	0	0	1	0	12.66	15.50	1.46	0.69
ENSGACG00000000398	*CNNM3 (5 of 5)*	0	1	0	0	37.00	35.56	71.52	60.28
ENSGACG00000000425	-	0	1	0	0	44.51	90.75	391.48	351.29
ENSGACG00000000439	-	0	1	0	0	11.30	13.16	32.95	36.23
ENSGACG00000000467	*CNPY2*	0	1	0	0	4.78	9.65	12.29	9.59
ENSGACG00000000486	*ANKRD52 (2 of 2)*	0	1	0	0	12.32	12.77	22.74	16.32
ENSGACG00000000491	*YIPF2*	0	1	0	1	25.64	27.66	109.09	49.64
ENSGACG00000000505	*EDEM2*	0	1	0	0	8.55	10.29	12.86	19.33
ENSGACG00000000511	*PI16*	0	1	1	0	2.93	1.56	176.19	30.36
ENSGACG00000000521	*HM13*	0	1	0	0	44.87	59.21	145.52	173.58
ENSGACG00000000523	*GPR25*	0	0	0	1	0.33	0.89	0.12	1.45
ENSGACG00000000531	*SSR1 (10 of 12)*	0	1	0	0	5.34	8.64	13.13	13.73
ENSGACG00000000535	*SSR1 (1 of 12)*	0	1	0	0	7.37	13.84	25.27	27.37
ENSGACG00000000562	-	0	0	0	1	40.90	100.96	15.81	2.11
ENSGACG00000000619	*EPS8*	0	0	1	0	30.01	13.33	29.27	29.47
ENSGACG00000000631	*ATP8B1*	0	0	1	0	8.84	2.69	5.92	7.32
ENSGACG00000000635	*SOCS2*	0	1	0	0	8.51	4.54	0.92	1.13
ENSGACG00000000652	*TSPAN12*	0	1	0	0	2.07	0.62	0.04	0.37
ENSGACG00000000684	*LAMTOR4*	0	1	0	0	149.17	300.52	323.37	221.38
ENSGACG00000000691	*TYMP*	0	1	0	0	3.12	0.58	0.09	0.43
ENSGACG00000000704	-	0	1	0	0	2.39	0.50	0.07	0.11
ENSGACG00000000707	*STAT2*	1	0	0	0	26.72	81.18	27.25	18.29
ENSGACG00000000711	*OS9*	0	1	0	0	26.58	27.36	68.78	37.63
ENSGACG00000000721	*AP1S1*	0	1	0	0	22.98	19.97	28.95	37.35

ENSGACG00000000743	*FKBP11*	0	1	1	1	21.86	40.51	193.69	71.63
ENSGACG00000000746	*ARF3*	0	1	0	1	0.46	0.57	3.77	0.85
ENSGACG00000000781	-	0	0	0	1	0.15	0.06	0.00	0.54
ENSGACG00000000824	*UROC1*	0	1	0	1	249.01	106.65	3.38	82.49
ENSGACG00000000827	*PYGM (1 of 2)*	0	1	0	0	19.09	9.70	2.75	5.21
ENSGACG00000000830	*SEC61A1 (2 of 2)*	0	1	0	0	50.07	88.92	265.99	293.82
ENSGACG00000000843	*BOLA2*	1	0	0	0	52.37	215.10	61.95	54.36
ENSGACG00000000844	*SCARB1*	0	0	1	0	4.71	2.11	7.09	3.50
ENSGACG00000000845	-	0	1	0	0	1.77	5.56	46.36	52.56
ENSGACG00000000880	*RIOK3*	0	1	0	0	21.77	22.58	29.78	44.98
ENSGACG00000000897	*TEX264 (1 of 2)*	0	1	1	0	19.85	10.25	23.92	15.77
ENSGACG00000000902	*KIRREL (1 of 3)*	1	1	0	0	4.87	1.09	0.41	1.25
ENSGACG00000000923	*NPR1 (3 of 3)*	0	1	0	0	17.62	6.90	3.29	18.76
ENSGACG00000000996	*AP3B1 (1 of 2)*	0	1	0	0	19.68	21.46	23.12	24.53
ENSGACG00000001004	*EPS8L3 (1 of 2)*	0	1	0	0	73.40	28.29	10.04	13.19
ENSGACG00000001061	*NUAK2*	0	0	1	0	5.41	2.35	6.52	9.33
ENSGACG00000001065	*CELSR2*	0	1	0	0	6.42	1.86	1.23	3.69
ENSGACG00000001132	-	1	0	0	0	15.74	2.91	5.25	11.05
ENSGACG00000001143	*CROCC (1 of 2)*	0	1	0	0	7.32	3.77	0.91	1.69
ENSGACG00000001179	*PRKAR2A (2 of 2)*	0	1	0	0	12.91	5.19	1.05	1.99
ENSGACG00000001210	-	0	0	1	0	30.67	18.72	74.31	52.73
ENSGACG00000001238	*PDIA5*	0	1	0	0	20.99	34.69	36.26	78.29
ENSGACG00000001242	*ASZ1 (1 of 2)*	0	1	1	0	4.14	5.62	13.84	7.52
ENSGACG00000001260	-	0	0	0	1	1.89	1.08	0.20	1.66
ENSGACG00000001261	-	0	1	0	0	5.01	2.11	0.84	1.75
ENSGACG00000001275	*CISH*	0	1	0	0	15.17	3.34	1.15	0.89
ENSGACG00000001281	*ANAPC1*	0	1	0	0	10.47	4.91	1.54	4.53
ENSGACG00000001285	-	0	1	1	0	44.71	70.79	6.72	4.09
ENSGACG00000001287	*SPTBN5*	0	1	0	0	38.17	11.00	7.82	15.72
ENSGACG00000001293	*PSMB8 (5 of 10)*	0	0	1	0	15.62	46.22	3.70	8.44
ENSGACG00000001305	*SSR2*	0	1	0	0	125.86	236.05	632.08	640.48
ENSGACG00000001319	-	0	0	1	0	80.71	25.69	0.41	9.19
ENSGACG00000001321	-	0	0	1	0	123.50	27.55	0.73	0.00
ENSGACG00000001332	-	0	1	0	0	42.69	21.56	2.36	10.88
ENSGACG00000001379	*MRPS18B*	0	1	0	0	56.97	99.26	71.77	65.68
ENSGACG00000001382	*CADPS (2 of 2)*	0	1	0	0	2.61	0.30	0.06	0.60
ENSGACG00000001388	*ABCF1*	0	1	0	0	24.68	38.25	33.69	33.43
ENSGACG00000001404	*CNNM3 (3 of 5)*	0	1	0	0	12.59	10.12	27.57	21.33
ENSGACG00000001431	-	0	1	0	0	33.11	65.18	89.70	52.56
ENSGACG00000001432	-	0	0	0	1	3.58	1.17	0.27	3.18
ENSGACG00000001459	-	0	0	1	0	1.65	0.05	2.02	0.14
ENSGACG00000001461	*PPP1R11*	0	1	0	0	10.82	13.99	14.35	12.71
ENSGACG00000001466	*FKBP5*	0	1	0	0	38.72	131.72	224.56	144.59
ENSGACG00000001474	*SAR1B*	0	1	0	0	58.67	95.37	99.05	110.91
ENSGACG00000001482	-	1	1	0	0	0.03	1.40	1.12	1.19
ENSGACG00000001487	*KLF11 (2 of 2)*	0	0	0	1	2.94	4.12	0.74	11.24
ENSGACG00000001502	*LGALS8 (2 of 2)*	0	1	0	0	50.12	77.58	59.71	49.51
ENSGACG00000001509	*MARCO*	0	1	0	0	47.80	106.57	7.74	14.08
ENSGACG00000001531	-	1	0	0	0	2.45	15.63	2.91	0.75
ENSGACG00000001534	*ADHFE1*	0	1	0	0	63.80	89.88	122.26	245.94
ENSGACG00000001553	*HYAL2 (2 of 2)*	0	1	1	0	7.37	9.17	48.32	32.58
ENSGACG00000001576	*MANF*	0	1	1	0	29.40	39.42	187.47	205.54
ENSGACG00000001584	*ITGB1 (1 of 2)*	0	1	0	0	23.05	27.34	26.42	35.85
ENSGACG00000001611	*FAM132B*	1	0	0	0	2.54	16.24	2.14	1.60
ENSGACG00000001638	*CYP2J2 (6 of 6)*	0	1	0	0	103.23	31.84	14.09	14.07
ENSGACG00000001640	*CYP2J2 (5 of 6)*	0	1	0	0	114.83	36.22	17.46	18.05
ENSGACG00000001644	*TMCC1 (2 of 2)*	0	1	0	0	10.04	17.43	39.06	47.24
ENSGACG00000001674	*NEBL*	0	1	0	0	6.00	1.81	0.30	1.35
ENSGACG00000001677	*AKR7A3*	0	1	0	0	33.81	47.06	77.07	77.26
ENSGACG00000001678	-	0	1	0	0	8.28	2.70	0.38	2.16
ENSGACG00000001736	-	0	0	0	1	3.06	1.63	3.15	0.25
ENSGACG00000001742	-	0	0	1	1	3.06	1.37	3.06	0.37
ENSGACG00000001744	*CNNM3 (4 of 5)*	0	1	0	0	12.44	9.54	28.06	21.69
ENSGACG00000001745	-	0	1	0	0	7.50	1.79	0.72	0.93
ENSGACG00000001748	-	0	0	0	1	2.99	1.36	3.13	0.37
ENSGACG00000001759	*SSR1 (4 of 12)*	0	1	0	0	6.88	12.38	20.52	19.05

ENSGACG00000001764	*SSR1 (2 of 12)*	0	1	0	0	5.83	10.38	17.81	16.68
ENSGACG00000001766	*SSR1 (9 of 12)*	0	1	0	0	3.92	7.51	14.42	18.81
ENSGACG00000001775	*SSR1 (7 of 12)*	0	1	0	0	7.67	12.69	19.98	20.22
ENSGACG00000001804	-	0	0	0	1	28.46	35.36	17.64	69.45
ENSGACG00000001814	*IDI2*	0	1	0	0	49.05	49.12	103.14	43.51
ENSGACG00000001833	*SSR1 (11 of 12)*	0	1	0	0	12.34	20.66	36.63	36.19
ENSGACG00000001834	*PSMB8 (10 of 10)*	0	0	1	0	13.36	37.40	3.09	7.30
ENSGACG00000001852	*CPNE3 (2 of 4)*	0	1	1	0	7.06	7.05	19.10	7.25
ENSGACG00000001854	*CPNE3 (3 of 4)*	0	1	1	1	7.04	6.05	16.62	6.21
ENSGACG00000001856	*HSD17B8*	0	1	0	0	30.80	55.83	60.72	52.11
ENSGACG00000001867	-	0	1	0	0	6.67	15.62	21.88	19.82
ENSGACG00000001873	*COMMD6*	0	1	0	1	26.39	35.31	47.10	20.12
ENSGACG00000001877	*CNNM3 (2 of 5)*	0	1	0	0	14.62	13.91	32.49	23.62
ENSGACG00000001893	*GPR183*	0	0	1	0	18.33	28.85	4.45	4.09
ENSGACG00000001911	*SLC13A5 (2 of 2)*	1	1	0	0	33.07	7.34	5.83	9.11
ENSGACG00000001936	*RNASEK (1 of 2)*	0	0	0	1	95.89	128.33	105.27	40.20
ENSGACG00000001947	*PSMB8 (4 of 10)*	0	0	1	0	12.38	30.26	2.50	5.55
ENSGACG00000001963	*ENPEP*	0	1	0	0	24.80	10.19	53.79	91.52
ENSGACG00000001971	-	0	1	0	0	0.28	0.64	1.09	0.81
ENSGACG00000001973	-	0	1	0	0	101.76	64.79	9.91	30.22
ENSGACG00000002010	*AK2*	0	1	0	0	85.11	142.53	118.87	170.49
ENSGACG00000002019	*KLHL25*	0	0	0	1	8.74	5.46	2.70	10.64
ENSGACG00000002037	*ATAD2 (2 of 2)*	0	1	0	0	6.42	2.53	0.69	0.69
ENSGACG00000002066	-	0	1	0	0	0.16	0.60	1.35	0.46
ENSGACG00000002087	-	0	0	1	0	1.54	0.34	335.45	8.94
ENSGACG00000002116	*NSMAF*	0	1	0	0	14.02	13.97	27.61	35.66
ENSGACG00000002151	*TMEM231*	0	1	0	0	17.49	14.85	29.27	28.33
ENSGACG00000002163	-	0	0	0	1	32.47	74.44	12.24	2.56
ENSGACG00000002180	*THTPA*	0	1	0	0	3.52	8.45	13.26	20.33
ENSGACG00000002191	*RAMP1 (1 of 2)*	0	1	0	0	11.42	3.26	0.78	2.48
ENSGACG00000002246	*TTC27*	0	0	0	1	9.75	10.68	4.24	19.99
ENSGACG00000002276	*IPO4*	0	1	0	0	9.22	18.21	23.67	30.64
ENSGACG00000002280	-	0	0	1	0	19.51	38.83	4.84	5.14
ENSGACG00000002286	*PDCD6*	0	0	0	1	41.70	63.96	33.60	17.87
ENSGACG00000002309	*TXNDC5*	0	1	0	0	67.56	115.00	260.12	435.15
ENSGACG00000002319	*THRAP3 (2 of 2)*	0	1	0	0	50.11	55.14	121.36	167.32
ENSGACG00000002324	*EEF1E1*	0	1	0	0	12.18	29.26	27.41	48.05
ENSGACG00000002346	*ESYT3 (3 of 3)*	0	1	1	0	2.48	1.84	8.22	6.06
ENSGACG00000002348	*ESYT3 (1 of 3)*	0	1	1	0	3.16	2.24	9.04	6.31
ENSGACG00000002350	*ESYT3 (2 of 3)*	0	1	0	0	2.47	1.35	5.70	4.60
ENSGACG00000002357	*MYO1G*	0	1	1	0	16.52	19.15	2.94	2.71
ENSGACG00000002358	*TM9SF2*	0	1	0	0	23.15	22.49	28.95	29.37
ENSGACG00000002362	*PHLDB2 (1 of 2)*	0	1	0	0	11.76	3.65	1.97	4.89
ENSGACG00000002364	-	0	1	0	0	36.49	10.67	5.60	5.41
ENSGACG00000002368	*CHKA*	0	1	0	0	25.81	5.80	3.07	6.32
ENSGACG00000002378	-	0	1	0	0	42.48	13.06	5.38	12.12
ENSGACG00000002394	*DAP*	0	1	0	0	49.18	64.92	313.28	413.18
ENSGACG00000002401	*GULP1*	0	1	0	1	19.97	28.37	45.04	21.20
ENSGACG00000002405	-	0	1	0	0	32.13	9.99	6.84	10.50
ENSGACG00000002416	-	0	0	0	1	10.67	14.92	10.11	3.52
ENSGACG00000002419	*SLC12A3*	0	1	0	0	116.96	83.31	24.11	77.31
ENSGACG00000002433	*APOE (2 of 2)*	0	1	1	1	0.35	0.81	8.42	1.22
ENSGACG00000002472	*RPP40*	0	1	0	0	12.29	24.42	16.62	20.54
ENSGACG00000002475	-	0	0	0	1	164.28	155.53	86.69	19.60
ENSGACG00000002492	-	0	1	1	0	10.88	8.21	39.13	24.00
ENSGACG00000002497	*FAM65A*	0	1	1	0	12.91	5.02	38.26	36.83
ENSGACG00000002499	-	0	1	1	0	14.95	13.92	59.67	31.29
ENSGACG00000002508	*IGFBP5 (1 of 2)*	1	1	0	0	81.11	13.02	5.52	24.35
ENSGACG00000002512	*PDIA4*	0	1	1	0	44.03	55.67	550.90	546.02
ENSGACG00000002514	*CYP27A1*	0	1	0	0	52.08	18.20	7.52	13.88
ENSGACG00000002515	*RIC8A*	0	1	0	0	14.00	4.17	1.47	6.25
ENSGACG00000002518	*CDK1*	0	0	1	0	27.39	84.50	9.79	5.29
ENSGACG00000002519	*GPT (1 of 2)*	0	1	0	0	31.89	9.57	3.12	20.06
ENSGACG00000002530	*CTU2*	0	1	0	0	10.25	14.45	16.32	24.74
ENSGACG00000002548	-	0	0	0	1	21.69	7.34	0.98	15.05
ENSGACG00000002559	*HGD*	0	1	0	0	2.31	0.26	0.04	0.80

ENSGACG00000002588	-	0	1	0	0	5.81	10.65	47.17	38.16
ENSGACG00000002589	*MCFD2*	0	1	0	0	12.99	30.83	102.74	79.70
ENSGACG00000002603	*PDK3*	0	1	0	1	21.52	21.82	64.00	6.54
ENSGACG00000002614	*BCCIP*	0	1	0	0	39.52	77.17	67.47	101.42
ENSGACG00000002624	*SLC17A9*	0	1	0	0	3.71	4.92	10.27	9.73
ENSGACG00000002660	*KIAA0319L*	0	1	1	0	10.81	7.46	27.20	26.06
ENSGACG00000002666	*HAAO*	0	1	0	0	38.06	31.24	4.26	30.55
ENSGACG00000002667	*AARS*	0	1	0	0	14.52	23.24	20.51	22.30
ENSGACG00000002686	*FBXO15 (1 of 2)*	0	0	0	1	12.38	9.16	7.31	3.51
ENSGACG00000002693	*CBLN2*	1	0	0	0	0.70	8.19	1.18	4.20
ENSGACG00000002694	*CDCA8*	0	1	1	0	24.98	33.38	4.20	5.80
ENSGACG00000002700	*RTTN*	0	1	0	0	7.57	4.63	0.96	1.29
ENSGACG00000002715	*SLC40A1*	0	1	0	0	15.62	6.11	2.43	2.56
ENSGACG00000002749	-	0	1	0	0	6.64	2.96	1.32	2.52
ENSGACG00000002750	*ABTB1*	0	1	0	0	26.07	21.46	33.85	24.28
ENSGACG00000002765	*RDH10*	0	1	0	0	142.79	34.98	18.97	14.45
ENSGACG00000002818	*TRAM1L1*	0	1	0	0	18.21	33.46	96.68	115.75
ENSGACG00000002828	-	0	1	0	1	0.29	2.16	2.08	0.09
ENSGACG00000002833	-	0	1	0	0	20.96	11.64	36.56	21.35
ENSGACG00000002837	*SULF1*	0	1	0	0	9.72	3.63	2.05	1.60
ENSGACG00000002845	*PREX2*	0	0	0	1	3.63	1.85	1.55	8.58
ENSGACG00000002869	*MCF2L (1 of 2)*	0	0	1	0	4.41	1.73	5.97	3.09
ENSGACG00000002873	*PPHLN1 (4 of 4)*	0	1	0	0	11.75	15.92	17.23	17.79
ENSGACG00000002882	-	1	0	0	0	7.18	1.37	2.45	5.73
ENSGACG00000002891	*ARHGEF7 (1 of 2)*	0	0	0	1	3.13	1.41	3.23	1.25
ENSGACG00000002902	-	0	1	0	0	10.68	0.42	0.05	0.07
ENSGACG00000002905	*BLNK*	0	0	1	0	17.37	25.26	4.42	2.60
ENSGACG00000002922	*SGK3*	0	0	0	1	28.39	29.11	28.12	10.63
ENSGACG00000002925	*EEF2K*	0	1	0	0	34.98	27.06	108.74	79.17
ENSGACG00000002945	-	0	1	0	0	107.36	54.96	10.70	72.72
ENSGACG00000002955	-	0	1	0	0	59.84	3.20	0.06	0.13
ENSGACG00000002958	*KIAA0319*	0	0	0	1	3.72	2.42	0.82	11.88
ENSGACG00000002962	*KMO*	0	1	0	0	3.92	2.27	0.16	0.90
ENSGACG00000002988	*CDC42EP3*	0	1	0	0	21.08	8.74	3.39	5.84
ENSGACG00000002992	*POU3F3 (2 of 2)*	0	1	0	0	22.49	25.67	36.44	35.84
ENSGACG00000003007	*CYP1B1*	0	1	0	0	0.51	0.72	1.94	0.58
ENSGACG00000003014	*MED30*	0	1	0	0	18.00	35.59	27.54	33.02
ENSGACG00000003038	*UXS1*	0	1	0	0	9.05	9.26	16.81	29.26
ENSGACG00000003039	*TRIM35 (20 of 28)*	0	0	0	1	1.01	1.55	0.12	1.67
ENSGACG00000003040	*C1QTNF9B*	0	1	0	0	91.20	51.16	5.91	51.45
ENSGACG00000003046	-	0	0	0	1	1.41	2.87	0.75	3.97
ENSGACG00000003047	*SPATA13*	0	1	0	0	34.32	13.29	7.07	20.34
ENSGACG00000003048	*SDC2*	0	1	0	0	24.82	7.90	3.33	7.08
ENSGACG00000003049	*SFXN2*	0	1	0	0	13.47	21.87	42.98	36.19
ENSGACG00000003051	*TRIM35 (22 of 28)*	0	0	0	1	1.63	2.42	0.19	2.86
ENSGACG00000003052	*CALR3 (1 of 2)*	0	1	1	0	127.71	166.85	1011.44	567.96
ENSGACG00000003061	*TPMT*	0	1	0	0	15.92	25.13	20.21	29.03
ENSGACG00000003063	-	0	1	1	0	2.06	0.98	20.82	8.59
ENSGACG00000003069	-	0	1	1	0	167.13	63.62	8.13	24.30
ENSGACG00000003084	*TMEM79 (1 of 2)*	0	1	0	0	22.34	16.23	32.35	28.17
ENSGACG00000003087	*TM9SF3*	0	1	0	0	34.17	34.36	47.61	53.05
ENSGACG00000003090	-	0	1	0	1	27.13	22.09	4.01	25.16
ENSGACG00000003133	*PRDX3*	0	1	0	0	61.50	129.53	103.87	134.78
ENSGACG00000003149	*LRRC59*	0	1	0	0	6.74	9.35	17.39	23.80
ENSGACG00000003191	*EIF3A*	0	1	0	0	66.08	77.99	91.78	103.23
ENSGACG00000003210	-	0	1	1	0	14.46	19.82	91.80	69.95
ENSGACG00000003217	-	0	1	0	0	9.54	7.15	11.13	15.64
ENSGACG00000003248	*PLA2G15*	0	1	0	0	34.20	35.61	50.87	75.45
ENSGACG00000003249	*SCUBE3*	0	1	0	0	4.15	2.03	0.56	0.81
ENSGACG00000003254	*KIAA1407*	0	1	0	0	7.36	7.78	22.80	25.30
ENSGACG00000003273	-	0	1	0	0	6.31	2.39	0.90	1.03
ENSGACG00000003287	*C1orf51*	0	1	0	1	16.32	6.07	1.79	7.92
ENSGACG00000003292	-	0	1	0	0	11.81	10.71	1.29	7.25
ENSGACG00000003299	*WT1 (2 of 2)*	0	1	0	0	20.35	5.28	2.02	4.88
ENSGACG00000003309	*NOXA1*	0	1	0	0	8.76	5.13	0.82	2.86
ENSGACG00000003312	*TCP11L1*	1	1	0	0	8.99	1.46	0.97	1.92

ENSGACG00000003314	*RPGR (2 of 2)*	1	0	0	0	0.25	0.00	0.00	0.02
ENSGACG00000003361	*COL1A1 (2 of 2)*	1	1	0	0	14.46	65.39	36.76	18.08
ENSGACG00000003374	-	0	0	0	1	4.02	8.02	1.74	9.76
ENSGACG00000003397	-	0	0	0	1	10.22	9.67	1.31	6.76
ENSGACG00000003423	*ALDH18A1 (1 of 2)*	0	1	0	0	19.53	26.54	33.42	76.33
ENSGACG00000003440	*TPD52*	0	1	0	0	47.19	33.66	65.38	65.42
ENSGACG00000003443	*FGFR2*	0	1	0	0	43.95	9.71	7.13	12.30
ENSGACG00000003457	-	0	1	0	0	113.22	112.79	274.21	447.85
ENSGACG00000003461	-	1	0	0	0	914.45	45.95	16.98	203.30
ENSGACG00000003473	-	1	1	0	0	118.93	10.33	8.92	19.55
ENSGACG00000003477	*GMPR*	1	0	0	0	1.84	15.68	1.96	0.99
ENSGACG00000003482	-	1	1	0	0	4.79	0.77	0.59	0.98
ENSGACG00000003483	-	0	0	0	1	33.30	4.74	3.09	18.14
ENSGACG00000003487	*SYT12*	1	1	0	0	33.72	6.79	2.73	5.42
ENSGACG00000003499	*POP1*	0	0	0	1	3.21	2.56	0.84	3.80
ENSGACG00000003501	-	1	1	0	0	5.68	0.46	0.24	2.13
ENSGACG00000003511	*PDZD7*	0	1	0	0	11.21	2.71	1.14	2.90
ENSGACG00000003512	*CRB2 (2 of 2)*	0	1	0	0	1.73	0.43	0.17	0.55
ENSGACG00000003537	*MEP1B (2 of 2)*	0	1	0	0	6.86	17.03	36.06	42.98
ENSGACG00000003560	*NR6A1*	0	1	0	0	4.06	0.80	0.39	1.57
ENSGACG00000003573	*ANKRD30B*	1	1	0	0	14.03	2.73	2.12	2.70
ENSGACG00000003591	*MTDH (1 of 2)*	0	1	0	0	20.43	28.02	39.52	56.29
ENSGACG00000003597	*PPM1H (1 of 2)*	0	1	0	0	9.41	2.00	0.91	1.48
ENSGACG00000003635	*ANK3 (2 of 2)*	0	1	0	0	5.64	4.61	10.51	16.62
ENSGACG00000003651	-	0	1	0	0	17.84	16.81	37.34	54.08
ENSGACG00000003652	*G6PC (1 of 2)*	0	1	0	0	54.37	7.47	2.11	11.40
ENSGACG00000003670	*CHMP4B (1 of 2)*	0	1	0	0	34.76	29.01	44.33	49.07
ENSGACG00000003682	-	0	1	0	0	21.58	22.06	3.35	5.64
ENSGACG00000003691	-	0	1	0	1	69.64	69.42	10.35	36.79
ENSGACG00000003693	*PLOD2*	0	1	0	0	9.96	5.64	1.62	12.76
ENSGACG00000003722	*MEOX1*	1	1	0	0	12.21	0.89	0.58	2.51
ENSGACG00000003734	*DHFR*	0	1	0	0	30.79	82.49	70.98	87.41
ENSGACG00000003761	*TEAD4*	0	0	0	1	0.84	0.37	2.84	0.13
ENSGACG00000003787	-	0	0	0	1	2.35	1.61	0.80	5.67
ENSGACG00000003789	-	0	1	1	1	0.27	0.55	3.53	0.24
ENSGACG00000003791	*FAM198A*	0	1	1	1	0.34	0.37	2.47	0.14
ENSGACG00000003802	*SDF2L1*	0	1	0	0	21.88	42.77	77.42	50.73
ENSGACG00000003805	*ANO10*	0	1	0	0	6.58	7.01	11.85	12.90
ENSGACG00000003816	*UBA5*	0	1	0	0	18.77	28.02	50.90	62.62
ENSGACG00000003818	-	0	0	0	1	17.63	21.35	5.02	1.79
ENSGACG00000003828	-	0	1	0	0	3.30	4.33	4.83	2.35
ENSGACG00000003853	*ABCA2*	0	1	1	0	0.44	0.63	10.79	5.07
ENSGACG00000003854	*HDAC10*	0	1	0	0	14.75	14.81	28.99	52.28
ENSGACG00000003864	*RAB1B*	0	1	0	0	52.82	64.86	86.57	99.11
ENSGACG00000003874	*PARVB*	0	1	0	0	27.32	32.28	4.11	3.31
ENSGACG00000003875	*CDK5RAP3*	0	1	0	0	18.34	37.21	92.97	48.84
ENSGACG00000003899	*PARVG*	0	1	0	0	37.01	26.13	5.72	4.02
ENSGACG00000003901	*TMCO1*	0	1	0	0	62.26	122.74	141.04	196.67
ENSGACG00000003911	*PLXNB2 (1 of 3)*	1	1	0	0	13.24	2.03	2.25	2.72
ENSGACG00000003935	*ABHD6 (2 of 2)*	0	1	0	0	25.60	5.59	2.00	2.96
ENSGACG00000003947	*APPL2*	0	0	1	0	11.51	4.64	12.30	10.13
ENSGACG00000003951	*TMEM254*	0	1	0	0	37.71	49.01	49.34	53.99
ENSGACG00000003957	*NUAK1*	0	1	0	0	5.84	1.08	0.36	0.91
ENSGACG00000003971	-	0	1	1	1	0.02	0.03	0.99	0.01
ENSGACG00000003974	*B3GAT2*	0	1	0	0	3.79	0.65	0.21	0.77
ENSGACG00000003981	*CLPTM1L (1 of 2)*	0	1	0	0	11.00	9.85	14.56	17.56
ENSGACG00000004047	*STX4*	0	0	0	1	33.22	53.13	15.42	47.76
ENSGACG00000004049	-	0	1	0	0	124.99	74.78	19.11	36.69
ENSGACG00000004061	-	0	1	0	0	0.74	2.67	2.09	4.25
ENSGACG00000004063	*ADAMTS20*	1	1	0	0	6.58	0.84	0.27	1.53
ENSGACG00000004068	*GADD45A (1 of 2)*	0	1	0	0	27.75	33.66	67.54	26.49
ENSGACG00000004076	*SLC39A4*	0	1	1	0	0.28	0.50	3.65	5.65
ENSGACG00000004095	*NDEL1 (1 of 2)*	0	1	0	0	5.44	6.66	11.64	12.71
ENSGACG00000004110	*SRP9*	0	1	0	0	107.16	223.14	428.45	395.50
ENSGACG00000004126	-	0	1	0	0	62.01	27.74	89.68	179.60
ENSGACG00000004127	*GSS*	0	1	0	0	6.38	7.98	12.78	10.65

ENSGACG00000004143	*AKAP9*	0	1	0	0	6.84	4.09	8.27	8.73
ENSGACG00000004153	-	0	1	0	0	174.56	112.47	286.25	399.30
ENSGACG00000004159	-	0	1	0	0	3.35	4.35	12.82	6.98
ENSGACG00000004166	*DNAJC3*	0	1	1	0	23.09	28.60	170.87	226.52
ENSGACG00000004174	*EGLN1*	0	1	0	1	13.17	14.57	17.98	8.83
ENSGACG00000004185	*SLC5A6 (1 of 2)*	0	0	0	1	18.47	9.25	7.88	52.67
ENSGACG00000004189	*UGGT2*	0	1	0	0	7.02	8.80	7.96	12.65
ENSGACG00000004192	*MRPL9*	0	1	0	0	27.06	32.73	32.79	54.76
ENSGACG00000004218	-	0	1	0	0	9.97	1.81	0.86	3.52
ENSGACG00000004242	-	0	1	1	1	0.01	0.45	62.12	0.94
ENSGACG00000004245	*DPM1*	0	1	0	0	20.13	35.01	63.64	48.36
ENSGACG00000004260	*ARFGEF2*	0	1	0	0	9.50	8.28	15.51	19.33
ENSGACG00000004266	*TNFRSF21*	0	1	1	0	2.22	1.26	4.92	16.61
ENSGACG00000004277	-	1	1	0	0	8.59	1.28	0.60	0.89
ENSGACG00000004294	-	0	1	0	0	13.27	12.34	17.09	22.50
ENSGACG00000004315	-	0	1	1	0	12.59	20.34	71.39	18.73
ENSGACG00000004318	-	0	1	0	0	0.44	0.46	1.60	0.85
ENSGACG00000004321	*NDUFS1 (1 of 2)*	0	0	1	0	14.97	4.90	26.60	83.98
ENSGACG00000004333	*GOLT1B*	0	1	0	0	16.15	21.73	41.05	40.68
ENSGACG00000004345	*EIF2AK2 (2 of 2)*	0	1	0	1	9.00	25.45	20.20	6.66
ENSGACG00000004351	*TECPR2*	0	1	1	1	17.74	10.70	211.73	31.01
ENSGACG00000004357	*CHCHD3 (2 of 2)*	0	1	0	0	17.50	6.61	2.52	3.14
ENSGACG00000004362	*GRB7*	0	0	0	1	3.92	1.05	0.71	4.02
ENSGACG00000004364	*RRN3*	0	1	0	0	18.78	19.19	42.81	40.00
ENSGACG00000004370	*HEATR5B*	0	1	0	0	26.68	30.43	40.61	36.96
ENSGACG00000004371	*CINP*	0	1	0	0	7.84	18.47	11.81	25.44
ENSGACG00000004374	*ARHGEF28*	0	1	0	0	7.21	3.78	8.88	7.43
ENSGACG00000004375	-	0	1	0	0	9.17	11.82	22.39	34.47
ENSGACG00000004376	-	0	1	0	0	16.43	24.98	39.20	56.15
ENSGACG00000004396	-	0	0	0	1	3.72	1.79	1.76	0.32
ENSGACG00000004416	*EIF2AK3*	0	1	1	0	5.44	5.02	15.28	12.08
ENSGACG00000004427	*ELP3*	0	1	0	0	16.24	20.08	22.24	26.01
ENSGACG00000004440	*TTC39C (1 of 2)*	0	0	0	1	0.76	0.54	0.21	1.47
ENSGACG00000004444	*AGPS*	0	1	0	0	15.17	21.29	16.91	23.25
ENSGACG00000004451	*SLCO4A1*	0	1	1	1	7.85	6.83	25.75	11.95
ENSGACG00000004454	*DENND2C*	0	1	1	0	10.60	8.91	39.38	19.60
ENSGACG00000004460	*FAM49B*	0	1	0	0	87.21	90.34	17.58	19.89
ENSGACG00000004476	-	0	1	1	0	24.91	8.57	1.15	5.06
ENSGACG00000004482	*STMN4 (1 of 2)*	1	1	0	0	3.25	19.34	9.35	8.77
ENSGACG00000004490	-	0	1	0	0	35.18	40.99	64.36	79.01
ENSGACG00000004493	*JAG1 (1 of 2)*	0	1	0	0	10.84	4.36	1.83	4.38
ENSGACG00000004498	-	0	1	0	0	8.44	7.82	15.98	10.53
ENSGACG00000004502	*DTYMK*	0	1	1	0	52.77	70.54	10.54	36.18
ENSGACG00000004506	-	0	1	0	0	18.54	24.26	44.95	55.76
ENSGACG00000004516	*DMC1*	0	0	1	0	0.11	1.45	0.06	0.06
ENSGACG00000004537	*DPM3*	0	1	0	0	53.63	145.92	217.55	131.46
ENSGACG00000004566	*MACROD2*	0	1	0	0	19.45	18.99	33.28	32.25
ENSGACG00000004567	*WIPI1 (2 of 2)*	0	0	0	1	12.78	6.80	9.71	4.58
ENSGACG00000004583	*FST*	0	1	0	0	5.57	0.78	0.31	0.73
ENSGACG00000004610	-	0	1	0	1	1.87	6.30	14.10	3.46
ENSGACG00000004619	*SPCS2*	0	1	0	0	131.43	192.63	321.09	209.42
ENSGACG00000004621	*AZIN1 (1 of 2)*	0	1	0	0	30.72	36.23	59.32	68.04
ENSGACG00000004623	*FARP2*	0	1	0	1	12.61	4.69	2.69	8.58
ENSGACG00000004651	*PFKFB2 (1 of 2)*	0	0	1	0	0.74	0.43	1.37	1.09
ENSGACG00000004664	*SLC4A2 (3 of 3)*	0	1	1	0	18.74	11.79	30.48	29.15
ENSGACG00000004670	*NEIL1*	0	1	0	0	20.21	4.98	2.45	4.25
ENSGACG00000004704	*FAM65B*	0	1	1	0	18.90	17.34	2.71	2.15
ENSGACG00000004729	*RCN3*	0	1	1	0	12.90	18.16	163.83	136.35
ENSGACG00000004737	*RRBP1*	0	1	0	0	12.24	12.82	33.02	41.55
ENSGACG00000004745	-	0	1	0	0	49.14	130.00	346.77	317.97
ENSGACG00000004748	*EIF2A*	0	1	0	0	28.26	50.41	41.91	61.93
ENSGACG00000004790	-	0	1	0	0	7.17	9.39	0.90	0.63
ENSGACG00000004811	*MASP1*	0	1	0	0	3.61	3.73	5.30	8.61
ENSGACG00000004820	*TDRD6*	0	0	1	0	1.73	0.64	0.02	0.00
ENSGACG00000004833	-	0	1	0	0	18.12	12.89	25.43	46.35
ENSGACG00000004848	*IGFBP1 (1 of 2)*	1	1	0	0	32.82	2.50	0.72	1.29

ENSGACG00000004852	*PXDC1*	0	1	0	0	12.46	5.08	2.19	5.40
ENSGACG00000004875	*WDR74*	0	0	0	1	7.42	8.66	3.61	13.08
ENSGACG00000004892	*TINAGL1*	0	1	0	0	49.12	22.37	6.67	17.99
ENSGACG00000004923	*DSP (2 of 2)*	0	1	0	1	5.66	1.03	0.87	5.97
ENSGACG00000004933	-	0	0	0	1	6.47	6.28	10.41	3.49
ENSGACG00000004940	-	0	1	1	0	5.77	1.58	29.69	16.77
ENSGACG00000004942	-	0	0	0	1	0.53	0.95	1.31	0.05
ENSGACG00000004949	-	0	1	0	0	141.39	141.66	286.95	304.64
ENSGACG00000004960	*HAO1*	0	1	0	0	70.59	42.60	5.76	66.27
ENSGACG00000004961	*GET4*	0	1	0	0	22.81	22.95	34.91	40.43
ENSGACG00000004965	*RAB25 (2 of 2)*	0	0	1	0	33.96	8.37	22.95	44.90
ENSGACG00000004992	*C11orf75*	0	1	0	0	35.45	140.86	207.19	90.39
ENSGACG00000005002	*OTUD6A*	0	1	0	0	20.09	42.38	26.86	31.78
ENSGACG00000005008	-	0	0	1	0	20.67	10.12	24.79	30.16
ENSGACG00000005020	*GMDS*	0	1	0	0	15.37	15.30	25.94	24.49
ENSGACG00000005029	*ICT1*	0	1	0	0	23.33	27.82	76.88	70.10
ENSGACG00000005030	-	0	0	1	0	3.00	0.89	5.31	12.24
ENSGACG00000005034	*NAV1 (1 of 2)*	0	1	0	0	3.83	1.48	0.37	1.32
ENSGACG00000005036	-	0	0	0	1	6.72	5.28	0.96	4.44
ENSGACG00000005049	-	1	0	0	0	7.87	45.45	9.95	3.85
ENSGACG00000005081	*CPXM1*	0	1	0	0	16.25	10.35	3.45	5.74
ENSGACG00000005096	*METTL5*	0	1	0	0	14.31	36.34	18.45	20.28
ENSGACG00000005124	*ATP13A2*	0	1	1	0	11.78	10.54	50.38	25.04
ENSGACG00000005143	*COL1A1 (1 of 2)*	1	1	0	0	6.81	29.91	17.78	8.46
ENSGACG00000005172	-	0	1	0	0	25.41	40.49	63.17	113.60
ENSGACG00000005173	*HMBS*	1	0	0	0	10.35	76.14	15.72	15.02
ENSGACG00000005180	*PIK3R3 (1 of 2)*	0	1	0	0	12.43	2.93	1.63	5.93
ENSGACG00000005183	*ARCN1*	0	1	0	0	37.51	48.84	61.72	66.19
ENSGACG00000005186	*FAM46A (1 of 2)*	0	1	0	0	7.99	0.80	0.45	1.38
ENSGACG00000005213	*TLE3 (2 of 2)*	0	0	1	1	3.07	1.19	3.14	1.30
ENSGACG00000005221	*ARHGEF19*	0	1	0	0	19.67	8.51	3.74	12.50
ENSGACG00000005238	*GRINA (1 of 2)*	0	1	0	0	12.10	12.14	14.40	15.10
ENSGACG00000005260	*DDOST*	0	1	0	0	79.31	252.45	317.60	416.97
ENSGACG00000005261	*CYB561*	0	1	1	0	12.16	8.68	73.41	67.76
ENSGACG00000005270	*TANC2 (2 of 2)*	0	1	0	0	1.39	1.14	3.11	2.55
ENSGACG00000005280	-	0	0	0	1	0.58	0.16	0.15	1.44
ENSGACG00000005285	*ABCG4 (1 of 2)*	0	1	1	0	0.00	0.01	1.31	0.24
ENSGACG00000005320	*RHPN1*	0	0	0	1	6.73	5.42	4.44	15.61
ENSGACG00000005331	*CHID1*	0	1	1	0	25.64	22.32	80.94	63.12
ENSGACG00000005334	-	0	1	0	1	26.62	22.78	37.02	15.95
ENSGACG00000005348	*CAP2*	0	0	0	1	2.68	1.74	3.23	0.72
ENSGACG00000005370	*GOLGA4*	0	1	0	0	17.38	10.90	18.97	27.03
ENSGACG00000005385	*GALNT3*	0	1	1	1	0.60	0.59	2.21	0.28
ENSGACG00000005390	*TNK2 (2 of 2)*	0	1	1	0	0.54	0.17	45.06	6.85
ENSGACG00000005411	-	0	0	0	1	11.16	29.39	6.74	1.37
ENSGACG00000005414	*C11orf10*	0	1	0	0	486.69	1339.31	1538.17	1224.66
ENSGACG00000005415	-	0	0	0	1	12.26	36.63	7.84	0.71
ENSGACG00000005435	*MAP3K15*	0	0	0	1	4.04	3.02	1.92	0.54
ENSGACG00000005443	-	0	1	0	0	13.61	30.30	20.56	20.04
ENSGACG00000005456	*SLC39A11*	0	1	0	0	8.18	9.00	12.95	24.15
ENSGACG00000005483	-	0	1	0	0	18.39	4.26	1.93	2.87
ENSGACG00000005489	*SLC25A22*	1	1	0	0	20.60	3.08	2.09	3.37
ENSGACG00000005536	*EIF4E (2 of 2)*	0	1	1	0	25.39	45.97	109.74	129.13
ENSGACG00000005561	*ATHL1*	0	1	0	0	20.06	5.14	2.27	2.65
ENSGACG00000005567	*TSTA3 (2 of 2)*	0	1	0	0	20.28	31.71	77.23	54.04
ENSGACG00000005572	*RASD1 (1 of 2)*	0	1	0	0	83.68	57.36	311.29	275.21
ENSGACG00000005583	*RAB26 (2 of 2)*	0	1	1	0	11.19	10.84	209.04	54.34
ENSGACG00000005596	*ERLEC1*	0	1	1	0	35.94	51.26	413.73	483.43
ENSGACG00000005599	-	0	0	0	1	35.21	68.91	19.14	8.65
ENSGACG00000005603	*HAS2*	0	1	1	0	0.44	1.76	449.72	3.65
ENSGACG00000005604	*SOGA1*	0	0	0	1	1.59	1.15	0.66	2.63
ENSGACG00000005615	*DERL1*	0	1	0	0	19.86	27.43	37.72	41.51
ENSGACG00000005621	-	0	1	0	0	13.03	13.81	21.33	47.10
ENSGACG00000005625	*PPAPDC1B*	0	1	0	0	2.58	5.17	5.62	6.79
ENSGACG00000005632	*HPS5*	0	1	0	0	41.58	17.30	6.85	7.85
ENSGACG00000005642	*RAD54L2 (1 of 2)*	0	1	0	0	8.57	2.87	1.36	2.21

ENSGACG00000005649	-	0	1	0	0	30.52	11.66	6.55	9.59
ENSGACG00000005656	*TMEM115*	0	1	0	0	6.28	4.45	13.54	9.94
ENSGACG00000005657	*EEF1D (2 of 3)*	0	1	0	0	8.71	18.82	13.02	17.25
ENSGACG00000005661	*COPZ2*	0	1	0	0	19.45	34.09	204.07	163.34
ENSGACG00000005685	*ATAD2 (1 of 2)*	0	1	0	0	12.48	11.72	2.52	4.29
ENSGACG00000005698	*GNMT*	0	1	0	0	94.67	99.04	308.79	96.49
ENSGACG00000005703	*KCNIP3 (1 of 2)*	0	0	1	0	3.62	1.04	5.62	4.62
ENSGACG00000005708	*RGS5*	0	1	0	0	4.00	8.28	8.86	5.59
ENSGACG00000005711	-	0	1	0	0	20.86	22.50	67.78	16.35
ENSGACG00000005712	-	0	1	0	0	72.77	70.35	242.45	93.92
ENSGACG00000005720	*PRR15L (2 of 2)*	0	1	0	0	17.83	45.66	110.36	151.58
ENSGACG00000005750	*PTP4A2 (2 of 2)*	0	1	0	0	32.99	23.62	42.70	36.49
ENSGACG00000005757	*TANC1 (1 of 2)*	0	1	0	1	10.09	3.15	1.60	5.81
ENSGACG00000005769	*CELA1 (2 of 2)*	0	1	0	0	0.56	4.27	4.38	3.33
ENSGACG00000005800	*UPP2*	0	1	0	0	476.84	117.10	22.33	32.28
ENSGACG00000005810	*ACVR1*	0	1	0	0	21.59	6.48	3.89	13.21
ENSGACG00000005832	*KCNK1*	0	1	0	0	2.12	4.18	6.51	2.58
ENSGACG00000005836	-	0	1	0	0	33.15	49.13	91.07	46.77
ENSGACG00000005842	*PKP3 (1 of 2)*	0	1	0	0	30.79	7.15	4.65	9.34
ENSGACG00000005856	*LIN52*	1	0	1	0	28.00	116.74	15.87	23.28
ENSGACG00000005874	*POC1B-GALNT4*	0	1	1	0	9.37	5.30	16.42	14.09
ENSGACG00000005881	*B3GALT2 (1 of 2)*	0	0	0	1	5.54	6.68	2.98	21.42
ENSGACG00000005885	*FRMD1 (1 of 2)*	0	1	0	0	8.93	2.66	1.28	2.54
ENSGACG00000005889	*ANO5 (2 of 2)*	0	1	0	0	8.51	2.69	1.12	1.97
ENSGACG00000005894	-	0	1	0	1	1.48	3.97	9.39	0.58
ENSGACG00000005903	-	0	1	0	0	42.90	71.46	80.90	99.34
ENSGACG00000005908	*ASPM (3 of 3)*	0	0	1	0	3.39	5.52	0.84	0.37
ENSGACG00000005911	*ASPM (1 of 3)*	0	0	1	0	3.14	4.17	0.77	0.28
ENSGACG00000005928	*CTNNAL1*	0	1	1	0	14.25	11.60	24.74	31.56
ENSGACG00000005949	-	0	1	0	0	19.73	6.07	3.24	7.73
ENSGACG00000005950	*SPI1 (2 of 2)*	0	1	0	0	27.07	26.93	2.57	3.69
ENSGACG00000005951	*RGS6 (2 of 2)*	0	1	0	0	4.02	0.83	0.30	0.78
ENSGACG00000005955	*MFRP*	0	1	0	0	1.24	0.59	0.04	0.21
ENSGACG00000006001	*MYO5C*	0	1	1	0	6.77	2.71	13.89	9.87
ENSGACG00000006013	*PCSK7*	0	1	0	0	23.10	20.08	41.69	75.63
ENSGACG00000006029	-	1	1	0	0	23.01	2.07	0.28	0.19
ENSGACG00000006030	*TTC14*	0	0	1	0	40.97	23.65	96.27	103.27
ENSGACG00000006057	*EHHADH*	0	1	0	0	22.45	11.37	4.75	12.62
ENSGACG00000006129	*FRMD1 (2 of 2)*	1	1	0	0	14.47	2.50	0.86	4.05
ENSGACG00000006149	*CDO1*	0	1	0	0	16.73	52.29	383.79	134.33
ENSGACG00000006158	*DUOXA2*	0	0	1	0	1.17	0.38	1.62	0.70
ENSGACG00000006161	*FBXO32*	0	0	0	1	16.51	10.07	11.49	3.85
ENSGACG00000006163	*DUOX1*	0	0	1	0	6.88	2.02	8.91	3.22
ENSGACG00000006172	-	0	1	0	0	36.99	47.76	54.80	41.83
ENSGACG00000006174	*TP53I13 (1 of 2)*	0	1	0	0	1.66	2.53	7.95	11.57
ENSGACG00000006194	*INF2 (1 of 2)*	0	1	0	0	10.82	3.61	1.58	3.23
ENSGACG00000006198	*FAM84B*	0	0	0	1	15.52	13.08	7.87	2.28
ENSGACG00000006211	*ZNF385C (2 of 3)*	0	1	0	0	6.36	1.86	0.27	1.81
ENSGACG00000006219	*SLC27A5 (2 of 3)*	0	1	0	0	16.94	4.71	1.79	3.97
ENSGACG00000006227	*RAP1GAP2*	0	1	0	0	18.50	26.91	67.00	72.37
ENSGACG00000006229	*FKBP2*	0	1	0	0	138.72	239.21	377.67	426.90
ENSGACG00000006241	-	0	0	1	0	81.20	149.52	23.08	28.82
ENSGACG00000006254	*VWA5A (2 of 2)*	0	0	1	0	25.27	11.86	24.83	14.13
ENSGACG00000006265	*PTGER1 (2 of 2)*	0	1	0	0	3.61	4.98	18.44	16.24
ENSGACG00000006278	*FARSA*	0	1	0	0	41.17	59.75	80.42	97.44
ENSGACG00000006287	*UFL1*	0	1	0	0	16.73	16.09	20.43	28.48
ENSGACG00000006293	*EIF3H*	0	1	0	0	115.07	231.71	213.43	245.33
ENSGACG00000006298	*AKT1*	0	0	0	1	21.15	14.41	6.76	21.09
ENSGACG00000006309	*KIAA0284 (2 of 2)*	0	1	0	0	10.08	3.01	1.78	2.82
ENSGACG00000006317	*MXRA5 (2 of 2)*	0	0	0	1	0.64	0.28	0.04	1.25
ENSGACG00000006319	*FRK*	0	1	0	0	10.71	2.52	1.45	2.24
ENSGACG00000006323	*CDK6*	0	1	0	0	16.12	39.39	87.94	98.35
ENSGACG00000006337	*ANKIB1*	0	1	0	0	11.15	7.61	14.89	17.54
ENSGACG00000006338	*SRP14*	0	1	0	0	23.87	47.12	35.74	46.36
ENSGACG00000006344	*HSPA13*	0	1	0	0	4.16	5.12	7.90	8.66
ENSGACG00000006349	*IHH (2 of 2)*	0	1	0	0	4.85	3.81	0.55	2.09

ENSGACG00000006351	-	1	1	0	1	8.31	1.43	0.36	2.18
ENSGACG00000006370	*NPTN (1 of 2)*	1	0	0	0	1.94	0.10	0.13	0.34
ENSGACG00000006391	*BECN1*	0	1	0	0	21.67	25.80	33.10	26.68
ENSGACG00000006399	*FAM210B*	0	1	0	0	20.46	30.11	31.66	18.54
ENSGACG00000006408	*DNAJB2*	0	0	0	1	23.56	12.33	4.00	19.28
ENSGACG00000006426	-	0	1	0	0	11.65	12.38	15.98	16.76
ENSGACG00000006439	*ITGB8*	0	0	0	1	14.99	5.41	8.54	30.89
ENSGACG00000006444	*ELOF1*	0	1	0	0	14.25	24.30	53.25	92.50
ENSGACG00000006453	*RAB3D (1 of 2)*	0	1	0	0	37.42	32.98	51.26	62.75
ENSGACG00000006471	*TRAF3IP2*	0	1	0	0	14.06	10.65	21.36	13.76
ENSGACG00000006476	*ABCA12*	0	1	1	1	5.96	8.88	0.11	6.10
ENSGACG00000006511	*COL1A2*	0	1	0	0	10.22	30.72	16.89	13.05
ENSGACG00000006516	*COX5A (2 of 2)*	0	1	0	0	63.65	99.96	91.85	70.91
ENSGACG00000006524	-	1	1	0	0	4.59	0.42	0.08	0.18
ENSGACG00000006527	*EPHB1 (2 of 2)*	0	1	0	0	1.47	0.20	0.05	0.06
ENSGACG00000006530	*NRAP*	0	0	0	1	0.29	0.13	0.09	0.64
ENSGACG00000006539	*MYL1*	0	1	0	0	5.20	0.91	0.04	0.18
ENSGACG00000006541	*RER1*	0	1	0	0	33.60	54.81	61.35	74.54
ENSGACG00000006550	*SRPR*	0	1	0	0	23.07	25.33	51.31	58.35
ENSGACG00000006559	*ST3GAL4*	0	1	0	0	33.45	23.89	6.37	11.35
ENSGACG00000006570	*AMD1*	0	1	0	0	36.77	29.80	51.83	96.95
ENSGACG00000006579	-	0	1	0	0	79.34	167.48	291.55	242.03
ENSGACG00000006616	*SLC12A8*	1	1	0	0	8.78	1.14	1.07	1.20
ENSGACG00000006637	*COL24A1*	0	1	0	0	1.69	0.77	0.16	0.49
ENSGACG00000006638	*GRAMD1B*	1	1	0	0	26.45	6.07	5.59	9.04
ENSGACG00000006644	-	0	1	0	1	74.70	20.50	0.10	17.67
ENSGACG00000006647	*AP3M1*	0	1	0	0	9.34	15.89	17.07	14.97
ENSGACG00000006664	*EDAR*	0	1	0	0	2.86	0.33	0.06	0.05
ENSGACG00000006665	-	0	1	0	0	23.98	42.93	51.18	76.76
ENSGACG00000006676	*PRR13*	0	1	0	0	40.46	56.99	54.07	42.52
ENSGACG00000006684	*CHMP2B*	0	1	0	0	32.15	41.03	43.38	37.72
ENSGACG00000006688	*ATG4D (2 of 2)*	0	1	0	0	26.20	28.88	78.86	48.87
ENSGACG00000006692	*SLC35D2*	0	1	1	0	12.82	9.02	30.36	50.46
ENSGACG00000006707	*LRRC16A (1 of 2)*	0	0	0	1	8.63	5.54	7.59	47.13
ENSGACG00000006711	-	1	0	0	0	371.50	27531.70	92495.10	260.01
ENSGACG00000006714	-	1	0	0	0	31.17	111.53	29.04	21.71
ENSGACG00000006750	*ADAM8 (1 of 2)*	0	0	0	1	29.87	37.66	7.97	3.47
ENSGACG00000006759	*NUCB2 (2 of 2)*	0	1	1	0	41.64	20.85	97.11	33.20
ENSGACG00000006766	*MAN1A1*	0	1	0	0	11.06	7.66	14.76	25.55
ENSGACG00000006768	-	0	1	0	0	7.68	8.81	11.11	10.26
ENSGACG00000006771	*HMOX1 (2 of 2)*	0	1	0	0	32.18	37.77	3.98	3.80
ENSGACG00000006773	-	0	1	1	1	0.77	0.40	3.37	0.44
ENSGACG00000006777	*FUOM*	0	1	1	0	20.66	13.22	103.11	81.93
ENSGACG00000006785	*ECHS1*	0	0	0	1	73.92	72.32	44.14	172.71
ENSGACG00000006797	*FBXO47*	0	1	0	0	0.42	0.59	2.32	1.81
ENSGACG00000006801	*PLCE1*	0	1	0	0	5.20	1.15	0.84	1.79
ENSGACG00000006805	*SLC5A7 (1 of 2)*	0	1	1	0	7.18	3.07	13.48	9.95
ENSGACG00000006837	*NMT1 (2 of 2)*	1	0	0	0	1.57	22.95	5.64	0.85
ENSGACG00000006840	*TTC13*	0	1	0	0	21.70	22.37	31.92	27.30
ENSGACG00000006847	-	0	1	0	0	21.95	11.29	4.65	5.47
ENSGACG00000006849	*MPP6 (1 of 2)*	0	1	0	0	2.51	6.85	8.58	1.76
ENSGACG00000006856	*TMEM30A (2 of 2)*	0	1	0	0	35.61	51.19	62.00	41.47
ENSGACG00000006860	*C5orf51*	0	1	0	0	25.42	36.13	32.40	32.56
ENSGACG00000006868	-	0	1	0	0	8.90	8.13	16.72	10.16
ENSGACG00000006885	*C1orf131*	0	1	0	0	12.88	29.43	21.24	18.99
ENSGACG00000006921	*CXCL12 (2 of 2)*	0	0	0	1	1143.59	1247.81	304.94	42.77
ENSGACG00000006942	*GPNMB*	0	1	0	1	24.82	10.79	1.51	0.46
ENSGACG00000006959	*QPCT*	0	1	1	1	1.57	0.91	10.14	1.23
ENSGACG00000006960	-	0	1	1	1	0.02	0.00	4.80	0.05
ENSGACG00000006965	*MOCS3*	0	1	1	1	12.92	13.79	35.43	16.03
ENSGACG00000006967	*TBC1D5*	0	1	1	0	14.17	7.82	83.53	34.43
ENSGACG00000006972	*WDFY4*	0	1	0	0	17.23	9.87	2.96	6.86
ENSGACG00000006980	*PRKD3 (2 of 2)*	0	0	0	1	8.76	7.85	8.04	3.03
ENSGACG00000006983	*ITGA7*	0	1	1	1	1.03	0.74	8.66	2.32
ENSGACG00000006984	*NDRG1 (2 of 2)*	0	0	0	1	0.14	0.04	2.86	0.03
ENSGACG00000006995	*TMEM214*	0	1	0	0	36.01	38.19	82.07	108.59

ENSGACG00000007009	*VMP1*	0	1	0	0	31.02	33.07	54.09	46.59
ENSGACG00000007016	*TUBD1*	0	1	0	0	5.41	4.74	0.57	1.24
ENSGACG00000007033	*PDIA3*	0	1	0	0	177.69	418.46	1816.64	1529.73
ENSGACG00000007046	*SLC33A1*	0	1	1	0	10.32	14.81	32.47	37.28
ENSGACG00000007062	*GMPPA*	0	1	1	0	9.65	11.65	32.83	29.33
ENSGACG00000007071	*SSR3*	0	1	0	0	158.60	525.81	1177.62	1021.47
ENSGACG00000007073	*MYH10 (2 of 2)*	0	1	0	0	1.98	0.14	0.07	0.14
ENSGACG00000007080	*NDUFA4 (2 of 2)*	0	1	0	1	76.36	78.27	157.22	54.22
ENSGACG00000007091	*MFGE8 (2 of 2)*	0	1	0	0	14.50	6.26	2.08	3.28
ENSGACG00000007097	*DNMT3A (2 of 2)*	0	1	0	1	15.97	8.57	2.44	20.83
ENSGACG00000007110	-	0	0	1	0	0.21	0.04	0.40	0.11
ENSGACG00000007114	*PPIP5K1 (3 of 3)*	0	1	0	0	5.09	1.86	0.69	0.94
ENSGACG00000007117	*VEPH1*	0	1	0	0	5.54	1.71	0.41	2.01
ENSGACG00000007161	*SERPINI1*	0	1	1	1	19.90	12.41	238.91	32.76
ENSGACG00000007167	*ELOVL7*	0	0	0	1	10.43	2.11	3.42	0.65
ENSGACG00000007186	*MYO9A (1 of 2)*	0	1	0	0	2.64	1.27	0.25	0.28
ENSGACG00000007235	*TGFB1I1*	0	1	0	1	8.95	5.60	1.39	7.89
ENSGACG00000007238	*PPIB*	0	1	0	0	275.17	243.31	1999.21	1238.25
ENSGACG00000007280	*CLIC4*	0	1	0	0	54.93	17.16	9.29	11.86
ENSGACG00000007290	*KDELR3*	0	1	0	0	28.94	35.79	159.75	167.60
ENSGACG00000007298	-	0	0	0	1	70.03	145.10	46.54	16.51
ENSGACG00000007308	*PPRC1 (1 of 2)*	0	0	0	1	5.48	5.55	3.13	16.29
ENSGACG00000007312	*HIST1H1T*	1	0	0	0	9.76	191.89	19.67	4.40
ENSGACG00000007315	*MPEG1*	0	1	0	0	52.22	69.76	8.86	14.73
ENSGACG00000007323	-	0	1	0	0	119.28	46.03	5.94	27.97
ENSGACG00000007331	*PROM2*	0	1	0	0	83.21	46.87	292.51	67.75
ENSGACG00000007337	-	0	1	0	0	24.69	11.29	3.41	1.36
ENSGACG00000007338	*CCDC164*	0	1	0	0	28.85	6.20	3.37	7.32
ENSGACG00000007343	*COL9A2*	0	1	1	0	12.18	12.19	64.10	168.38
ENSGACG00000007345	*GRID2*	0	1	0	0	1.01	4.61	10.45	7.70
ENSGACG00000007346	*PHACTR4 (1 of 2)*	0	1	0	0	4.95	1.85	0.72	1.71
ENSGACG00000007348	-	0	1	1	0	0.34	0.24	2.16	0.49
ENSGACG00000007397	*ATP2A1 (2 of 2)*	0	1	0	1	22.12	0.58	0.10	0.70
ENSGACG00000007411	-	0	1	1	0	70.65	41.53	143.02	47.66
ENSGACG00000007419	-	0	1	0	0	18.31	5.45	2.11	5.58
ENSGACG00000007434	*PTPN9*	0	0	1	0	6.61	1.85	6.99	11.52
ENSGACG00000007442	-	0	1	0	0	1.36	5.80	7.02	7.89
ENSGACG00000007466	-	0	0	0	1	3.79	0.88	0.58	5.26
ENSGACG00000007470	-	0	0	0	1	4.21	0.60	0.71	5.38
ENSGACG00000007483	-	0	1	0	1	4.03	0.89	0.58	5.25
ENSGACG00000007493	*GPR32 (1 of 3)*	0	0	1	0	12.29	14.64	1.16	0.99
ENSGACG00000007514	*ESR2*	0	1	0	0	6.71	11.00	22.97	18.01
ENSGACG00000007518	-	0	1	0	0	512.27	664.84	31.01	62.19
ENSGACG00000007547	-	0	1	0	0	2.27	0.55	0.13	1.02
ENSGACG00000007570	*SLC6A19 (1 of 3)*	0	1	0	0	26.92	13.19	1.03	14.09
ENSGACG00000007583	*ERRFI1 (1 of 2)*	0	1	0	0	22.89	5.89	2.69	4.60
ENSGACG00000007592	*REPS1*	0	1	0	0	16.19	12.12	27.82	20.89
ENSGACG00000007595	*C14orf1*	0	1	0	1	5.48	15.92	32.68	8.84
ENSGACG00000007615	*NIF3L1*	0	1	0	0	9.30	8.49	14.27	12.14
ENSGACG00000007628	*TEF (1 of 2)*	0	1	0	0	22.36	5.49	3.28	5.41
ENSGACG00000007646	*PRODH2*	0	1	0	0	36.92	22.86	5.44	6.48
ENSGACG00000007652	*EIF3CL*	0	1	0	0	85.31	106.08	106.24	119.64
ENSGACG00000007659	*IGFLR1*	0	1	0	0	54.75	31.15	9.50	24.45
ENSGACG00000007663	*ST13*	0	1	0	0	94.46	106.73	117.40	97.59
ENSGACG00000007665	*SFRP5*	0	1	0	1	3.52	8.37	6.18	2.09
ENSGACG00000007684	*XPNPEP3*	0	1	0	0	9.36	9.76	14.30	16.77
ENSGACG00000007711	*NPHS1*	1	1	0	0	45.74	7.99	3.24	8.81
ENSGACG00000007715	*EDN2*	0	1	0	1	3.25	2.55	6.81	0.67
ENSGACG00000007730	-	0	1	0	0	11.93	2.26	0.05	0.48
ENSGACG00000007744	*GPI (2 of 2)*	0	1	0	0	322.16	249.95	54.97	35.70
ENSGACG00000007752	*GSTZ1*	0	0	1	0	54.67	65.20	4.06	13.04
ENSGACG00000007754	*STARD10*	0	1	0	0	48.04	37.36	111.91	56.07
ENSGACG00000007762	*ALOX5*	0	0	0	1	93.95	126.36	30.21	10.15
ENSGACG00000007763	*CCDC176*	0	0	0	1	9.59	3.21	0.89	3.96
ENSGACG00000007766	*NR2F6 (2 of 2)*	0	1	0	0	29.05	7.17	2.90	10.05
ENSGACG00000007773	*PTPRU (1 of 2)*	0	1	0	1	1.20	0.23	0.08	0.83

ENSGACG00000007782	-	1	1	0	0	23.32	1.09	1.47	4.23
ENSGACG00000007786	*SNX20*	0	1	0	0	17.78	12.69	1.59	1.66
ENSGACG00000007789	-	0	1	0	0	38.33	1.65	3.32	3.16
ENSGACG00000007794	-	0	1	0	0	24.25	2.15	2.05	3.96
ENSGACG00000007797	*VKORC1*	0	1	0	0	24.26	25.56	81.75	92.49
ENSGACG00000007837	-	0	1	0	0	17.16	4.81	2.50	2.54
ENSGACG00000007862	*ACAP3 (2 of 2)*	0	1	1	1	0.95	0.24	3.45	0.43
ENSGACG00000007929	*NPHS2*	0	1	0	0	41.48	13.99	3.59	11.57
ENSGACG00000007935	*SLC16A4*	0	1	0	1	69.16	11.02	0.06	22.34
ENSGACG00000007945	*IRF3*	1	0	0	0	4.46	17.40	3.88	1.96
ENSGACG00000007981	*ASPG*	0	1	0	0	38.55	13.83	5.22	12.15
ENSGACG00000007983	*ARRDC2*	0	1	0	0	95.40	39.18	12.99	17.25
ENSGACG00000007986	*NR1D2 (1 of 2)*	0	1	0	0	25.84	8.98	3.80	9.33
ENSGACG00000007988	*KIF26A (1 of 2)*	0	1	0	0	9.95	4.03	2.33	6.34
ENSGACG00000008019	-	0	1	0	0	11.08	6.82	1.21	2.91
ENSGACG00000008022	-	0	1	0	0	6.49	2.66	1.15	2.05
ENSGACG00000008048	*SERPINA10 (1 of 2)*	0	1	0	0	22.81	27.12	2.30	15.87
ENSGACG00000008055	-	0	1	0	0	6.77	18.85	0.66	8.91
ENSGACG00000008059	-	0	1	0	0	0.77	0.95	1.97	1.02
ENSGACG00000008064	-	0	1	0	0	4.75	12.30	0.46	3.76
ENSGACG00000008066	-	0	0	1	0	6.36	20.71	2.62	2.26
ENSGACG00000008067	*MACC1*	0	1	1	1	2.38	2.43	23.55	7.46
ENSGACG00000008074	*KIF24*	0	1	0	0	2.76	2.27	9.27	14.18
ENSGACG00000008090	*NAPSA*	0	1	0	0	80.65	56.79	14.16	18.12
ENSGACG00000008094	*OTUB2*	0	0	0	1	25.97	23.04	30.37	16.02
ENSGACG00000008095	*MARK1 (2 of 2)*	0	1	1	1	6.01	3.36	19.60	4.89
ENSGACG00000008097	*PSMD9*	0	1	0	0	19.53	36.99	26.51	26.49
ENSGACG00000008109	*KIAA0895*	0	1	0	0	16.20	4.35	1.15	1.65
ENSGACG00000008112	*SAYSD1*	0	1	0	0	13.05	19.98	27.48	28.10
ENSGACG00000008118	*ANLN*	0	1	0	0	14.51	12.32	1.75	1.30
ENSGACG00000008132	*PGPEP1 (1 of 2)*	0	1	0	0	6.69	5.50	10.44	11.69
ENSGACG00000008133	*FKBP14 (2 of 2)*	0	1	1	0	3.43	6.60	16.64	13.90
ENSGACG00000008160	*MYL9*	0	0	0	1	38.19	88.08	44.55	18.97
ENSGACG00000008164	*PARG (1 of 2)*	0	1	0	0	7.12	12.64	15.47	11.79
ENSGACG00000008168	*FRZB*	0	1	0	0	5.01	11.61	15.04	8.88
ENSGACG00000008173	*RAB3A (2 of 2)*	0	1	1	0	0.99	0.71	6.69	4.30
ENSGACG00000008189	*DNAJC10*	0	1	0	0	19.00	30.82	469.40	386.66
ENSGACG00000008217	*ANGPTL4 (2 of 2)*	0	1	0	1	27.80	3.36	1.16	21.97
ENSGACG00000008225	*GRB10 (2 of 2)*	1	0	0	0	26.31	5.27	7.47	15.00
ENSGACG00000008228	*P2RY2 (2 of 2)*	0	1	0	0	7.58	9.33	11.29	16.24
ENSGACG00000008233	*P2RY2 (1 of 2)*	0	1	0	0	21.03	22.24	27.83	47.99
ENSGACG00000008243	*RILPL2*	0	1	0	0	18.91	28.86	54.18	35.95
ENSGACG00000008249	-	0	1	0	0	44.10	66.76	61.24	52.25
ENSGACG00000008299	*TMEM150B*	0	1	0	0	94.81	76.18	173.54	189.29
ENSGACG00000008307	-	0	1	0	0	0.92	2.07	3.18	1.22
ENSGACG00000008312	*STX18*	0	1	0	0	10.33	17.34	28.05	21.74
ENSGACG00000008313	*PDGFB (1 of 2)*	0	1	1	0	1.82	1.45	23.24	8.56
ENSGACG00000008349	*TNNI2 (4 of 5)*	0	1	0	0	25.35	2.85	0.02	0.09
ENSGACG00000008361	*VPS53*	0	1	0	0	10.29	13.76	13.06	9.17
ENSGACG00000008365	-	0	1	1	0	39.21	58.97	826.99	219.18
ENSGACG00000008370	*ABLIM1 (2 of 2)*	1	0	0	0	8.13	1.15	0.84	0.67
ENSGACG00000008374	*MAT2A (1 of 2)*	0	1	1	0	57.55	80.23	813.40	244.27
ENSGACG00000008379	*MPRIP*	0	1	0	0	12.65	3.61	2.00	3.34
ENSGACG00000008384	*TNNT3 (1 of 2)*	0	1	0	0	26.84	2.54	0.06	0.28
ENSGACG00000008395	*SYT14 (2 of 2)*	0	1	1	1	5.25	4.41	16.13	7.04
ENSGACG00000008433	*SLC24A4*	0	0	1	0	6.62	0.76	3.48	3.72
ENSGACG00000008460	*HIF1AN*	0	1	0	0	3.53	4.79	7.40	5.15
ENSGACG00000008463	*C7orf41*	0	1	0	0	8.44	0.57	0.40	0.18
ENSGACG00000008494	-	0	1	0	0	23.91	8.85	4.35	6.99
ENSGACG00000008515	-	0	1	0	0	163.93	253.58	335.77	190.16
ENSGACG00000008542	*TPP1*	0	1	0	0	137.23	104.74	28.95	37.83
ENSGACG00000008543	*TMED6*	0	1	0	0	4.57	2.00	0.21	0.59
ENSGACG00000008558	*COMP*	1	1	0	0	16.88	1.19	1.62	1.17
ENSGACG00000008583	*COX6B1*	0	0	0	1	117.65	227.98	138.89	26.71
ENSGACG00000008590	*GALNT6*	0	1	0	0	28.01	45.59	220.31	119.71
ENSGACG00000008591	*ZDHHC11*	0	1	1	0	8.58	3.79	44.19	30.03

ENSGACG00000008596	*DDIT4 (2 of 2)*	0	0	0	1	0.54	0.72	1.55	0.12
ENSGACG00000008613	*EVL (2 of 2)*	0	1	0	0	3.03	2.00	5.37	5.77
ENSGACG00000008617	*PTPRZ1 (1 of 2)*	1	1	0	0	2.45	0.15	0.10	0.99
ENSGACG00000008622	*MAST3 (1 of 2)*	0	0	0	1	12.28	8.46	8.05	4.00
ENSGACG00000008639	*PKDCC*	0	1	1	0	4.63	3.89	9.87	8.78
ENSGACG00000008643	*B3GNT3*	0	0	0	1	32.07	34.69	33.65	8.11
ENSGACG00000008644	*PPA1 (1 of 2)*	1	1	0	0	14.88	59.88	41.38	50.03
ENSGACG00000008645	*ARHGAP4 (3 of 3)*	0	1	1	0	18.60	21.87	3.32	6.76
ENSGACG00000008662	*SSR4*	0	1	0	0	81.25	181.42	528.98	481.70
ENSGACG00000008665	-	0	1	0	0	8.66	10.16	20.78	30.87
ENSGACG00000008673	*CPD*	0	1	1	0	11.22	8.26	27.32	12.96
ENSGACG00000008675	-	0	1	1	0	10.68	11.23	1.93	6.27
ENSGACG00000008681	*AVPR2 (1 of 2)*	0	1	1	0	3.86	2.42	24.89	7.65
ENSGACG00000008709	*IDH3G (1 of 2)*	0	1	0	0	47.95	45.65	62.98	48.93
ENSGACG00000008721	-	0	0	0	1	7.41	3.46	3.71	12.10
ENSGACG00000008724	*IVD*	0	1	0	0	51.46	43.01	69.62	76.43
ENSGACG00000008730	*SPAM1*	0	0	0	1	1.27	0.58	0.06	5.35
ENSGACG00000008740	*DHX58*	1	0	0	0	4.78	14.74	2.79	1.79
ENSGACG00000008760	*KIAA1328 (2 of 2)*	0	1	1	1	4.18	7.53	39.38	4.46
ENSGACG00000008773	*PTPRS (2 of 2)*	0	1	0	0	6.03	1.62	1.34	1.96
ENSGACG00000008778	*CMKLR1 (1 of 2)*	0	1	0	0	10.71	9.75	1.46	2.03
ENSGACG00000008784	*FICD*	0	1	0	0	3.85	3.80	6.98	6.52
ENSGACG00000008786	*STEAP4 (2 of 2)*	0	1	1	0	0.14	0.26	1.43	0.45
ENSGACG00000008801	*ISCU*	0	1	0	0	62.22	126.29	108.90	99.98
ENSGACG00000008805	*ST3GAL1 (1 of 8)*	0	1	1	0	5.38	5.36	23.68	14.95
ENSGACG00000008811	*DLG5 (1 of 2)*	0	1	1	0	13.50	6.56	15.85	25.11
ENSGACG00000008826	*ST3GAL1 (7 of 8)*	0	0	1	0	0.46	0.48	1.92	2.12
ENSGACG00000008827	*GTF2H5*	0	1	0	0	138.97	377.69	253.38	223.76
ENSGACG00000008833	*TMEM181*	0	1	0	0	10.77	10.04	17.34	12.22
ENSGACG00000008867	*LEPREL4 (1 of 2)*	0	1	0	0	1.39	1.69	3.65	2.19
ENSGACG00000008882	*CEP57L1*	0	1	0	0	14.91	14.75	2.62	4.46
ENSGACG00000008890	*ZNF503 (1 of 2)*	0	1	0	0	17.16	8.50	2.24	7.39
ENSGACG00000008898	*CERK (1 of 2)*	0	1	0	0	11.16	3.36	1.09	3.09
ENSGACG00000008923	*IAH1*	0	1	0	0	16.95	34.61	42.15	28.48
ENSGACG00000008936	*SLC45A4 (2 of 2)*	0	0	0	1	5.64	3.75	2.68	12.71
ENSGACG00000008943	*GPR20 (1 of 2)*	0	1	1	0	0.48	0.39	3.07	1.34
ENSGACG00000008951	*DGAT1 (2 of 2)*	0	1	0	0	12.59	12.92	21.95	24.80
ENSGACG00000008955	*ASAP2 (1 of 2)*	0	0	0	1	6.49	2.77	1.50	5.73
ENSGACG00000008967	*DNAH8*	1	0	0	0	0.00	0.93	0.03	0.01
ENSGACG00000008970	*NCL*	0	0	0	1	232.20	235.43	112.77	349.33
ENSGACG00000008974	*NSUN2*	0	0	0	1	13.57	11.03	6.00	21.52
ENSGACG00000008995	*HSPE1*	1	0	0	0	113.89	537.46	103.25	205.36
ENSGACG00000009011	*ALG2*	0	1	0	0	17.61	17.00	42.59	43.48
ENSGACG00000009015	*CTTNBP2*	1	1	0	0	4.95	0.79	0.33	0.72
ENSGACG00000009017	*RFTN2*	0	1	1	1	2.36	1.84	7.57	0.70
ENSGACG00000009022	*SEC61B*	0	1	0	0	67.65	244.35	590.32	419.57
ENSGACG00000009026	*PLCL1*	0	1	0	0	1.49	3.25	7.27	4.63
ENSGACG00000009034	*PTPRA*	0	1	0	0	17.25	11.38	34.31	35.94
ENSGACG00000009041	*STX17*	0	1	1	0	8.19	8.09	19.07	12.61
ENSGACG00000009047	*DNAJB11*	0	1	0	0	17.37	21.22	42.25	44.91
ENSGACG00000009048	*ERP44*	0	1	0	0	22.55	31.91	64.53	62.22
ENSGACG00000009056	*INVS*	0	1	0	0	2.71	3.37	3.85	2.91
ENSGACG00000009062	-	0	1	0	0	7.81	9.63	22.60	28.10
ENSGACG00000009069	*NMUR1 (1 of 2)*	0	1	1	1	0.90	0.53	3.15	0.17
ENSGACG00000009089	*OAF*	0	1	0	0	8.15	2.83	1.19	1.78
ENSGACG00000009104	*DNAJC22*	0	1	0	0	12.08	7.53	1.51	3.65
ENSGACG00000009130	*LCMT1*	0	1	0	0	12.43	12.66	21.15	11.07
ENSGACG00000009151	*PRKG1*	0	1	0	0	17.21	5.22	1.97	2.91
ENSGACG00000009159	*KCNH3*	1	1	0	0	7.20	1.14	0.75	1.42
ENSGACG00000009169	*ESRP1*	0	1	0	0	14.33	8.26	16.70	22.28
ENSGACG00000009178	*MINPP1 (1 of 2)*	0	1	1	0	12.17	8.26	28.66	29.61
ENSGACG00000009188	-	1	0	0	0	4.35	106.62	10.21	4.13
ENSGACG00000009190	*MET*	1	1	0	0	15.07	1.93	1.04	1.87
ENSGACG00000009194	*PM20D2*	0	1	1	0	2.26	1.75	9.92	17.06
ENSGACG00000009195	*PAPSS2*	0	1	0	0	7.84	8.77	15.52	9.24
ENSGACG00000009200	-	1	0	0	0	6.35	97.30	10.94	5.28

ENSGACG00000009214	*CPA1*	0	0	0	1	220.60	196.66	42.33	11.94
ENSGACG00000009227	*ESYT1 (1 of 2)*	0	1	0	0	17.95	8.62	1.75	1.81
ENSGACG00000009261	-	0	1	0	0	5.51	5.11	30.62	16.92
ENSGACG00000009262	*SEPN1*	0	1	1	0	9.29	7.11	15.26	28.02
ENSGACG00000009268	-	0	1	1	0	12.29	19.14	106.23	43.77
ENSGACG00000009279	*ZNF593*	0	1	0	0	42.40	122.41	57.72	99.95
ENSGACG00000009283	*SLC6A18 (2 of 3)*	1	1	0	0	29.70	3.59	2.10	10.14
ENSGACG00000009285	*TRAM2*	0	1	0	0	10.34	11.84	17.25	12.61
ENSGACG00000009289	*CCDC80*	0	1	0	0	5.54	7.59	9.84	4.31
ENSGACG00000009290	-	0	0	1	0	11.73	44.46	1.45	0.77
ENSGACG00000009292	-	0	1	0	0	25.27	24.23	38.85	27.72
ENSGACG00000009295	*ATP13A3*	0	1	0	0	349.62	94.46	54.03	191.00
ENSGACG00000009299	*ABCB4*	1	1	0	0	84.30	10.62	2.09	4.41
ENSGACG00000009313	*IGKC (13 of 24)*	0	0	0	1	38.55	178.81	11.87	2.68
ENSGACG00000009315	*IGKC (9 of 24)*	0	0	0	1	26.12	110.68	7.58	2.43
ENSGACG00000009327	*IGKC (21 of 24)*	0	0	0	1	28.40	126.11	8.52	1.89
ENSGACG00000009345	-	0	0	1	0	4.10	1.52	4.68	2.22
ENSGACG00000009349	*TMPRSS4*	0	1	1	0	29.68	18.93	55.26	81.10
ENSGACG00000009350	-	0	1	1	0	0.26	0.33	1.35	1.60
ENSGACG00000009353	*MAOA*	0	1	0	0	25.91	7.94	4.12	50.33
ENSGACG00000009354	-	0	0	0	1	12.51	0.95	0.18	6.17
ENSGACG00000009358	*ZNF683*	0	1	1	1	1.72	2.96	25.96	7.54
ENSGACG00000009363	-	0	1	0	0	25.45	16.21	3.22	3.35
ENSGACG00000009374	*TECPR1 (1 of 2)*	0	1	0	0	9.52	7.84	14.67	21.34
ENSGACG00000009393	-	0	1	1	0	0.09	0.07	3.09	0.12
ENSGACG00000009400	*EOGT*	0	0	0	1	2.34	1.45	0.60	3.39
ENSGACG00000009403	*BHLHA15*	0	1	1	0	3.10	4.10	25.60	25.65
ENSGACG00000009409	-	0	1	0	0	12.99	3.71	0.00	0.02
ENSGACG00000009413	*WARS*	0	1	0	0	7.48	13.97	9.76	9.48
ENSGACG00000009423	-	0	1	0	1	12.64	6.70	0.71	24.83
ENSGACG00000009441	*SLC25A29 (2 of 2)*	0	1	0	0	32.02	16.84	5.28	5.84
ENSGACG00000009463	*CSAD*	0	0	0	1	64.48	48.89	21.37	4.00
ENSGACG00000009469	*EGLN2*	0	1	1	1	4.30	3.08	9.59	3.15
ENSGACG00000009472	-	0	0	1	0	40.96	270.86	6.07	4.34
ENSGACG00000009475	*IGKC (4 of 24)*	0	0	0	1	40.17	181.57	12.61	2.83
ENSGACG00000009476	-	0	0	0	1	8.10	22.36	4.14	0.88
ENSGACG00000009480	*IGKC (1 of 24)*	0	0	0	1	74.46	216.31	18.90	2.84
ENSGACG00000009487	-	0	1	1	0	0.90	2.49	8.55	5.68
ENSGACG00000009488	*NIPAL3*	0	1	0	0	14.89	11.44	18.05	15.08
ENSGACG00000009493	*IGKC (3 of 24)*	0	0	0	1	39.37	179.87	12.26	2.61
ENSGACG00000009499	-	0	0	0	1	15.41	78.06	5.53	1.11
ENSGACG00000009503	*IGKC (5 of 24)*	0	0	0	1	38.53	167.97	10.42	2.36
ENSGACG00000009504	*AIM1 (2 of 2)*	0	1	0	0	6.51	5.00	1.08	1.56
ENSGACG00000009509	*CNKSR1*	0	1	0	0	14.90	4.33	2.21	3.93
ENSGACG00000009510	-	0	1	0	1	13.18	13.77	16.45	7.54
ENSGACG00000009522	*TMEM98*	0	1	0	0	4.12	10.05	25.81	55.19
ENSGACG00000009539	*RAB4B*	0	1	0	0	30.00	23.69	49.85	73.22
ENSGACG00000009553	*RASSF9*	0	1	0	0	5.73	1.74	0.65	0.86
ENSGACG00000009560	*PVRL2*	0	1	0	0	10.44	4.53	1.80	2.38
ENSGACG00000009562	*SLC6A15*	0	1	0	1	2.86	2.01	5.14	0.34
ENSGACG00000009566	-	0	1	1	1	11.88	8.21	35.91	15.45
ENSGACG00000009586	*PDIA6*	0	1	1	0	61.83	96.20	579.35	767.44
ENSGACG00000009601	*VARS*	0	1	0	0	14.66	19.75	17.50	25.59
ENSGACG00000009604	*CALM3*	0	1	0	0	47.94	54.57	55.69	80.55
ENSGACG00000009622	*SLC4A1 (1 of 2)*	1	0	0	0	15.85	128.79	43.24	6.51
ENSGACG00000009623	*RPN1*	0	1	0	0	33.06	58.01	103.16	194.86
ENSGACG00000009626	-	1	1	0	0	3.00	0.42	0.19	0.34
ENSGACG00000009644	*LRRC16A (2 of 2)*	0	1	0	0	9.95	4.40	1.45	3.03
ENSGACG00000009674	*PTAFR*	0	0	0	1	9.96	16.73	4.73	1.44
ENSGACG00000009676	-	0	0	0	1	23.20	62.95	10.24	4.98
ENSGACG00000009684	*SLC35B1*	0	1	0	0	21.45	33.72	132.02	107.05
ENSGACG00000009689	*SMC1B*	1	0	1	0	0.01	0.60	0.02	0.02
ENSGACG00000009691	*NAV1 (2 of 2)*	0	1	0	0	11.82	5.57	2.56	4.20
ENSGACG00000009702	*COL14A1 (1 of 2)*	0	1	0	0	1.88	0.38	0.17	0.43
ENSGACG00000009707	*SEC61A1 (1 of 2)*	0	1	0	0	45.48	71.73	253.94	275.43
ENSGACG00000009715	*SLC16A6 (2 of 2)*	0	0	0	1	23.99	22.80	18.90	60.09

ENSGACG00000009722	*RUNDC3A (1 of 2)*	0	1	0	1	3.74	7.63	8.00	3.63
ENSGACG00000009729	*AMPD3 (1 of 2)*	0	1	0	1	44.09	25.74	7.03	3.09
ENSGACG00000009732	*QPCTL*	0	1	1	0	4.70	7.57	22.14	21.58
ENSGACG00000009733	-	1	0	0	0	8.23	0.95	1.50	1.55
ENSGACG00000009747	*TNNT2 (1 of 2)*	0	1	1	0	0.07	0.34	2.27	0.59
ENSGACG00000009748	*SWAP70 (1 of 2)*	0	1	0	0	31.57	22.26	4.79	2.69
ENSGACG00000009752	*PKP1 (1 of 2)*	0	0	1	0	3.12	0.91	11.99	2.45
ENSGACG00000009754	*RIOK2*	0	1	0	0	16.07	22.96	22.44	27.10
ENSGACG00000009757	*DDR1*	0	0	0	1	24.44	14.31	13.00	49.29
ENSGACG00000009759	*MYOF (2 of 2)*	0	1	0	1	43.94	20.79	7.44	25.47
ENSGACG00000009763	-	1	0	0	0	1.78	16.87	2.78	2.08
ENSGACG00000009765	*GNL1*	0	1	0	0	19.62	17.35	22.53	21.79
ENSGACG00000009768	-	0	0	1	0	4.73	49.91	5.26	2.77
ENSGACG00000009770	-	1	0	0	0	8.82	100.56	26.47	5.15
ENSGACG00000009779	-	0	1	1	1	2.28	1.15	6.00	0.71
ENSGACG00000009781	-	0	1	1	0	11.97	13.60	27.72	39.26
ENSGACG00000009785	-	0	1	0	0	4.36	5.10	17.78	4.92
ENSGACG00000009852	-	0	0	0	1	28.92	63.75	11.07	4.49
ENSGACG00000009860	-	0	0	0	1	0.10	0.05	0.02	1.32
ENSGACG00000009865	*RHAG*	1	0	0	0	35.16	127.51	22.41	10.44
ENSGACG00000009874	*LMO2*	0	1	0	0	33.77	34.30	5.13	4.42
ENSGACG00000009891	*SRPRB*	0	1	0	0	36.87	48.44	70.47	79.55
ENSGACG00000009892	*TSPAN18*	0	1	0	0	18.44	3.98	2.95	5.65
ENSGACG00000009898	*ACE*	0	1	0	0	46.83	29.60	6.49	15.55
ENSGACG00000009919	-	0	0	0	1	0.17	0.06	0.05	0.00
ENSGACG00000009930	*JMY (1 of 2)*	0	1	1	0	0.62	0.21	2.12	2.27
ENSGACG00000009934	*LHFPL3 (2 of 2)*	1	1	0	0	0.27	5.30	3.45	3.04
ENSGACG00000009937	-	0	1	0	0	21.07	55.41	95.32	173.91
ENSGACG00000009941	*DIMT1*	0	1	0	0	11.80	14.97	14.64	32.84
ENSGACG00000009945	-	0	0	1	1	0.41	0.20	2.28	0.21
ENSGACG00000009959	*RELN*	0	1	0	0	1.78	0.67	0.13	0.58
ENSGACG00000009969	*SLC16A9*	0	1	0	0	16.50	22.71	55.26	62.48
ENSGACG00000009989	*CASP12*	0	0	1	0	38.64	76.37	8.82	5.97
ENSGACG00000010010	*EZR (1 of 2)*	0	1	0	0	36.76	23.44	45.29	18.20
ENSGACG00000010028	*NMD3*	0	1	0	0	27.86	48.21	36.22	57.42
ENSGACG00000010029	-	0	1	0	0	11.67	17.75	27.31	31.44
ENSGACG00000010058	*UBE2D1*	1	0	0	0	38.13	141.21	48.93	31.99
ENSGACG00000010067	*HSP90B1*	0	1	1	0	131.53	131.28	820.41	981.53
ENSGACG00000010070	*IQGAP2*	0	1	0	0	85.07	59.02	110.00	82.00
ENSGACG00000010071	*MIA2*	0	1	1	0	11.62	7.00	28.18	34.16
ENSGACG00000010074	*MIA3*	0	1	0	0	29.08	19.16	36.08	45.78
ENSGACG00000010092	*PPM1L (1 of 2)*	0	1	0	0	16.86	16.28	89.21	81.15
ENSGACG00000010100	*STXBP4*	0	0	0	1	10.16	5.83	12.68	5.07
ENSGACG00000010117	-	0	0	1	0	1.26	0.81	3.15	1.78
ENSGACG00000010120	*ANKFN1*	0	0	1	0	0.81	0.51	1.72	1.19
ENSGACG00000010121	*LPAR2 (2 of 2)*	0	1	0	0	14.33	9.85	2.01	1.63
ENSGACG00000010131	*PTTG1IP (2 of 2)*	0	1	1	1	21.32	11.51	61.14	12.23
ENSGACG00000010155	-	0	1	0	0	10.04	2.88	0.97	3.45
ENSGACG00000010163	-	0	1	0	0	16.96	3.47	1.23	3.00
ENSGACG00000010182	-	0	0	0	1	8.50	31.54	2.89	0.44
ENSGACG00000010186	*FGD6 (2 of 2)*	0	1	0	0	3.72	0.77	0.17	0.29
ENSGACG00000010197	-	1	1	0	0	2.96	0.24	0.06	0.14
ENSGACG00000010201	*DAPK2 (2 of 2)*	0	1	0	0	41.32	23.63	6.24	6.97
ENSGACG00000010213	-	0	1	0	0	23.34	9.82	3.63	1.86
ENSGACG00000010215	-	0	1	0	1	20.14	7.78	2.86	0.67
ENSGACG00000010227	*PTS*	0	1	0	0	36.02	9.25	3.17	11.82
ENSGACG00000010230	*CIB2 (1 of 2)*	0	1	1	0	1.06	0.59	5.37	1.91
ENSGACG00000010262	*DNMT3L (2 of 2)*	0	0	1	1	1.05	0.19	0.81	0.04
ENSGACG00000010273	*DNMT3B*	0	0	0	1	12.52	13.97	9.17	32.25
ENSGACG00000010275	-	0	1	0	0	17.27	6.10	2.24	6.23
ENSGACG00000010278	*NCOA7 (1 of 2)*	0	1	0	0	9.97	4.26	1.37	2.15
ENSGACG00000010302	*GPR149*	0	1	0	0	1.28	0.19	0.04	0.08
ENSGACG00000010308	-	0	1	0	0	14.53	4.61	1.04	3.66
ENSGACG00000010324	*TPD52L2*	0	1	0	0	26.27	21.68	42.46	39.19
ENSGACG00000010338	*HTRA1 (1 of 2)*	0	0	0	1	0.85	1.04	0.29	2.98
ENSGACG00000010360	*LBH*	0	1	1	0	30.00	24.42	74.49	121.28

ENSGACG00000010368	*BMP4*	0	1	0	0	5.35	1.38	0.47	1.11
ENSGACG00000010376	-	0	1	1	0	0.29	0.11	12.44	0.76
ENSGACG00000010405	*LAMA5*	0	1	1	0	7.93	3.20	10.04	15.30
ENSGACG00000010420	*BOP1*	0	1	0	0	16.92	29.64	20.37	34.94
ENSGACG00000010439	*SLC29A1 (1 of 2)*	0	1	0	1	52.34	48.72	74.44	34.13
ENSGACG00000010450	*TMED10*	0	1	0	0	104.75	138.22	313.85	345.72
ENSGACG00000010455	-	1	0	0	1	43.37	204.86	45.79	19.47
ENSGACG00000010460	-	0	1	0	0	5.15	1.86	0.65	1.13
ENSGACG00000010478	-	0	0	0	1	1.58	5.69	1.16	0.20
ENSGACG00000010510	-	1	1	0	0	11.64	0.97	0.24	0.28
ENSGACG00000010522	*AKT3 (1 of 2)*	0	1	1	1	7.26	2.94	10.65	1.86
ENSGACG00000010559	*LYSMD3*	0	1	0	0	8.18	10.57	15.94	12.80
ENSGACG00000010560	*CERS1*	0	0	0	1	8.52	5.65	1.60	10.14
ENSGACG00000010568	*COPE*	0	1	0	0	41.96	76.40	85.81	87.05
ENSGACG00000010579	-	0	1	0	0	6.61	1.94	0.68	0.19
ENSGACG00000010588	*FLVCR2 (2 of 2)*	0	1	0	0	11.93	7.70	1.74	4.13
ENSGACG00000010607	*BCO2 (3 of 3)*	1	0	0	0	22.70	4.17	4.76	2.18
ENSGACG00000010609	*PTK2B (1 of 2)*	0	1	0	0	13.90	8.30	17.04	15.64
ENSGACG00000010610	-	0	1	0	0	10.66	23.72	31.08	39.91
ENSGACG00000010612	*C19orf10*	0	1	0	0	16.58	29.79	45.27	58.83
ENSGACG00000010649	*C16orf80 (2 of 3)*	1	0	0	0	0.00	2.60	0.19	0.00
ENSGACG00000010678	*ACSF2 (2 of 2)*	0	1	0	0	100.25	147.21	367.43	588.19
ENSGACG00000010690	*TMEM2*	0	1	1	0	10.10	8.44	24.89	20.63
ENSGACG00000010711	*ZBTB16 (2 of 2)*	0	1	1	1	0.11	0.12	3.09	0.41
ENSGACG00000010725	*GCNT3 (2 of 2)*	0	1	0	1	6.59	1.38	0.64	2.58
ENSGACG00000010729	*GCNT3 (1 of 2)*	0	1	0	0	33.11	11.57	4.65	13.34
ENSGACG00000010732	-	0	1	0	0	42.71	9.22	2.22	8.86
ENSGACG00000010733	*RPN2*	0	1	0	0	43.78	89.47	137.34	186.81
ENSGACG00000010734	*OTUD7A*	0	1	0	1	7.20	2.22	0.35	3.10
ENSGACG00000010737	*PIH1D2*	0	1	1	0	7.15	8.87	25.69	22.05
ENSGACG00000010738	*SLC3A1*	0	1	0	0	47.88	63.64	311.33	143.43
ENSGACG00000010740	-	0	1	1	0	15.51	18.33	49.39	38.11
ENSGACG00000010742	-	0	1	1	0	17.59	8.77	68.01	23.15
ENSGACG00000010749	*SLCO3A1 (2 of 2)*	0	1	0	0	17.97	6.14	2.85	4.31
ENSGACG00000010798	-	0	0	1	1	9.70	1.99	13.20	5.02
ENSGACG00000010845	*TTYH2 (2 of 2)*	0	1	0	1	0.13	0.32	0.80	0.04
ENSGACG00000010863	*BLM*	0	1	0	0	16.22	4.88	2.28	3.85
ENSGACG00000010876	*SLC13A5 (1 of 2)*	1	0	0	0	45.93	3.17	1.23	0.81
ENSGACG00000010910	-	0	1	0	1	6.71	36.90	71.02	11.01
ENSGACG00000010911	*SLC28A2*	0	0	1	0	83.70	29.67	134.15	60.71
ENSGACG00000010917	-	0	1	0	0	27.31	7.73	5.09	7.59
ENSGACG00000010927	-	0	1	0	1	489.93	189.08	20.75	419.58
ENSGACG00000010939	*GFPT1*	0	1	0	0	18.44	27.79	52.75	46.41
ENSGACG00000010950	*TRIM35 (27 of 28)*	0	0	0	1	8.87	10.51	5.30	1.85
ENSGACG00000010952	-	0	0	0	1	18.49	18.49	11.61	3.58
ENSGACG00000010955	-	0	1	1	0	21.96	25.71	57.47	43.52
ENSGACG00000010958	*PLD1 (1 of 2)*	0	1	0	0	14.41	11.33	26.59	29.76
ENSGACG00000010962	*MFSD4 (2 of 2)*	0	1	1	0	18.83	20.42	124.96	42.21
ENSGACG00000010967	*TNIK (2 of 2)*	0	1	0	0	3.37	1.00	0.41	1.07
ENSGACG00000010968	*SLC2A12*	1	1	0	0	72.58	15.04	5.70	10.53
ENSGACG00000010972	*USH1G (2 of 2)*	0	0	0	1	1.18	0.58	2.55	0.36
ENSGACG00000010975	-	1	1	0	0	19.18	3.09	3.50	2.69
ENSGACG00000010980	-	0	1	0	0	26.86	5.89	4.14	4.96
ENSGACG00000010982	*AMN*	1	0	0	0	100.09	15.90	14.28	14.21
ENSGACG00000010985	*SLC2A2*	0	1	0	0	40.19	12.81	5.58	13.70
ENSGACG00000010987	*HID1 (1 of 2)*	0	1	1	0	4.89	7.32	63.63	62.95
ENSGACG00000010995	-	0	1	0	0	95.43	145.64	119.81	159.18
ENSGACG00000011003	*KIF20B (1 of 2)*	0	0	1	0	1.50	2.52	0.45	0.35
ENSGACG00000011004	*USH1C*	0	1	0	0	20.66	6.84	3.57	10.50
ENSGACG00000011014	*AAK1 (1 of 2)*	0	1	0	0	6.90	7.82	15.33	14.65
ENSGACG00000011018	*ALDOC*	0	1	0	0	0.22	1.17	1.40	0.55
ENSGACG00000011029	*LEPRE1*	0	1	0	0	1.54	1.73	4.83	2.29
ENSGACG00000011031	*RSBN1*	0	1	1	0	3.05	1.62	7.82	3.64
ENSGACG00000011040	*CALR*	0	1	0	0	9.25	12.99	16.91	20.11
ENSGACG00000011059	*LGALS3BP*	1	0	0	0	21.42	91.57	26.14	8.40
ENSGACG00000011062	*PTPRJ*	0	0	0	1	15.74	3.44	9.65	1.92

ENSGACG00000011067	*CANT1 (1 of 2)*	0	1	0	0	10.97	11.99	19.62	17.20
ENSGACG00000011072	*SYNGR2 (1 of 2)*	0	1	0	0	36.44	56.66	59.26	67.42
ENSGACG00000011081	*OSBPL5*	0	1	0	0	8.83	8.03	12.03	10.55
ENSGACG00000011099	*SLC22A15*	0	1	0	0	11.44	12.74	21.52	32.59
ENSGACG00000011100	*SPTB*	1	0	0	0	4.50	17.15	5.52	2.01
ENSGACG00000011107	*ACAD9*	0	1	0	0	36.44	36.05	43.98	41.08
ENSGACG00000011109	*NOTUM (2 of 2)*	0	1	0	0	2.45	0.86	0.13	0.72
ENSGACG00000011113	*IYD*	0	1	0	0	83.55	51.82	5.62	28.02
ENSGACG00000011115	*SEC63*	0	1	0	0	19.12	17.20	39.60	43.26
ENSGACG00000011136	*NBAS*	0	1	0	0	3.64	3.06	5.35	10.44
ENSGACG00000011143	-	0	1	0	0	11.03	5.77	1.92	2.07
ENSGACG00000011184	*TCF7L1*	0	1	0	0	6.91	8.15	20.27	21.68
ENSGACG00000011190	*RAB34*	0	1	0	0	24.90	14.36	3.61	7.25
ENSGACG00000011195	*TGOLN2*	0	1	0	0	22.00	22.75	28.09	29.66
ENSGACG00000011199	*RASD1 (2 of 2)*	0	1	1	1	0.01	0.10	10.24	0.00
ENSGACG00000011217	*SDF4*	0	1	0	0	43.63	55.21	77.34	87.70
ENSGACG00000011232	-	0	0	0	1	0.13	0.07	0.01	0.60
ENSGACG00000011258	*ALDH1L1*	0	1	0	0	101.60	37.38	9.97	42.52
ENSGACG00000011275	*PLEKHH1*	0	1	0	0	8.57	6.27	1.48	3.52
ENSGACG00000011308	*VPS33A*	0	1	0	0	13.23	20.05	16.29	10.48
ENSGACG00000011321	*FBXO2*	0	1	0	0	120.42	154.19	22.27	45.11
ENSGACG00000011330	*DIABLO (1 of 2)*	0	1	0	0	27.48	60.55	37.54	37.83
ENSGACG00000011336	-	0	1	0	0	23.22	3.97	3.47	5.80
ENSGACG00000011354	*LPL*	0	1	0	0	30.41	11.34	5.67	7.20
ENSGACG00000011391	*DNMT3A (1 of 2)*	0	0	0	1	30.35	18.43	7.45	43.25
ENSGACG00000011399	*C8orf42*	0	1	0	0	14.65	4.48	1.57	4.28
ENSGACG00000011419	*MAPRE3 (1 of 2)*	0	0	0	1	15.20	5.89	4.32	24.63
ENSGACG00000011422	*SLC22A7 (1 of 2)*	0	1	1	0	33.63	22.76	1.13	10.10
ENSGACG00000011430	*CD44*	0	1	0	0	53.68	70.15	88.45	55.57
ENSGACG00000011442	-	0	1	0	0	82.96	32.33	9.93	18.28
ENSGACG00000011450	-	0	0	1	0	9.22	15.39	1.62	1.80
ENSGACG00000011451	*TMC4*	0	1	1	0	16.38	8.96	19.06	11.99
ENSGACG00000011475	*CGB1*	0	1	1	0	0.22	0.05	5.40	2.57
ENSGACG00000011478	*FURIN (2 of 2)*	0	1	0	0	2.82	1.19	0.30	0.38
ENSGACG00000011484	*MAN2A2*	0	1	0	0	11.29	12.13	14.96	15.99
ENSGACG00000011497	*ARFRP1*	0	1	0	0	16.91	12.91	24.75	29.81
ENSGACG00000011503	*KDELR2 (1 of 2)*	0	1	0	0	15.77	34.98	28.18	31.34
ENSGACG00000011522	*CCDC167*	0	1	0	0	4.86	17.19	8.21	14.75
ENSGACG00000011524	-	0	1	1	0	40.58	59.50	6.70	13.99
ENSGACG00000011534	-	0	1	1	1	5.61	2.15	15.28	2.55
ENSGACG00000011552	-	0	1	0	0	11.84	16.64	2.22	4.14
ENSGACG00000011555	*NBEA (1 of 2)*	1	1	0	0	1.47	0.11	0.11	2.69
ENSGACG00000011556	*RORB*	0	1	0	0	2.47	0.50	0.14	0.57
ENSGACG00000011557	*CKS2*	0	0	1	0	32.98	196.83	22.96	14.65
ENSGACG00000011570	*CPAMD8*	0	1	0	0	5.52	2.31	0.65	1.79
ENSGACG00000011579	*DCLK1 (2 of 2)*	0	1	0	0	1.67	4.63	10.99	7.11
ENSGACG00000011587	-	0	1	0	0	10.46	13.78	1.82	4.00
ENSGACG00000011591	*FANCI*	0	1	0	0	6.47	4.73	1.08	1.65
ENSGACG00000011592	*STK19*	0	1	0	0	15.40	22.85	31.21	28.18
ENSGACG00000011595	-	0	1	1	0	11.20	15.75	2.02	3.73
ENSGACG00000011601	-	0	1	0	0	40.84	59.41	60.88	40.03
ENSGACG00000011612	*STXBP5 (2 of 2)*	0	1	1	0	13.99	10.61	19.23	29.79
ENSGACG00000011632	*CYTH3 (1 of 2)*	0	0	0	1	9.14	8.35	6.55	23.72
ENSGACG00000011641	*PKLR*	0	1	1	1	83.82	45.64	2.67	64.12
ENSGACG00000011642	*NCKAP5L*	0	1	0	0	4.52	2.31	0.69	1.29
ENSGACG00000011656	*EPM2A*	0	1	0	0	1.56	1.14	3.60	5.18
ENSGACG00000011668	*FREM2 (1 of 2)*	0	1	0	0	3.62	1.16	0.28	0.82
ENSGACG00000011689	*ARFIP2 (2 of 2)*	0	1	1	0	2.78	3.47	9.98	8.70
ENSGACG00000011691	*SLC11A2 (2 of 2)*	0	1	0	0	36.32	21.35	69.47	59.25
ENSGACG00000011698	*TP53I3*	0	1	0	0	10.97	44.06	21.80	17.24
ENSGACG00000011700	*ERN1*	0	1	0	0	18.42	40.15	110.96	190.77
ENSGACG00000011702	*ATF6*	0	1	0	0	20.54	43.37	74.22	97.54
ENSGACG00000011712	*NT5E*	0	1	0	1	10.76	16.04	17.81	6.27
ENSGACG00000011742	*XCR1 (2 of 2)*	0	0	1	0	4.09	7.51	0.83	0.56
ENSGACG00000011780	*TMEM30A (1 of 2)*	0	1	0	0	56.18	19.94	10.33	12.10
ENSGACG00000011782	*STAR*	1	0	0	0	0.41	2.78	0.53	0.13

ENSGACG00000011789	*RPRD2 (2 of 2)*	0	1	1	1	9.00	9.37	29.71	13.21
ENSGACG00000011791	*CDKN3*	0	0	0	1	2.99	9.79	5.65	0.65
ENSGACG00000011804	*USP16*	0	1	0	0	11.01	16.34	20.28	20.92
ENSGACG00000011812	*RWDD2B*	0	1	0	0	4.00	4.23	10.23	14.23
ENSGACG00000011813	*CRABP2 (2 of 2)*	0	0	0	1	4.15	24.78	7.42	1.82
ENSGACG00000011825	*EIF4E3 (1 of 2)*	0	0	1	0	29.95	14.55	31.33	53.91
ENSGACG00000011858	*HAGH*	0	1	0	0	40.79	49.26	48.38	53.81
ENSGACG00000011867	*NIN*	0	1	0	0	3.58	1.55	0.36	0.76
ENSGACG00000011879	-	0	1	0	0	22.89	7.24	3.25	7.56
ENSGACG00000011906	*AHCYL2 (2 of 2)*	0	1	0	0	40.82	3.37	0.09	2.55
ENSGACG00000011922	*COG6*	0	1	0	0	13.84	14.13	19.17	20.79
ENSGACG00000011934	-	0	1	0	0	97.75	151.32	281.39	366.45
ENSGACG00000011950	*RCBTB2*	0	1	0	0	14.89	18.06	36.78	39.58
ENSGACG00000011955	*RNF151*	0	0	0	1	3.25	5.95	3.80	0.99
ENSGACG00000011980	*C3orf52*	0	1	0	0	10.92	10.37	15.65	17.21
ENSGACG00000012002	*SLC50A1*	0	1	0	0	7.31	12.44	33.50	19.38
ENSGACG00000012007	*MST1R (1 of 2)*	0	1	0	0	50.48	38.13	109.84	257.79
ENSGACG00000012012	*CYP2W1 (3 of 5)*	0	1	1	0	231.81	67.13	0.31	42.58
ENSGACG00000012044	*TRAIP*	0	1	0	1	10.66	18.62	15.13	6.87
ENSGACG00000012055	*ACOT11 (2 of 2)*	0	1	0	0	7.05	1.88	0.74	2.40
ENSGACG00000012063	*ACTN1*	0	1	0	0	25.12	28.05	30.28	16.64
ENSGACG00000012066	*PRR5 (1 of 2)*	0	1	0	0	38.30	15.49	5.67	10.43
ENSGACG00000012073	-	0	1	0	0	23.64	4.72	2.41	0.65
ENSGACG00000012083	-	0	1	0	0	24.82	20.01	70.66	117.06
ENSGACG00000012086	*SLC17A5*	0	1	0	0	16.13	12.91	29.96	23.67
ENSGACG00000012088	-	0	1	0	0	41.53	35.53	115.94	202.75
ENSGACG00000012090	*COMT*	0	1	0	0	22.45	11.03	1.25	3.77
ENSGACG00000012099	*ERC1 (1 of 2)*	0	1	0	0	4.13	14.18	11.75	16.93
ENSGACG00000012103	*TECPR1 (2 of 2)*	0	1	0	0	6.21	2.31	1.02	2.87
ENSGACG00000012110	-	0	1	0	0	7.72	18.77	17.36	28.10
ENSGACG00000012130	*TTC9*	0	1	1	1	0.78	0.18	15.53	1.26
ENSGACG00000012148	-	0	0	1	0	14.62	7.28	17.48	13.18
ENSGACG00000012151	*KIAA1377*	0	0	1	0	15.70	7.16	17.03	13.53
ENSGACG00000012162	*PGR*	1	0	0	0	3.90	0.34	0.49	0.24
ENSGACG00000012174	-	0	1	0	0	387.27	31.48	0.77	2.60
ENSGACG00000012176	*CNTN5*	0	1	0	0	2.14	0.53	0.28	0.33
ENSGACG00000012177	*ANKRD6*	0	1	0	0	8.45	2.05	1.03	1.80
ENSGACG00000012178	-	0	1	0	0	141.36	49.05	1.58	1.45
ENSGACG00000012193	*GABRR1 (1 of 2)*	0	1	0	1	0.92	1.78	4.99	0.39
ENSGACG00000012197	*H6PD*	0	1	0	0	37.22	29.22	43.95	37.05
ENSGACG00000012220	*PIWIL1*	0	0	1	0	1.33	1.00	0.00	0.01
ENSGACG00000012223	*TRIP11*	0	1	1	0	10.17	6.40	12.70	11.57
ENSGACG00000012231	*ECM1*	0	0	0	1	38.60	32.96	27.52	12.97
ENSGACG00000012247	-	0	1	0	0	86.18	70.12	156.21	139.13
ENSGACG00000012251	-	0	1	0	0	21.45	18.65	46.83	42.52
ENSGACG00000012260	*TC2N*	0	1	0	0	8.74	10.86	15.34	13.06
ENSGACG00000012278	*CXCR5*	0	0	0	1	3.65	4.72	1.25	0.19
ENSGACG00000012289	*EDEM1*	0	1	1	0	6.30	5.57	13.45	15.86
ENSGACG00000012304	*SEPT9 (1 of 2)*	0	1	0	0	29.36	18.15	4.82	11.55
ENSGACG00000012306	*CPT1B*	1	1	0	1	8.89	0.60	0.65	3.67
ENSGACG00000012313	*ENPP5*	0	0	1	0	15.96	4.50	12.49	12.27
ENSGACG00000012317	*NCOA4*	0	1	0	0	74.60	75.31	89.12	100.04
ENSGACG00000012330	*RGL1*	0	1	1	0	17.02	10.17	29.38	20.66
ENSGACG00000012346	*ARG2*	0	0	0	1	21.34	16.39	6.91	2.41
ENSGACG00000012348	-	0	0	0	1	14.85	169.29	36.07	4.84
ENSGACG00000012358	-	0	1	0	0	9.46	5.31	1.18	2.48
ENSGACG00000012360	*DNAJC28*	0	0	0	1	11.62	5.77	7.26	2.18
ENSGACG00000012367	*FAM101A*	0	1	0	0	14.38	2.72	0.71	2.93
ENSGACG00000012396	*EDEM3*	0	1	1	0	18.76	19.06	65.80	46.83
ENSGACG00000012405	*EML4*	0	0	0	1	21.08	17.49	7.30	25.70
ENSGACG00000012410	*FGFR1 (1 of 2)*	1	1	0	0	9.45	1.76	0.76	2.10
ENSGACG00000012421	-	0	1	0	0	19.11	28.30	67.01	87.85
ENSGACG00000012427	*CKM (2 of 2)*	0	1	0	0	61.61	3.47	0.45	2.32
ENSGACG00000012449	*TNPO2 (1 of 2)*	0	0	0	1	10.02	11.75	4.66	20.76
ENSGACG00000012458	-	1	0	0	0	1.46	0.16	0.22	0.99
ENSGACG00000012462	-	0	0	1	0	3.08	1.46	4.60	6.95

ENSGACG00000012480	-	0	1	0	0	3.93	3.50	7.21	1.82
ENSGACG00000012487	*BMP2*	0	1	1	1	0.87	1.40	4.34	1.10
ENSGACG00000012494	*LIM2*	0	1	0	0	27.40	5.36	0.76	0.00
ENSGACG00000012496	*CRLF1 (2 of 2)*	0	1	1	1	0.54	0.08	3.04	0.02
ENSGACG00000012498	*MFSD4 (1 of 2)*	0	1	0	0	225.91	121.65	635.10	476.70
ENSGACG00000012500	*ARF4 (1 of 2)*	0	1	0	0	73.85	156.40	182.61	182.55
ENSGACG00000012502	*VSIG8*	0	1	0	1	11.23	2.11	0.33	4.34
ENSGACG00000012512	*LRRTM4 (3 of 3)*	0	1	0	0	1.35	0.09	0.02	0.12
ENSGACG00000012538	-	0	0	1	1	108.23	27.83	71.64	26.63
ENSGACG00000012547	-	0	1	0	0	111.93	234.64	195.82	199.92
ENSGACG00000012548	-	0	0	1	0	45.36	11.57	0.39	12.46
ENSGACG00000012551	-	0	1	0	0	216.23	451.93	333.88	375.15
ENSGACG00000012556	-	0	0	1	0	29.22	8.61	0.35	7.53
ENSGACG00000012592	-	1	0	0	1	1.97	0.30	0.36	1.54
ENSGACG00000012602	*CABP1 (2 of 2)*	0	0	0	1	23.02	2.09	0.72	17.40
ENSGACG00000012609	*LGALS1 (3 of 3)*	1	1	0	1	4.51	27.27	15.84	2.15
ENSGACG00000012618	*SCAP*	0	1	0	0	2.34	2.02	3.88	3.55
ENSGACG00000012631	*LGALS1 (2 of 3)*	1	1	0	1	11.39	57.10	32.10	4.97
ENSGACG00000012633	*MTHFR*	0	0	0	1	14.96	11.32	6.20	24.10
ENSGACG00000012637	*SGSM3*	0	1	0	0	11.16	10.62	17.81	17.17
ENSGACG00000012658	*ATP8B3*	0	1	0	0	11.31	31.88	144.21	131.26
ENSGACG00000012678	*EIF2S3*	0	1	0	0	64.45	107.69	72.27	93.62
ENSGACG00000012707	*ADPRHL2*	0	1	0	0	13.56	16.08	19.30	23.34
ENSGACG00000012713	*SLC7A13*	0	1	1	0	26.81	14.58	154.46	154.87
ENSGACG00000012741	*CACNA1A*	1	1	0	0	0.31	0.02	0.01	0.01
ENSGACG00000012745	*SAT1*	0	0	0	1	147.58	204.41	277.02	107.44
ENSGACG00000012779	-	0	1	0	0	36.15	48.08	5.60	3.75
ENSGACG00000012792	-	0	1	0	0	38.89	31.98	5.51	2.08
ENSGACG00000012794	-	0	0	1	0	6.16	24.85	1.44	0.63
ENSGACG00000012795	*HHATL (1 of 2)*	0	1	0	0	5.07	3.52	0.64	0.31
ENSGACG00000012813	*CALU*	0	1	0	0	52.95	121.38	403.53	364.72
ENSGACG00000012817	*IDUA*	0	1	0	1	6.38	7.03	13.02	6.82
ENSGACG00000012821	-	0	1	0	0	12.84	3.75	0.87	3.29
ENSGACG00000012826	-	0	1	1	0	0.24	1.02	11.02	1.67
ENSGACG00000012832	-	0	1	1	0	0.13	0.75	7.33	1.22
ENSGACG00000012843	*WDR43*	0	0	0	1	27.21	29.67	14.09	44.58
ENSGACG00000012855	*NME4*	0	1	0	0	17.70	3.03	1.68	6.50
ENSGACG00000012874	*PPP6R2 (2 of 2)*	0	1	0	0	9.33	18.56	21.58	13.90
ENSGACG00000012888	*HSP90AA1 (1 of 2)*	0	0	1	0	10.80	17.89	2.66	1.50
ENSGACG00000012900	*MIOX*	0	1	1	0	120.77	188.12	0.11	2.75
ENSGACG00000012923	*SLC25A47*	0	1	0	0	17.29	5.34	2.06	3.72
ENSGACG00000012926	*SCFD1*	0	1	0	0	28.79	42.67	56.92	50.28
ENSGACG00000012958	*NR1D2 (2 of 2)*	0	1	0	0	41.99	17.86	3.81	12.52
ENSGACG00000012972	*GORASP2 (1 of 2)*	0	1	0	0	16.84	21.14	26.12	33.81
ENSGACG00000012997	*PLCH2 (1 of 2)*	0	0	1	0	1.74	0.41	1.29	2.73
ENSGACG00000013002	-	0	1	0	0	31.13	5.91	2.10	0.95
ENSGACG00000013018	*PRKCZ*	0	1	0	1	3.88	0.92	0.23	1.80
ENSGACG00000013022	*MOCS1*	0	1	0	0	9.88	5.96	1.62	3.39
ENSGACG00000013028	*NME3*	0	1	0	0	2.09	9.47	10.17	7.38
ENSGACG00000013064	*PTPRQ*	1	1	0	0	37.84	3.49	1.48	7.21
ENSGACG00000013082	*IGFBP1 (2 of 2)*	0	1	0	0	3.07	0.38	0.05	1.44
ENSGACG00000013087	*SEL1L*	0	1	1	0	19.09	17.39	48.01	46.18
ENSGACG00000013107	-	0	1	0	0	7.61	4.15	1.28	1.60
ENSGACG00000013135	*VWF*	0	1	0	0	12.03	4.34	1.53	1.69
ENSGACG00000013149	*ANO2 (2 of 2)*	0	1	1	0	0.87	1.71	3.64	3.95
ENSGACG00000013175	-	1	1	0	0	26.50	3.57	1.10	2.79
ENSGACG00000013177	*TTC23*	0	1	0	0	13.52	3.16	0.74	1.75
ENSGACG00000013183	*AQP4*	0	0	0	1	1.62	0.39	0.75	0.05
ENSGACG00000013184	*IL20RA*	0	1	0	1	45.86	15.70	7.36	30.86
ENSGACG00000013244	-	0	1	0	0	75.46	88.59	12.90	11.37
ENSGACG00000013257	*PAK1*	0	1	0	0	3.61	4.67	12.66	14.75
ENSGACG00000013269	*MYO7A (1 of 2)*	0	1	0	0	6.33	1.56	0.79	1.97
ENSGACG00000013274	*ADI1*	0	1	0	0	27.97	46.79	62.17	66.34
ENSGACG00000013300	*LAMA3*	0	0	0	1	2.88	1.44	1.20	4.81
ENSGACG00000013313	*PGM3*	0	1	0	0	6.35	12.59	17.01	18.77
ENSGACG00000013315	*SLC35F2 (2 of 2)*	1	1	0	0	5.83	0.63	0.08	0.10

ENSGACG00000013326	-	0	1	0	1	10.82	39.67	33.54	2.69
ENSGACG00000013330	*TSPAN13*	0	1	0	0	70.35	64.40	247.16	166.78
ENSGACG00000013417	*BCR (2 of 2)*	0	0	0	1	26.41	12.60	8.96	28.83
ENSGACG00000013438	-	0	0	0	1	78.18	63.38	37.27	19.61
ENSGACG00000013445	*LMBRD2 (1 of 2)*	0	1	0	0	12.11	12.85	22.49	15.16
ENSGACG00000013448	-	0	1	0	0	22.22	26.95	35.92	27.81
ENSGACG00000013449	-	0	1	0	0	19.08	6.17	1.08	6.78
ENSGACG00000013458	*AIDA*	0	0	0	1	10.00	11.09	5.24	25.75
ENSGACG00000013463	*RAD9B*	0	1	0	0	0.26	0.73	4.29	1.79
ENSGACG00000013469	*PPTC7 (1 of 2)*	0	1	0	0	13.56	14.84	23.90	14.89
ENSGACG00000013473	*BTBD11 (1 of 2)*	0	1	0	0	2.57	0.48	0.23	0.59
ENSGACG00000013483	-	0	1	0	0	35.28	49.91	155.08	133.96
ENSGACG00000013498	*TCTE1*	0	1	0	0	2.62	3.08	6.43	6.30
ENSGACG00000013532	*SLC30A7*	0	1	0	0	20.44	32.39	62.28	56.08
ENSGACG00000013535	*DPH5*	0	1	0	0	15.81	23.24	22.71	40.25
ENSGACG00000013543	*CCDC64 (1 of 2)*	0	1	0	0	2.43	0.51	0.22	0.55
ENSGACG00000013545	-	0	1	1	0	6.57	4.99	0.06	2.28
ENSGACG00000013555	*CORO1A*	0	1	0	0	288.23	498.78	62.69	49.20
ENSGACG00000013567	*TXLNB (2 of 2)*	0	1	0	0	3.87	0.10	0.01	0.05
ENSGACG00000013581	*DHDDS*	0	1	0	0	8.48	14.08	20.90	25.03
ENSGACG00000013583	*COX6A2*	0	1	0	0	22.50	19.23	1.12	11.87
ENSGACG00000013621	-	0	1	0	0	107.42	26.69	5.62	14.90
ENSGACG00000013624	*TMEM251*	0	1	0	1	53.92	95.01	99.13	41.93
ENSGACG00000013626	*CDC20*	0	0	1	0	12.41	12.85	1.30	0.75
ENSGACG00000013627	*ASB2*	0	1	1	0	9.69	11.48	52.87	39.42
ENSGACG00000013630	*FAM180A (1 of 2)*	0	1	0	0	16.15	15.64	37.91	58.83
ENSGACG00000013631	-	0	1	0	0	13.64	15.71	30.31	17.48
ENSGACG00000013637	*PPP4R4*	0	1	1	0	4.15	2.79	16.08	9.05
ENSGACG00000013642	*OGFOD2*	0	1	0	0	6.28	6.11	10.49	4.81
ENSGACG00000013645	*DPT*	0	1	0	0	4.80	16.01	23.53	11.91
ENSGACG00000013666	*AGXT (2 of 2)*	0	1	0	0	1142.37	605.93	96.79	652.87
ENSGACG00000013689	*PIPOX*	0	1	1	0	110.53	46.14	3.71	25.52
ENSGACG00000013708	*KIF1A (1 of 2)*	0	1	0	1	6.25	1.12	0.29	2.85
ENSGACG00000013715	*GLDC*	0	1	0	0	47.83	11.11	8.04	19.59
ENSGACG00000013724	*GALNT8 (2 of 2)*	0	1	1	0	0.50	0.19	12.95	8.38
ENSGACG00000013729	*CNGA3*	0	0	0	1	2.22	1.02	6.77	0.14
ENSGACG00000013732	*AMOTL2 (1 of 2)*	0	1	0	0	22.21	7.43	3.94	6.12
ENSGACG00000013739	-	0	1	0	0	0.06	1.07	1.09	0.56
ENSGACG00000013746	*NOP58*	0	0	0	1	43.22	88.66	27.29	101.89
ENSGACG00000013760	-	0	0	0	1	6.87	6.16	8.59	3.70
ENSGACG00000013763	*DYRK4*	0	1	0	0	19.43	7.26	3.08	6.38
ENSGACG00000013768	*WFDC1*	0	0	0	1	8.96	7.08	1.50	7.41
ENSGACG00000013783	*EMB*	0	1	1	1	0.80	0.81	28.39	3.68
ENSGACG00000013800	*EIF2B3*	0	1	0	0	16.44	22.11	23.09	17.23
ENSGACG00000013802	*GRIK3*	0	1	0	1	0.11	0.49	0.52	0.04
ENSGACG00000013805	*C12orf5 (2 of 2)*	0	0	1	0	2.44	0.69	3.78	3.48
ENSGACG00000013811	*ICA1L*	0	1	0	0	9.51	10.77	18.33	22.07
ENSGACG00000013828	*PRKCSH*	0	1	0	0	29.15	41.21	65.31	82.77
ENSGACG00000013840	*ARL14 (2 of 2)*	0	1	0	0	22.83	9.65	3.20	10.79
ENSGACG00000013844	*PLCXD1 (1 of 3)*	0	1	1	0	0.85	0.47	3.60	2.19
ENSGACG00000013890	-	0	1	0	0	30.50	9.20	2.19	9.45
ENSGACG00000013898	-	0	1	0	0	61.12	16.81	4.25	6.36
ENSGACG00000013906	*PLLP*	0	1	0	0	32.73	38.47	48.08	16.74
ENSGACG00000013933	*XIRP2 (1 of 2)*	0	1	0	0	7.37	2.84	1.10	4.51
ENSGACG00000013941	*DHRS9*	0	1	0	0	48.08	14.83	5.21	6.65
ENSGACG00000013944	*GPC1 (1 of 2)*	0	1	1	0	1.14	1.04	3.52	5.31
ENSGACG00000013951	*EPHB1 (1 of 2)*	0	0	0	1	3.57	1.16	0.61	2.56
ENSGACG00000013963	*B3GNT9*	1	1	0	0	0.67	12.12	15.48	16.22
ENSGACG00000013968	*CTPS1 (2 of 2)*	0	1	0	1	27.27	10.90	4.82	19.35
ENSGACG00000013974	*MTHFS*	0	1	0	0	36.34	37.06	60.65	68.89
ENSGACG00000013981	-	0	1	0	0	35.85	24.20	7.75	11.27
ENSGACG00000013988	-	0	1	1	0	9.93	10.33	33.51	23.22
ENSGACG00000013989	-	0	1	0	0	48.14	27.14	5.55	12.51
ENSGACG00000013999	-	0	1	0	0	110.99	40.56	23.45	36.31
ENSGACG00000014008	-	0	0	1	0	815.41	186.16	820.37	255.69
ENSGACG00000014033	*UBFD1*	0	1	0	0	14.28	23.25	20.88	26.74

ENSGACG00000014044	*PREB*	0	1	0	0	25.12	26.72	36.36	45.43
ENSGACG00000014057	*ZFAND2A*	0	1	1	1	20.65	24.30	68.00	14.28
ENSGACG00000014068	*CHTF8*	0	1	0	0	21.84	22.22	2.89	22.03
ENSGACG00000014075	-	0	1	0	0	5.82	0.99	0.53	2.38
ENSGACG00000014089	*VIMP*	0	1	0	0	48.36	66.31	108.41	114.54
ENSGACG00000014106	*RNFT2*	0	1	1	0	3.16	2.11	6.09	2.71
ENSGACG00000014119	*ACP2*	0	1	0	0	21.05	24.62	32.29	36.02
ENSGACG00000014177	*GOSR2*	0	1	0	0	32.18	59.34	97.71	84.12
ENSGACG00000014184	*SSR1 (8 of 12)*	0	1	0	0	6.15	10.56	17.69	16.55
ENSGACG00000014196	-	0	1	0	1	0.72	0.98	3.36	0.54
ENSGACG00000014197	*DCUN1D4*	0	0	1	0	30.60	15.32	37.08	105.73
ENSGACG00000014248	*N6AMT1*	0	1	0	0	9.48	14.96	13.23	19.67
ENSGACG00000014256	*JAGN1 (2 of 3)*	0	1	1	0	12.90	24.27	51.90	42.83
ENSGACG00000014260	*HSPB8*	0	1	0	0	12.75	13.52	38.65	60.64
ENSGACG00000014266	*XBP1*	0	1	0	0	64.83	209.33	806.41	512.96
ENSGACG00000014283	-	0	0	0	1	1.36	0.49	0.80	8.46
ENSGACG00000014373	*COL8A1 (1 of 2)*	0	1	1	1	0.90	0.88	4.31	1.24
ENSGACG00000014376	*JAGN1 (1 of 3)*	0	1	0	0	16.26	31.03	69.92	45.67
ENSGACG00000014377	*UROD*	1	0	0	0	20.12	100.01	22.52	20.09
ENSGACG00000014413	*TTPA*	0	0	0	1	48.99	50.80	11.88	5.01
ENSGACG00000014423	*CNPY1*	0	1	0	0	18.71	48.14	272.25	119.58
ENSGACG00000014445	*UAP1*	0	1	0	0	18.88	30.01	32.62	41.76
ENSGACG00000014453	*DAD1*	0	1	0	0	83.61	384.34	571.05	408.84
ENSGACG00000014465	-	1	0	0	0	1.93	9.72	2.04	0.54
ENSGACG00000014468	*FADS6*	0	1	0	0	22.61	16.58	3.76	4.58
ENSGACG00000014480	*HID1 (2 of 2)*	0	1	1	0	8.07	14.63	48.83	35.70
ENSGACG00000014487	*HBM*	0	1	0	1	23.89	102.12	57.74	17.26
ENSGACG00000014505	-	0	1	1	0	79.02	81.22	2.53	55.00
ENSGACG00000014520	*KANK1*	0	1	0	0	12.41	4.51	1.74	3.08
ENSGACG00000014535	*CYP2J2 (3 of 6)*	0	1	0	0	10.72	4.36	0.67	1.22
ENSGACG00000014541	*DOK4*	0	1	1	1	2.59	0.87	9.10	0.83
ENSGACG00000014556	-	0	0	0	1	5.42	5.09	4.60	1.00
ENSGACG00000014571	*CTSL1*	0	0	1	0	126.58	52.75	177.47	113.94
ENSGACG00000014589	-	1	0	0	0	2.62	0.03	0.08	0.40
ENSGACG00000014605	*U2AF1*	0	1	0	1	26.66	51.91	66.33	30.86
ENSGACG00000014613	*PRDX6 (2 of 2)*	0	1	0	0	46.32	100.69	59.38	55.78
ENSGACG00000014627	*CBS*	0	1	0	0	59.72	96.26	387.93	351.40
ENSGACG00000014646	-	1	1	0	0	21.75	199.21	151.24	293.22
ENSGACG00000014662	-	0	1	0	0	7.92	20.15	19.42	11.67
ENSGACG00000014663	*BEST3*	0	1	1	0	3.76	3.21	172.02	28.51
ENSGACG00000014665	*CTSE*	0	1	1	0	0.31	0.23	20.90	2.94
ENSGACG00000014669	*CYP4F2*	0	0	0	1	116.70	144.07	42.90	10.55
ENSGACG00000014674	*CYP1A2*	1	1	0	0	32.57	5.55	2.73	20.30
ENSGACG00000014690	-	0	1	0	1	0.07	0.20	1.39	0.06
ENSGACG00000014696	*SEMA7A*	0	0	0	1	57.55	78.22	13.81	7.33
ENSGACG00000014705	*METTL13*	0	0	0	1	8.43	7.05	2.12	11.52
ENSGACG00000014708	*C12orf28 (2 of 2)*	0	1	0	0	0.11	0.28	1.43	1.68
ENSGACG00000014710	*C12orf28 (1 of 2)*	0	1	0	0	0.22	0.59	1.70	2.01
ENSGACG00000014740	*UTP18*	0	1	0	0	24.46	38.36	28.14	48.81
ENSGACG00000014752	-	0	1	0	0	20.57	8.26	0.09	0.30
ENSGACG00000014756	*F3 (2 of 2)*	0	1	0	0	8.18	15.61	79.65	14.65
ENSGACG00000014764	*SV2B*	0	1	0	0	1.89	2.33	4.87	12.50
ENSGACG00000014790	*CACNB4 (1 of 2)*	0	1	1	1	0.06	1.27	16.10	0.13
ENSGACG00000014802	*RDM1*	0	0	0	1	8.43	9.10	3.44	15.71
ENSGACG00000014819	-	0	1	1	0	0.86	0.75	17.77	15.44
ENSGACG00000014827	*UNC50*	0	1	0	0	21.53	18.82	25.21	30.35
ENSGACG00000014838	*PTK2B (2 of 2)*	0	0	0	1	18.05	24.92	5.91	3.01
ENSGACG00000014841	-	0	0	1	0	0.29	0.01	1.03	0.11
ENSGACG00000014856	*MEF2A*	0	1	1	0	1.47	1.95	6.13	1.95
ENSGACG00000014866	*JMJD8*	0	1	0	0	6.26	10.90	16.08	20.91
ENSGACG00000014870	*C1orf27*	0	1	0	0	8.78	11.83	13.36	33.24
ENSGACG00000014875	*DPYD*	0	1	0	0	59.96	25.95	9.52	45.77
ENSGACG00000014889	*MCF2L (2 of 2)*	0	1	1	0	5.00	2.76	10.70	7.47
ENSGACG00000014896	*NCKAP5*	0	0	0	1	8.98	4.56	1.89	8.16
ENSGACG00000014905	*RAB2A*	0	1	0	0	54.80	59.67	86.67	98.09
ENSGACG00000014929	*IMPAD1*	0	1	0	0	10.89	13.95	19.12	29.40

ENSGACG00000014936	-	1	0	0	0	5.49	233.88	19.64	6.29
ENSGACG00000014945	*ATP7B*	0	1	0	0	4.44	0.65	0.36	0.50
ENSGACG00000014947	*FITM1 (1 of 2)*	0	0	0	1	0.10	0.00	0.00	1.50
ENSGACG00000014973	*NAT2*	0	1	0	0	6.48	36.55	27.71	14.56
ENSGACG00000014981	*SCG3*	0	1	0	0	0.38	1.90	16.60	6.65
ENSGACG00000015005	*NUPR1*	0	1	0	0	308.32	620.90	2144.34	1043.48
ENSGACG00000015014	*WRN*	0	1	1	1	7.04	4.21	20.89	10.45
ENSGACG00000015057	-	0	1	0	1	1.06	0.10	0.01	0.39
ENSGACG00000015179	*ITGB6*	0	1	0	0	1.06	0.92	3.64	3.86
ENSGACG00000015202	*KDELR2 (2 of 2)*	0	1	0	0	33.87	48.27	152.39	152.27
ENSGACG00000015247	-	0	1	0	0	841.43	2297.74	2465.26	3354.64
ENSGACG00000015262	*PCDH20*	0	1	0	0	1.46	0.02	0.02	0.09
ENSGACG00000015278	-	0	1	0	0	26.74	29.35	31.77	39.19
ENSGACG00000015285	*ARL6IP6*	0	1	0	0	10.45	12.61	41.86	28.85
ENSGACG00000015301	*UNC13A*	0	0	1	0	3.88	1.67	4.14	2.29
ENSGACG00000015303	*DDRGK1*	0	1	0	0	32.14	37.64	44.93	30.55
ENSGACG00000015312	*ATP1B1 (2 of 2)*	0	1	0	0	775.95	439.86	156.11	350.89
ENSGACG00000015323	*DDX4*	0	0	1	0	11.91	2.85	0.03	0.01
ENSGACG00000015329	*MTR*	0	0	0	1	7.78	5.95	5.83	26.86
ENSGACG00000015331	*TMCO3*	0	0	1	0	5.94	2.51	6.55	5.18
ENSGACG00000015354	*PTPRS (1 of 2)*	0	1	1	0	1.80	0.73	2.47	0.92
ENSGACG00000015362	*JMJD1C*	0	1	0	0	11.64	9.05	16.65	26.76
ENSGACG00000015367	*CDK5R2 (2 of 2)*	0	1	0	0	10.28	4.40	1.14	2.09
ENSGACG00000015371	*FMNL2 (2 of 2)*	0	1	0	0	2.71	0.83	0.31	0.76
ENSGACG00000015375	-	1	1	0	0	41.78	4.29	2.95	10.46
ENSGACG00000015411	-	0	1	0	0	204.13	121.12	33.39	107.51
ENSGACG00000015415	-	0	1	0	1	44.40	40.46	4.13	33.07
ENSGACG00000015419	*CMTM4*	0	1	0	0	28.40	20.94	36.54	37.32
ENSGACG00000015420	*IMPG2 (1 of 2)*	1	1	0	0	2.21	0.25	0.01	0.23
ENSGACG00000015423	*CKLF-CMTM1*	0	0	0	1	33.89	95.68	15.25	5.65
ENSGACG00000015455	*COPB2*	0	1	0	0	26.97	35.21	34.90	38.00
ENSGACG00000015458	*MAN2A1*	0	1	1	0	13.58	13.07	34.63	21.28
ENSGACG00000015490	*UFC1*	0	1	0	0	5.21	20.67	14.51	18.67
ENSGACG00000015493	*FKTN*	0	1	0	0	21.87	36.40	28.25	25.54
ENSGACG00000015496	*CERS4 (2 of 2)*	0	1	0	0	31.76	9.47	4.42	10.44
ENSGACG00000015513	*RAB3A (1 of 2)*	0	1	1	0	7.81	5.86	20.17	6.45
ENSGACG00000015516	*EPHX2*	0	1	0	0	20.26	14.49	1.82	7.98
ENSGACG00000015518	*FGFR1 (2 of 2)*	0	1	0	0	28.27	8.41	4.65	7.55
ENSGACG00000015528	-	0	0	0	1	13.68	42.61	4.86	1.18
ENSGACG00000015537	*CYBB*	0	0	0	1	212.37	380.76	69.88	21.08
ENSGACG00000015557	-	1	0	1	0	0.68	28.74	0.41	0.15
ENSGACG00000015561	*ANGPTL4 (1 of 2)*	0	0	0	1	5.66	1.52	1.34	12.22
ENSGACG00000015566	*CASC4*	0	1	1	0	40.45	41.69	96.46	56.76
ENSGACG00000015582	-	0	1	0	0	3.08	4.51	12.71	13.25
ENSGACG00000015584	*TMED2*	0	1	0	0	74.72	132.67	272.45	238.22
ENSGACG00000015589	*STAT1*	1	0	0	0	39.99	143.02	44.68	58.47
ENSGACG00000015610	*MFSD6*	0	1	1	0	6.85	6.22	19.52	11.06
ENSGACG00000015626	*NUCB2 (1 of 2)*	0	1	0	0	13.31	19.32	23.73	26.39
ENSGACG00000015629	*KYNU*	0	1	0	0	31.47	10.38	1.50	7.52
ENSGACG00000015632	*PLEKHA7 (1 of 2)*	0	0	1	0	7.54	3.09	5.70	3.62
ENSGACG00000015643	*COPB1*	0	1	0	0	29.55	48.52	55.49	63.49
ENSGACG00000015666	*FAM69A (2 of 2)*	0	1	0	0	7.73	8.47	24.97	36.91
ENSGACG00000015670	*RPL5 (3 of 3)*	0	1	0	0	86.28	201.77	147.71	152.38
ENSGACG00000015683	*SBF2*	0	1	0	0	11.63	7.59	13.57	11.89
ENSGACG00000015718	-	0	1	0	0	25.85	52.22	45.80	55.21
ENSGACG00000015726	*DKK3 (1 of 2)*	0	1	1	1	9.32	3.20	17.32	6.40
ENSGACG00000015734	*VAT1L*	0	1	0	0	4.31	0.76	0.33	1.21
ENSGACG00000015763	-	0	1	0	0	2.58	2.93	0.08	0.20
ENSGACG00000015767	*PROC*	0	1	0	0	38.90	11.14	1.99	4.26
ENSGACG00000015772	*AP3B1 (2 of 2)*	0	1	0	0	10.78	10.86	13.46	14.21
ENSGACG00000015777	*DMGDH*	0	1	0	0	4.88	0.76	0.28	0.67
ENSGACG00000015791	*PHLPP2*	0	0	0	1	9.91	3.36	2.90	10.39
ENSGACG00000015812	*KCNQ3*	1	1	0	0	2.48	0.31	0.06	0.29
ENSGACG00000015824	*MAST4*	0	1	1	0	1.89	1.68	19.91	11.27
ENSGACG00000015936	-	0	0	0	1	15.47	42.99	1.92	15.74
ENSGACG00000015943	*CYBA*	0	0	0	1	178.56	362.39	53.47	19.60

ENSGACG00000015948	*TCF25*	0	1	0	0	21.06	20.84	25.10	22.04
ENSGACG00000015976	*CENPN*	0	0	1	0	8.19	16.14	1.85	2.51
ENSGACG00000015983	*SURF1*	0	1	0	0	11.19	17.84	21.95	15.38
ENSGACG00000015994	*SLC35A3 (2 of 2)*	0	1	1	0	5.29	12.67	83.24	37.50
ENSGACG00000016001	*DEPDC7 (1 of 2)*	0	0	0	1	3.49	2.92	3.38	0.47
ENSGACG00000016020	*WT1 (1 of 2)*	0	1	0	0	15.95	10.26	2.54	5.01
ENSGACG00000016033	-	0	1	0	0	47.05	4.68	5.18	3.99
ENSGACG00000016042	-	0	0	1	0	1.42	8.12	0.30	0.51
ENSGACG00000016044	-	0	0	1	0	1.69	3.46	0.38	0.27
ENSGACG00000016070	-	0	0	0	1	353.92	572.45	123.04	53.73
ENSGACG00000016091	*PFN2 (2 of 2)*	0	1	1	0	20.04	14.75	54.05	43.92
ENSGACG00000016095	*AHSG*	0	1	1	0	0.44	0.30	2.77	2.88
ENSGACG00000016111	*DNA2*	0	1	0	0	8.90	4.97	1.23	2.56
ENSGACG00000016130	-	0	1	1	0	11.71	6.01	17.96	12.22
ENSGACG00000016132	*DDR2 (2 of 2)*	0	1	1	1	1.28	0.22	2.79	0.47
ENSGACG00000016136	*FAM114A1*	0	1	1	0	4.93	4.92	15.61	15.22
ENSGACG00000016142	*ATP5A1 (2 of 2)*	0	0	0	1	13.78	21.66	5.64	27.00
ENSGACG00000016143	*RHOU*	0	1	0	0	8.59	2.44	0.97	1.53
ENSGACG00000016173	*PRUNE2 (1 of 2)*	0	1	1	0	35.72	33.94	85.09	51.03
ENSGACG00000016175	*HORMAD1*	0	0	1	0	0.23	3.46	0.03	0.04
ENSGACG00000016178	*UGDH*	0	1	0	0	33.11	44.66	52.99	50.63
ENSGACG00000016183	-	0	1	1	0	127.58	63.19	1373.57	466.22
ENSGACG00000016184	*UBE2K (2 of 2)*	0	1	0	0	17.26	15.43	25.29	21.57
ENSGACG00000016198	*CGREF1*	0	1	0	0	1.84	2.82	4.11	4.44
ENSGACG00000016199	-	0	1	0	1	25.77	22.72	42.54	22.43
ENSGACG00000016219	*NSUN7*	0	1	0	0	8.97	3.80	1.25	3.77
ENSGACG00000016233	-	0	1	0	0	38.18	41.09	57.71	57.84
ENSGACG00000016253	*FZD3 (2 of 2)*	1	0	0	0	9.02	1.80	2.92	3.55
ENSGACG00000016258	-	0	0	1	0	2.01	0.42	1.14	1.49
ENSGACG00000016266	-	0	0	0	1	47.38	61.49	16.31	9.25
ENSGACG00000016288	*LPIN1 (2 of 2)*	1	0	0	0	5.17	0.49	4.05	1.02
ENSGACG00000016309	*SUCO*	0	1	1	0	15.30	14.12	54.34	43.54
ENSGACG00000016311	*NIPSNAP3A*	0	1	0	1	141.44	94.57	30.51	125.33
ENSGACG00000016322	*SORCS1 (1 of 2)*	0	0	0	1	2.34	4.05	1.58	9.17
ENSGACG00000016335	*SLC43A1 (2 of 2)*	0	1	0	0	2.21	3.33	9.89	11.44
ENSGACG00000016349	*GNPNAT1*	0	1	0	0	19.87	19.10	38.00	39.45
ENSGACG00000016359	*ESRP2*	0	1	0	0	2.32	3.40	6.19	8.84
ENSGACG00000016360	*PCDH1 (1 of 2)*	0	1	0	0	13.55	5.51	2.70	5.18
ENSGACG00000016363	*TMED9*	0	1	0	0	75.82	108.72	224.06	229.03
ENSGACG00000016376	*PTPN13*	0	1	0	0	23.53	12.96	42.99	43.91
ENSGACG00000016390	*TENM3 (1 of 2)*	0	1	0	1	0.87	0.93	1.68	0.54
ENSGACG00000016395	-	0	1	0	0	41.66	44.64	70.81	58.53
ENSGACG00000016427	*SPCS3*	0	1	0	0	97.79	219.73	238.36	168.59
ENSGACG00000016429	*LMAN1L*	0	1	0	0	10.20	21.16	33.68	34.63
ENSGACG00000016430	*SEC11A*	0	1	0	0	62.86	124.66	273.97	227.04
ENSGACG00000016431	*SPON2*	0	0	0	1	2.48	2.45	2.91	0.49
ENSGACG00000016446	*WDR76*	0	1	0	0	2.65	0.44	0.06	0.93
ENSGACG00000016460	-	0	1	1	0	2.10	0.82	69.46	23.43
ENSGACG00000016464	*CXCL14*	0	0	0	1	4.47	1.23	10.88	0.55
ENSGACG00000016467	*TMED3*	0	1	0	0	22.48	46.77	121.17	110.10
ENSGACG00000016475	*PMM2*	0	1	0	0	33.33	74.35	125.19	109.15
ENSGACG00000016476	*IDH2 (1 of 2)*	0	1	0	0	2.93	3.21	6.78	10.16
ENSGACG00000016478	*AGXT2 (2 of 2)*	0	1	0	0	163.39	46.55	3.23	40.12
ENSGACG00000016491	*BRIX1*	0	1	0	0	29.76	54.16	54.11	54.70
ENSGACG00000016493	*WDR17*	0	1	0	1	6.49	3.85	0.67	6.10
ENSGACG00000016503	*DYM*	0	1	0	0	16.92	12.57	25.80	20.78
ENSGACG00000016514	*CTIF*	0	1	1	0	4.86	2.96	11.43	9.76
ENSGACG00000016517	*S1PR3*	0	1	1	1	1.55	0.91	4.56	0.29
ENSGACG00000016526	*PLK1*	0	0	1	1	14.39	27.76	3.19	0.99
ENSGACG00000016527	*UNC13C*	1	1	1	0	2.16	0.34	0.00	0.05
ENSGACG00000016534	*IGDCC4*	0	0	0	1	4.56	1.87	1.49	7.97
ENSGACG00000016550	*PDGFC*	0	1	0	0	4.84	10.75	21.57	56.92
ENSGACG00000016554	*EDF1*	0	1	0	0	136.99	189.14	246.82	238.34
ENSGACG00000016556	*GLRB (1 of 2)*	0	1	0	0	0.38	2.41	1.46	5.25
ENSGACG00000016582	*TXNDC12*	0	1	0	0	51.26	74.14	491.01	354.23
ENSGACG00000016586	*BTF3L4 (2 of 2)*	0	1	0	0	59.93	154.84	75.05	149.12

ENSGACG00000016612	*MPPE1*	0	1	0	0	4.87	0.78	0.23	0.28
ENSGACG00000016618	-	0	0	0	1	21.96	9.23	6.29	2.31
ENSGACG00000016630	*SLC12A4*	0	1	0	0	13.20	5.54	2.68	4.02
ENSGACG00000016632	*GRIN2A*	0	1	1	0	0.06	0.02	0.31	0.08
ENSGACG00000016633	*HSPA5*	0	1	1	0	93.15	125.83	578.73	720.25
ENSGACG00000016641	*QDPR*	0	1	0	1	43.48	44.42	4.40	35.51
ENSGACG00000016644	*GOLGA2*	0	1	0	0	12.43	11.68	16.32	22.94
ENSGACG00000016660	*SURF4*	0	1	0	0	36.14	44.67	163.66	199.90
ENSGACG00000016663	*RADIL*	0	1	0	0	2.99	0.73	0.16	0.50
ENSGACG00000016682	*IRX3*	0	1	0	0	6.24	7.69	9.73	9.75
ENSGACG00000016692	-	0	1	0	0	13.07	4.79	2.32	2.89
ENSGACG00000016749	*SRP72*	0	1	0	0	32.81	57.45	74.28	106.36
ENSGACG00000016753	*BRAT1*	0	1	0	0	5.02	2.20	0.60	2.37
ENSGACG00000016756	-	0	1	0	0	2.45	6.97	9.63	9.05
ENSGACG00000016765	*SLC12A1*	1	1	0	0	94.51	8.04	14.12	33.21
ENSGACG00000016797	*PDILT*	0	1	0	1	0.48	1.18	1.39	0.45
ENSGACG00000016800	*PDIA2*	0	0	0	1	1.82	1.31	1.02	5.83
ENSGACG00000016813	-	0	1	0	0	19.03	7.15	1.86	7.28
ENSGACG00000016821	*LMAN2*	0	1	0	0	26.85	32.50	60.50	50.90
ENSGACG00000016848	*G3BP1*	0	0	0	1	9.08	11.71	4.58	21.45
ENSGACG00000016856	*SLC25A48*	0	1	1	0	3.72	2.37	75.21	17.37
ENSGACG00000016860	*MFGE8 (1 of 2)*	0	1	1	1	0.96	0.42	4.94	0.92
ENSGACG00000016865	-	0	1	0	0	39.42	50.22	65.45	44.51
ENSGACG00000016875	*TMEM208*	0	1	0	0	17.91	32.79	36.77	44.27
ENSGACG00000016898	*CALR3 (2 of 2)*	0	1	0	0	150.15	302.65	312.83	445.74
ENSGACG00000016911	-	0	1	0	0	44.27	88.59	261.24	129.80
ENSGACG00000016928	*PRODH*	0	1	0	0	62.93	16.66	8.38	10.69
ENSGACG00000016961	*CLN6*	0	1	0	0	6.93	13.50	10.21	12.61
ENSGACG00000016980	*STX5*	0	1	0	0	35.24	42.36	65.31	57.52
ENSGACG00000016984	*MSMO1*	0	1	1	1	4.60	5.07	21.94	4.16
ENSGACG00000016985	*SEC62*	0	1	1	0	19.75	16.02	41.23	29.37
ENSGACG00000017003	*APOD (1 of 6)*	0	0	1	1	1.12	0.15	2.03	0.04
ENSGACG00000017040	*ATL3*	0	1	0	0	26.16	19.98	29.14	45.16
ENSGACG00000017041	-	0	1	0	0	145.35	44.15	17.20	24.82
ENSGACG00000017043	-	1	0	0	0	0.31	1.48	0.35	0.24
ENSGACG00000017044	*ANXA5 (2 of 2)*	0	1	0	1	122.13	186.59	207.33	42.58
ENSGACG00000017048	*GGT5 (2 of 2)*	0	1	0	0	27.72	16.37	4.24	8.19
ENSGACG00000017049	-	0	1	0	0	26.64	26.88	46.94	63.23
ENSGACG00000017051	*YIF1A*	0	1	0	0	26.63	31.92	68.91	55.61
ENSGACG00000017083	*SLC25A43*	0	1	0	0	78.07	24.83	5.98	8.65
ENSGACG00000017092	*HTATIP2*	0	1	0	0	19.31	19.21	29.29	40.43
ENSGACG00000017100	*LRP4*	1	1	0	0	8.33	1.36	0.64	2.11
ENSGACG00000017122	*TEX11*	1	0	1	0	0.03	1.39	0.02	0.03
ENSGACG00000017123	-	0	1	0	0	10.21	3.19	1.45	1.60
ENSGACG00000017132	-	0	1	0	0	14.96	13.49	34.47	65.35
ENSGACG00000017136	*TMEM154*	0	0	0	1	32.57	51.90	11.54	4.58
ENSGACG00000017137	*TTC17*	0	1	0	0	13.09	10.70	18.05	16.36
ENSGACG00000017140	*TCEA1*	0	1	0	0	15.64	24.89	21.26	29.91
ENSGACG00000017143	*PET112*	0	1	0	0	5.76	6.99	11.49	12.61
ENSGACG00000017144	*OSBP2 (2 of 2)*	0	0	0	1	1.29	1.53	1.06	4.54
ENSGACG00000017148	*SLC35E4*	0	1	0	0	5.74	8.88	16.73	38.60
ENSGACG00000017163	*SFTPB (1 of 2)*	0	0	1	0	268.95	343.40	53.01	141.96
ENSGACG00000017193	*NR3C2*	0	1	0	0	4.22	0.79	0.36	0.61
ENSGACG00000017206	*PRMT10*	0	1	0	0	7.51	7.37	10.36	13.35
ENSGACG00000017214	*TMEM184C*	0	1	0	0	19.82	14.37	24.71	20.58
ENSGACG00000017231	*HHIP*	0	1	0	0	2.77	1.21	0.27	0.59
ENSGACG00000017252	-	0	0	0	1	10.10	3.23	3.31	11.52
ENSGACG00000017263	*GPR112 (2 of 2)*	0	1	1	0	0.35	0.85	12.88	2.92
ENSGACG00000017274	*ZNF330*	0	1	0	0	10.07	15.59	14.69	13.84
ENSGACG00000017280	*C1QL3*	0	1	0	0	2.61	0.98	0.04	0.47
ENSGACG00000017283	*UCP1*	0	1	0	0	40.41	9.69	1.01	8.68
ENSGACG00000017285	*RAI14*	0	1	1	0	11.37	7.72	16.73	14.70
ENSGACG00000017307	*DAPK2 (1 of 2)*	0	0	1	0	2.55	1.51	3.56	1.63
ENSGACG00000017323	*GREB1L*	0	1	0	0	6.77	21.18	59.71	53.81
ENSGACG00000017332	-	0	0	0	1	47.83	72.33	26.16	9.14
ENSGACG00000017336	*USP14*	0	1	0	0	31.54	42.66	42.85	70.54

ENSGACG00000017347	*USO1*	0	1	0	0	34.10	45.64	65.14	68.43
ENSGACG00000017362	*XPNPEP2*	1	0	0	0	53.93	3.23	5.15	4.43
ENSGACG00000017373	-	1	1	0	0	10.00	39.87	15.85	15.94
ENSGACG00000017374	-	1	1	0	0	17.69	68.36	32.01	28.07
ENSGACG00000017390	*CNTLN*	0	1	1	0	3.66	2.67	8.56	8.67
ENSGACG00000017395	-	0	0	0	1	32.26	28.51	16.49	49.41
ENSGACG00000017396	*SARDH*	0	0	0	1	22.19	23.00	12.93	40.78
ENSGACG00000017409	*SLC27A4*	0	0	0	1	14.23	8.56	5.83	19.68
ENSGACG00000017427	*SNAP47*	0	0	1	0	6.33	2.70	6.24	4.53
ENSGACG00000017437	-	0	1	0	0	94.15	125.53	142.25	128.35
ENSGACG00000017450	*CCDC109B (1 of 2)*	0	1	0	0	76.65	109.47	159.56	216.67
ENSGACG00000017467	*PCDH19*	0	1	0	1	4.72	1.25	0.12	3.69
ENSGACG00000017485	*TIMM8A*	0	1	0	0	31.95	57.36	41.14	63.73
ENSGACG00000017487	*CCNB2 (2 of 2)*	0	0	1	0	22.36	12.03	1.56	0.75
ENSGACG00000017494	*PAPSS1*	0	1	0	0	12.81	23.83	15.77	16.95
ENSGACG00000017498	*KCNG4 (2 of 3)*	0	1	0	0	5.51	1.99	0.29	0.30
ENSGACG00000017499	*NPDC1*	0	1	1	1	8.29	9.04	41.76	13.36
ENSGACG00000017501	*AIMP1 (1 of 2)*	0	1	0	0	39.17	64.70	47.70	48.92
ENSGACG00000017512	*TPPP3*	0	1	0	0	13.82	6.95	1.53	5.29
ENSGACG00000017537	*BCR (1 of 2)*	0	1	0	0	4.40	1.25	0.46	1.19
ENSGACG00000017578	-	0	1	1	1	28.05	18.13	49.26	13.35
ENSGACG00000017583	*GANAB*	0	1	0	0	26.61	31.07	37.99	56.61
ENSGACG00000017594	-	0	0	0	1	9.33	14.22	3.83	11.66
ENSGACG00000017595	-	1	1	0	0	1.42	0.16	0.04	0.26
ENSGACG00000017596	*WBP1 (1 of 2)*	0	0	1	0	0.99	0.40	2.51	1.72
ENSGACG00000017601	-	0	1	0	0	47.32	40.57	64.66	64.37
ENSGACG00000017617	*GPN3*	0	0	0	1	11.39	22.44	6.85	25.67
ENSGACG00000017619	-	0	1	0	1	46.61	17.64	7.99	57.09
ENSGACG00000017637	-	0	1	1	1	1.20	1.58	10.95	4.17
ENSGACG00000017644	-	1	1	0	0	5.98	0.66	0.20	1.06
ENSGACG00000017650	-	0	1	0	0	4.42	17.25	31.37	50.12
ENSGACG00000017652	-	0	1	0	0	3.58	3.90	24.25	39.54
ENSGACG00000017654	-	0	1	0	0	2.33	2.16	18.05	32.98
ENSGACG00000017655	-	0	1	0	0	5.60	10.34	31.20	24.65
ENSGACG00000017666	*SLC16A2*	0	1	0	1	10.88	6.06	1.58	9.32
ENSGACG00000017671	*F8*	0	1	0	0	28.82	15.27	3.38	4.61
ENSGACG00000017681	-	0	0	0	1	17.52	12.30	5.87	0.07
ENSGACG00000017683	-	1	1	0	0	0.85	0.02	0.00	0.05
ENSGACG00000017696	*KIRREL (3 of 3)*	0	1	0	0	5.84	1.24	0.44	1.54
ENSGACG00000017701	-	0	0	0	1	84.48	180.29	49.75	0.60
ENSGACG00000017717	-	0	0	0	1	2.38	1.15	3.34	0.00
ENSGACG00000017739	*DUSP4*	0	1	1	0	4.99	4.12	10.72	6.06
ENSGACG00000017751	*COPA*	0	1	0	0	37.68	40.78	49.21	61.21
ENSGACG00000017761	*UFSP2*	0	1	0	0	10.28	17.25	27.34	30.99
ENSGACG00000017762	*PEX19*	0	1	0	0	26.42	24.03	45.18	36.13
ENSGACG00000017774	*RASGEF1B (2 of 2)*	0	1	0	0	22.03	9.23	2.55	7.17
ENSGACG00000017814	*SLC22A7 (2 of 2)*	0	1	0	0	613.63	359.60	100.76	401.83
ENSGACG00000017826	*DHX15 (1 of 2)*	0	1	0	0	26.48	32.96	31.47	41.42
ENSGACG00000017837	*SLC43A3 (2 of 2)*	0	1	1	0	16.83	10.79	31.01	24.92
ENSGACG00000017886	*VLDLR*	0	0	0	1	27.31	5.68	9.22	32.01
ENSGACG00000017905	-	0	0	1	0	0.14	0.02	0.26	0.08
ENSGACG00000017908	*C6*	0	1	0	0	3.70	2.67	0.45	0.10
ENSGACG00000017928	*ETF1*	0	1	0	0	31.81	50.13	42.48	69.20
ENSGACG00000017970	-	0	1	0	0	1.13	4.32	6.60	5.53
ENSGACG00000017973	*SLC30A5*	0	1	0	0	14.63	13.37	16.74	19.11
ENSGACG00000018002	-	0	1	0	0	24.63	31.12	4.96	10.04
ENSGACG00000018008	*MYO10 (1 of 2)*	0	1	0	0	22.22	7.00	2.98	9.23
ENSGACG00000018014	*CCNB1*	0	0	1	0	26.98	22.13	2.85	1.43
ENSGACG00000018036	*SLC34A1*	0	1	0	0	49.59	15.47	3.96	29.18
ENSGACG00000018045	-	0	0	0	1	0.10	0.07	0.01	0.77
ENSGACG00000018047	*CABP4 (1 of 2)*	0	1	1	0	3.57	2.24	22.49	12.27
ENSGACG00000018054	*SERPING1*	1	0	0	0	0.98	4.50	0.53	0.64
ENSGACG00000018062	*GFPT2*	0	1	0	0	30.98	36.26	146.52	110.33
ENSGACG00000018066	*GPR83*	0	0	0	1	0.03	0.00	0.01	1.71
ENSGACG00000018095	*SLC31A2*	0	1	0	0	43.99	49.13	214.61	153.51
ENSGACG00000018138	-	0	1	0	0	56.43	63.24	6.34	8.96

ENSGACG00000018150	*GSN*	0	0	0	1	20.66	24.57	17.90	8.39
ENSGACG00000018162	*BNIP3L (1 of 2)*	0	1	1	0	21.19	12.45	54.85	38.13
ENSGACG00000018166	*DPYSL2 (1 of 2)*	0	1	0	0	7.04	2.22	1.13	2.49
ENSGACG00000018185	*P4HB*	0	1	0	0	106.61	159.66	617.93	885.23
ENSGACG00000018207	-	0	1	1	1	3.89	5.94	23.96	5.09
ENSGACG00000018219	*CBX4 (1 of 2)*	0	1	0	1	2.32	4.24	7.40	0.70
ENSGACG00000018220	*CARD14*	0	0	1	0	31.55	10.49	33.19	27.60
ENSGACG00000018226	*PRF1 (2 of 5)*	0	0	1	0	2.00	4.80	0.30	2.33
ENSGACG00000018233	*PDCD11 (1 of 2)*	0	0	0	1	14.08	16.05	5.57	17.46
ENSGACG00000018236	*RASSF6*	0	1	0	1	36.23	23.16	5.09	89.57
ENSGACG00000018237	-	0	1	0	0	6.48	10.36	14.14	24.20
ENSGACG00000018249	-	0	1	0	0	29.59	33.41	1.64	8.83
ENSGACG00000018250	-	0	0	1	0	0.34	1.03	0.05	0.14
ENSGACG00000018253	-	0	1	1	0	9.24	9.55	1.25	2.32
ENSGACG00000018254	-	0	1	0	0	65.13	83.16	4.80	20.86
ENSGACG00000018282	*DNAJC25*	0	1	1	0	20.78	27.90	106.19	113.21
ENSGACG00000018287	*ZNF185*	0	0	0	1	5.90	3.82	0.75	4.59
ENSGACG00000018318	*DUSP1*	0	1	1	1	12.43	17.77	65.10	12.91
ENSGACG00000018319	*ERGIC1*	0	1	0	0	34.73	44.58	134.62	163.88
ENSGACG00000018323	-	0	1	0	0	6.10	1.20	0.47	1.54
ENSGACG00000018325	*PRELID1*	0	1	0	0	33.73	78.87	77.63	103.26
ENSGACG00000018349	*SLC27A6*	0	1	0	0	8.94	1.82	1.07	1.76
ENSGACG00000018363	*PRRC1*	0	1	0	0	16.08	18.02	27.30	31.55
ENSGACG00000018394	*DERL2*	0	1	0	0	20.86	28.41	56.54	53.39
ENSGACG00000018405	*C4orf33*	0	1	0	1	11.83	19.15	34.04	5.77
ENSGACG00000018416	-	0	0	0	1	51.35	71.04	27.07	9.56
ENSGACG00000018459	*SLC43A3 (1 of 2)*	0	1	0	0	0.07	0.17	0.86	0.36
ENSGACG00000018464	-	1	0	0	0	0.03	1.61	0.45	0.03
ENSGACG00000018467	*AFAP1L1 (2 of 2)*	0	1	0	0	4.54	1.94	0.53	1.57
ENSGACG00000018485	*ITK*	0	0	0	1	6.74	5.88	3.68	1.48
ENSGACG00000018491	-	0	1	1	0	109.96	49.62	1.98	14.96
ENSGACG00000018497	*AXIN2 (1 of 2)*	0	1	0	0	2.94	0.87	0.35	0.63
ENSGACG00000018523	*EFNB1*	0	1	0	0	12.35	5.30	1.92	4.69
ENSGACG00000018525	*AR (2 of 2)*	0	1	0	0	48.81	58.37	84.95	163.23
ENSGACG00000018527	*MSN (2 of 2)*	0	1	0	0	0.92	4.92	17.13	7.95
ENSGACG00000018531	*NTMT1*	0	1	0	0	14.76	22.51	21.69	37.85
ENSGACG00000018567	*GNAQ (3 of 3)*	0	1	0	0	11.37	5.63	1.62	2.62
ENSGACG00000018576	*TUSC3*	0	1	1	1	3.28	8.41	22.28	6.71
ENSGACG00000018606	*FBXW7*	0	0	0	1	3.05	3.06	1.79	7.20
ENSGACG00000018610	*EFHA2 (2 of 2)*	0	1	0	0	12.24	16.74	24.91	39.62
ENSGACG00000018611	*ABCE1*	0	1	0	0	23.03	53.06	38.26	46.62
ENSGACG00000018613	*FGF20*	0	0	1	0	3.70	1.96	5.20	4.86
ENSGACG00000018618	*SLC10A7*	0	1	0	0	20.59	21.12	40.08	34.02
ENSGACG00000018624	*SLC38A9*	0	1	0	0	15.83	11.74	18.58	18.13
ENSGACG00000018643	-	0	0	0	1	10.35	0.57	0.12	28.22
ENSGACG00000018671	-	0	0	1	0	11.70	1.41	8.19	0.99
ENSGACG00000018685	-	0	1	0	0	0.25	2.25	2.19	1.16
ENSGACG00000018719	-	0	1	0	0	62.65	42.77	125.59	85.63
ENSGACG00000018721	*XPNPEP1*	0	1	1	0	26.56	30.46	108.98	75.33
ENSGACG00000018723	*SYVN1*	0	1	0	0	13.93	19.02	46.41	42.35
ENSGACG00000018730	*COMMD5*	0	1	0	1	12.86	17.31	27.21	11.23
ENSGACG00000018731	*LONRF3*	0	1	0	0	4.16	7.08	33.96	44.88
ENSGACG00000018741	*GDE1 (1 of 2)*	0	1	0	0	10.39	13.58	18.18	11.31
ENSGACG00000018743	*PHKA1*	0	1	0	0	16.20	7.34	2.57	5.99
ENSGACG00000018748	-	0	1	0	0	1.74	0.45	0.12	0.70
ENSGACG00000018754	*ADCY9 (2 of 2)*	0	0	1	0	7.85	3.41	8.44	12.56
ENSGACG00000018755	*SSR1 (6 of 12)*	0	1	0	0	7.74	9.97	14.39	14.85
ENSGACG00000018771	*FAM125A*	0	1	0	0	15.39	22.10	27.08	12.85
ENSGACG00000018772	-	0	1	1	0	6.23	4.61	11.72	5.25
ENSGACG00000018774	*ACSL1 (2 of 2)*	0	1	0	0	19.91	25.47	74.50	109.80
ENSGACG00000018779	-	0	1	0	1	24.82	10.00	5.09	16.45
ENSGACG00000018791	*POLR1A*	0	0	0	1	7.30	9.38	3.80	12.44
ENSGACG00000018792	*TMED1 (1 of 2)*	0	1	0	0	35.21	44.91	72.49	77.99
ENSGACG00000018799	*PLS3 (2 of 2)*	0	1	0	1	23.86	10.39	3.24	12.20
ENSGACG00000018808	*EXOC6B*	0	1	1	0	11.85	9.02	23.08	21.67
ENSGACG00000018848	-	0	1	0	1	2.92	1.43	0.36	2.66

ENSGACG00000018865	*CNTN1 (2 of 2)*	0	1	0	0	1.29	0.25	0.06	0.29
ENSGACG00000018866	-	0	0	1	0	22.40	44.18	6.03	3.18
ENSGACG00000018868	*GCGR (2 of 2)*	0	1	0	0	6.30	1.00	0.51	0.42
ENSGACG00000018888	-	0	1	0	0	17.14	15.65	26.11	39.80
ENSGACG00000018905	-	0	0	1	0	2.39	13.35	0.90	0.95
ENSGACG00000018907	-	1	0	0	0	0.49	8.10	0.99	0.42
ENSGACG00000018926	*LRRN3*	0	1	1	0	4.08	4.23	20.65	30.30
ENSGACG00000018927	-	0	1	0	0	3.26	4.62	16.77	26.12
ENSGACG00000018928	-	0	0	0	1	19.26	36.73	15.91	6.92
ENSGACG00000018937	*C16orf88*	0	1	0	0	10.23	21.10	17.36	33.97
ENSGACG00000018952	*TSC2*	0	1	0	0	24.35	17.47	27.31	36.40
ENSGACG00000018953	*SLC37A3*	0	1	1	0	12.53	8.39	19.87	15.94
ENSGACG00000018958	*PPARA (2 of 2)*	0	1	0	0	36.07	14.97	5.86	4.21
ENSGACG00000018981	*KDM5A*	0	0	1	0	15.14	8.34	19.60	20.56
ENSGACG00000018995	*MRI1*	0	0	0	1	5.52	5.90	3.09	15.27
ENSGACG00000019002	-	0	1	0	0	2.82	3.50	8.01	10.03
ENSGACG00000019005	*CRELD2*	0	1	1	0	10.82	16.64	94.61	46.99
ENSGACG00000019033	-	0	0	0	1	7.67	4.67	2.03	7.59
ENSGACG00000019036	*SLC2A13 (1 of 2)*	0	0	1	0	0.20	0.10	1.57	0.32
ENSGACG00000019037	-	0	1	0	0	305.65	119.90	0.00	1387.54
ENSGACG00000019038	-	0	1	1	1	0.00	0.02	2.74	0.00
ENSGACG00000019040	*B3GNT1*	0	1	1	1	1.52	1.09	10.27	0.87
ENSGACG00000019053	-	0	1	1	0	102.30	12.66	18754.10	1457.29
ENSGACG00000019056	-	0	1	0	0	176.34	58.75	9339.20	2705.08
ENSGACG00000019061	-	0	1	0	0	307.54	96.13	0.00	1384.05
ENSGACG00000019063	-	0	1	0	0	106.41	24.70	7663.68	513.99
ENSGACG00000019064	-	0	1	0	0	21.04	144.38	2241.10	1971.81
ENSGACG00000019068	*PYROXD1*	0	1	0	0	8.57	11.39	17.99	15.91
ENSGACG00000019078	-	0	1	0	0	47.82	76.68	6.66	5.45
ENSGACG00000019081	-	0	1	1	1	1.67	1.21	4.81	1.23
ENSGACG00000019085	*F13A1*	0	0	0	1	43.72	61.71	13.56	4.41
ENSGACG00000019087	*SLC5A8*	0	1	1	1	0.19	0.42	28.40	1.63
ENSGACG00000019098	*ARL1*	0	1	0	0	26.89	43.20	82.15	64.21
ENSGACG00000019133	-	1	0	0	0	0.14	4.69	0.36	0.56
ENSGACG00000019143	*KLF1*	1	0	0	0	3.35	14.66	4.18	1.31
ENSGACG00000019160	*CAMK1D (1 of 2)*	0	1	0	0	20.95	3.80	1.56	3.06
ENSGACG00000019183	-	0	0	0	1	4.63	7.09	0.79	3.52
ENSGACG00000019202	*COA6*	0	1	0	0	23.17	44.68	27.39	38.18
ENSGACG00000019209	*SEC23B*	0	1	1	0	11.09	19.29	47.96	47.71
ENSGACG00000019221	*LARGE*	0	0	0	1	4.92	2.01	1.22	4.54
ENSGACG00000019231	*CCDC88A (2 of 2)*	1	0	0	0	0.50	17.16	1.30	0.07
ENSGACG00000019246	-	0	0	0	1	48.79	21.83	42.34	17.81
ENSGACG00000019250	*TM4SF5*	0	1	0	0	1.87	13.07	11.34	15.49
ENSGACG00000019252	-	0	0	0	1	59.71	131.96	17.71	5.67
ENSGACG00000019256	*SRP68*	0	1	0	0	18.56	23.48	43.37	53.24
ENSGACG00000019265	-	0	1	0	0	11.39	14.56	23.61	28.94
ENSGACG00000019290	*TTC26*	0	1	0	0	17.96	23.30	23.57	30.91
ENSGACG00000019291	*IRAK3*	0	0	0	1	19.63	14.66	26.81	4.36
ENSGACG00000019297	*STIM2*	0	1	0	0	4.14	3.97	7.70	4.24
ENSGACG00000019313	*TTLL12*	0	1	1	1	4.08	12.47	41.23	12.29
ENSGACG00000019326	-	0	0	0	1	124.40	749.53	62.76	20.06
ENSGACG00000019327	-	0	0	0	1	16.22	48.51	9.91	1.79
ENSGACG00000019332	*EVI5L*	0	1	1	0	9.47	3.16	19.36	19.67
ENSGACG00000019333	*ALDH1L2*	0	1	0	0	0.39	1.56	2.06	0.67
ENSGACG00000019336	*SLC41A2 (1 of 2)*	0	1	1	0	8.55	6.87	18.84	13.69
ENSGACG00000019337	-	0	1	1	0	8.01	5.42	22.36	12.45
ENSGACG00000019338	-	0	1	1	0	6.07	5.38	19.71	9.37
ENSGACG00000019342	-	0	1	0	0	67.25	31.95	14.31	30.81
ENSGACG00000019343	*PODXL*	0	1	0	0	114.38	49.76	20.81	44.36
ENSGACG00000019347	*SLC16A13*	0	0	0	1	4.81	2.27	0.53	4.17
ENSGACG00000019365	*SHBG*	1	1	0	0	9.60	1.01	0.59	1.08
ENSGACG00000019391	*SLC26A3 (2 of 2)*	0	0	1	0	0.30	0.03	1.15	0.07
ENSGACG00000019394	*HN1 (1 of 2)*	0	1	1	0	9.80	2.79	22.72	48.39
ENSGACG00000019423	*HGFAC*	0	1	1	1	0.10	0.05	2.83	0.26
ENSGACG00000019432	-	0	0	0	1	1.10	1.01	0.19	2.41
ENSGACG00000019467	*CSRP2*	0	1	1	1	13.33	27.21	190.90	76.83

ENSGACG00000019483	-	0	0	0	1	4.80	4.64	6.97	2.53
ENSGACG00000019485	*CMAHP (1 of 2)*	0	0	0	1	0.83	0.54	0.68	0.19
ENSGACG00000019494	-	0	1	0	0	0.16	0.47	1.21	0.30
ENSGACG00000019509	*ANO4*	0	1	0	0	17.07	5.88	1.89	1.29
ENSGACG00000019512	*NXPE3 (8 of 8)*	0	1	0	0	27.90	24.53	5.55	3.34
ENSGACG00000019528	*PTPRD (2 of 2)*	0	1	0	0	32.88	9.51	6.85	18.96
ENSGACG00000019537	*SEC24D*	0	1	0	0	6.49	12.39	28.93	31.02
ENSGACG00000019546	*PPARGC1A*	0	0	0	1	27.47	11.62	3.56	23.72
ENSGACG00000019547	*TXNDC11*	0	1	1	0	8.27	7.92	30.21	40.46
ENSGACG00000019555	*TMEM129*	0	1	0	0	13.25	15.24	23.10	18.05
ENSGACG00000019558	*RNF103*	0	1	1	0	18.00	17.24	56.58	39.92
ENSGACG00000019565	-	0	1	0	0	39.74	15.55	6.85	10.99
ENSGACG00000019566	-	0	1	0	0	65.63	31.09	11.95	16.79
ENSGACG00000019569	*CCDC149 (1 of 2)*	0	1	1	0	5.75	3.17	15.53	13.30
ENSGACG00000019575	*LGI2 (1 of 2)*	0	1	0	1	0.54	0.76	1.59	0.39
ENSGACG00000019588	*ENPP6*	0	0	0	1	29.70	11.54	7.26	26.44
ENSGACG00000019601	*OSTC*	0	1	0	0	109.18	202.28	337.69	364.07
ENSGACG00000019622	-	0	0	0	1	9.17	6.07	2.70	13.13
ENSGACG00000019638	*PLIN2*	0	0	0	1	9.13	8.59	2.54	13.36
ENSGACG00000019653	*FGL1 (1 of 2)*	0	1	0	0	2.94	9.56	8.83	4.26
ENSGACG00000019662	*ADIPOR2*	0	1	0	0	30.93	24.60	53.27	31.97
ENSGACG00000019686	*BANK1*	1	0	0	0	2.01	0.14	0.16	1.16
ENSGACG00000019694	*CACNA1C*	1	0	0	0	1.07	0.11	0.20	0.42
ENSGACG00000019730	*TP53BP2 (2 of 2)*	0	1	0	0	1.38	0.30	0.15	0.35
ENSGACG00000019732	*COPG2*	0	1	0	0	41.09	49.51	53.03	53.73
ENSGACG00000019746	*IQSEC3 (1 of 2)*	0	1	1	1	0.06	0.11	1.84	0.10
ENSGACG00000019748	-	1	1	0	0	18.59	2.92	0.81	1.80
ENSGACG00000019750	-	1	0	0	0	9.85	1.08	2.86	1.22
ENSGACG00000019757	-	0	1	0	0	29.85	46.16	112.02	127.18
ENSGACG00000019758	*PPP1R14B*	0	1	1	0	29.82	26.93	89.52	118.29
ENSGACG00000019784	*MYOM2 (2 of 2)*	1	0	0	0	1.48	0.14	0.17	0.15
ENSGACG00000019801	-	0	0	0	1	13.38	11.93	3.70	0.76
ENSGACG00000019823	-	1	0	0	0	2.71	12.93	1.67	1.99
ENSGACG00000019826	-	0	0	0	1	2.92	1.47	3.21	0.33
ENSGACG00000019833	-	0	1	0	0	2.48	2.48	0.24	1.02
ENSGACG00000019851	*CKAP4*	0	1	1	0	3.81	3.76	35.43	30.18
ENSGACG00000019855	*ARFGAP3*	0	1	1	0	33.57	23.17	63.96	52.64
ENSGACG00000019860	-	0	1	0	0	14.44	8.66	2.96	5.71
ENSGACG00000019866	*GNPTAB*	0	1	1	0	10.60	5.75	13.15	10.38
ENSGACG00000019869	*CHPT1*	0	1	0	0	18.37	14.09	22.86	20.01
ENSGACG00000019888	-	0	1	0	0	3.05	0.49	0.17	0.98
ENSGACG00000019890	*PSEN2*	0	1	0	0	34.55	24.04	45.92	45.75
ENSGACG00000019893	-	1	0	1	0	0.11	1.71	0.07	0.19
ENSGACG00000019895	-	0	1	0	0	17.92	4.60	0.67	1.88
ENSGACG00000019896	*RPGRIP1*	0	1	0	0	0.39	0.02	0.00	0.16
ENSGACG00000019900	*PCED1A*	0	0	1	0	4.43	2.71	0.00	0.00
ENSGACG00000019912	-	1	0	0	0	6.31	44.36	8.89	5.84
ENSGACG00000019920	*KDM6B (1 of 2)*	0	0	0	1	15.35	7.47	4.15	13.07
ENSGACG00000019933	*URGCP*	0	0	1	0	9.38	18.33	2.37	10.81
ENSGACG00000019950	-	0	1	0	0	155.98	125.99	405.88	271.87
ENSGACG00000019953	-	0	1	0	0	8.63	2.81	0.62	1.91
ENSGACG00000019954	-	0	1	0	0	11.17	2.53	0.18	0.79
ENSGACG00000019958	-	0	1	0	0	6.98	2.14	0.38	0.69
ENSGACG00000019967	-	0	1	0	0	6.73	1.90	0.75	1.94
ENSGACG00000019969	-	0	0	1	0	0.85	3.21	0.09	0.41
ENSGACG00000019972	-	0	0	1	0	1.72	4.27	0.29	0.69
ENSGACG00000019974	-	0	0	1	0	0.61	2.86	0.09	0.32
ENSGACG00000019987	*GOLGB1*	0	1	1	0	6.28	4.36	10.48	13.30
ENSGACG00000019988	-	0	1	0	0	8.48	8.85	16.08	15.03
ENSGACG00000019992	*LLPH*	0	1	0	0	51.47	190.83	124.86	164.40
ENSGACG00000020037	*CRY1 (1 of 2)*	0	1	0	0	5.00	1.35	0.55	1.73
ENSGACG00000020056	*NTN4 (2 of 2)*	0	1	0	0	4.06	0.87	0.54	0.66
ENSGACG00000020057	*SLC29A2 (1 of 2)*	0	0	0	1	3.76	3.26	3.25	12.54
ENSGACG00000020078	*MPDU1 (1 of 2)*	0	1	0	0	37.75	43.94	65.99	55.77
ENSGACG00000020083	*CREB3L2*	0	1	0	0	2.58	2.22	4.90	6.37
ENSGACG00000020085	*DGKI*	0	0	0	1	10.21	13.47	2.67	1.20

ENSGACG00000020090	-	0	1	0	0	20.18	12.79	2.37	12.69
ENSGACG00000020099	-	0	1	1	1	0.40	0.11	1.81	0.30
ENSGACG00000020105	-	0	1	0	0	18.67	8.41	2.89	6.05
ENSGACG00000020113	*HYOU1*	0	1	1	0	25.72	34.10	72.09	108.05
ENSGACG00000020124	-	0	0	0	1	1.84	0.53	0.03	1.44
ENSGACG00000020156	*WSCD1*	0	1	0	0	2.56	0.78	0.01	0.09
ENSGACG00000020166	*SLC8A2 (2 of 2)*	0	1	1	0	19.11	16.21	157.20	123.50
ENSGACG00000020168	*CKM (1 of 2)*	0	1	0	0	69.57	1.61	0.20	0.52
ENSGACG00000020174	-	0	1	0	0	75.15	99.14	120.78	130.00
ENSGACG00000020183	*JAM3 (1 of 2)*	0	1	1	0	5.24	2.85	11.19	10.19
ENSGACG00000020193	*STT3A*	0	1	0	0	85.09	144.22	236.16	330.05
ENSGACG00000020196	*WNT11*	0	1	0	0	14.27	5.60	1.41	4.17
ENSGACG00000020228	*P2RX1*	0	0	0	1	6.90	8.25	2.31	0.79
ENSGACG00000020236	-	0	0	1	0	6.88	2.16	7.18	7.81
ENSGACG00000020240	*CA4 (1 of 3)*	1	1	0	0	16.14	1.35	1.06	6.48
ENSGACG00000020253	*TRPV2*	0	0	0	1	16.92	9.72	8.14	1.77
ENSGACG00000020259	*CENPV*	0	1	0	1	8.94	11.34	12.16	5.17
ENSGACG00000020272	*STIP1*	0	1	0	0	42.86	110.66	122.42	92.65
ENSGACG00000020275	*ZFPL1*	0	1	0	0	15.88	22.59	27.57	21.84
ENSGACG00000020298	*PCOLCE (2 of 2)*	0	0	0	1	59.33	81.95	58.60	23.70
ENSGACG00000020303	*KSR1 (1 of 2)*	0	1	0	0	10.22	4.92	1.40	2.01
ENSGACG00000020317	*HRH2 (1 of 2)*	0	1	0	1	12.74	1.80	0.23	24.89
ENSGACG00000020382	*RRM1 (2 of 2)*	0	0	0	1	3.41	8.96	1.27	0.41
ENSGACG00000020400	*SLC25A2*	0	0	0	1	9.12	6.59	3.16	20.01
ENSGACG00000020402	*MPDU1 (2 of 2)*	0	1	0	0	9.90	4.82	0.85	4.33
ENSGACG00000020403	-	0	1	1	0	15.86	15.01	1.23	15.58
ENSGACG00000020406	-	0	1	0	0	16.51	6.27	1.71	2.42
ENSGACG00000020412	*DPAGT1*	0	1	0	0	15.76	21.31	32.99	34.59
ENSGACG00000020422	*F11R*	0	0	1	0	54.04	20.56	50.19	68.70
ENSGACG00000020444	*EHBP1L1 (2 of 2)*	0	1	0	0	16.45	12.79	3.49	2.87
ENSGACG00000020456	*UBLCP1*	0	1	0	0	10.07	12.82	16.82	23.28
ENSGACG00000020461	*BNIP1 (2 of 2)*	0	1	0	0	12.86	23.95	17.10	14.21
ENSGACG00000020469	-	0	0	1	0	13.51	2.49	7.14	5.56
ENSGACG00000020542	-	0	1	1	0	0.05	0.00	1.43	0.02
ENSGACG00000020546	*USPL1*	0	1	0	1	25.91	12.44	4.74	18.99
ENSGACG00000020548	*MEDAG*	0	1	0	0	17.09	7.31	1.92	4.94
ENSGACG00000020551	*FRY (2 of 2)*	0	1	0	0	3.29	1.13	0.52	0.74
ENSGACG00000020555	*STARD13 (2 of 2)*	0	1	0	0	7.00	2.01	1.19	2.30
ENSGACG00000020556	*NBEA (2 of 2)*	0	1	0	0	12.48	3.20	2.12	3.52
ENSGACG00000020563	*ALG5*	0	1	0	0	13.90	19.22	30.08	27.23
ENSGACG00000020566	-	0	1	0	0	28.53	4.01	0.45	1.60
ENSGACG00000020567	*POSTN (2 of 2)*	0	1	1	1	1.76	2.73	14.05	2.42
ENSGACG00000020572	*ARFIP2 (1 of 2)*	0	1	1	0	12.67	17.94	63.79	58.54
ENSGACG00000020582	*TIAM1 (2 of 2)*	1	1	0	0	5.50	1.06	0.32	1.13
ENSGACG00000020589	*FAT3 (2 of 2)*	0	1	0	1	15.15	3.50	0.70	8.36
ENSGACG00000020600	*SLC7A1 (1 of 2)*	0	1	0	0	13.95	4.66	1.03	2.62
ENSGACG00000020605	*HEPHL1*	0	1	1	0	13.69	18.56	203.11	13.97
ENSGACG00000020606	*SLC43A2 (2 of 2)*	0	0	0	1	71.79	28.06	42.45	19.32
ENSGACG00000020609	*NLE1*	0	1	0	0	10.39	16.66	14.57	23.50
ENSGACG00000020612	-	0	0	0	1	17.48	10.39	16.10	171.43
ENSGACG00000020619	-	0	0	0	1	119.14	161.07	44.37	17.23
ENSGACG00000020620	*PIGS*	0	1	0	0	12.11	18.79	15.87	12.77
ENSGACG00000020622	-	0	0	1	0	25.44	41.52	5.39	13.17
ENSGACG00000020635	*CHEK1*	0	0	0	1	40.78	40.86	15.88	125.68
ENSGACG00000020636	*C2 (2 of 2)*	0	1	0	0	0.14	0.24	2.86	0.27
ENSGACG00000020657	*CXXC5*	0	1	0	0	8.23	2.83	0.96	2.83
ENSGACG00000020673	-	0	0	0	1	15.41	15.19	12.12	5.78
ENSGACG00000020678	*PRDX4*	0	1	0	0	72.50	235.88	608.90	363.00
ENSGACG00000020683	*WASF3 (2 of 2)*	0	0	0	1	1.69	0.69	1.62	0.54
ENSGACG00000020724	*YIPF5*	0	1	0	0	20.05	32.31	40.92	40.74
ENSGACG00000020755	*TBL2*	0	1	0	0	9.01	11.34	17.81	20.09
ENSGACG00000020776	*PLEKHB1*	0	1	1	1	0.04	0.53	16.00	1.40
ENSGACG00000020820	*GALNT10 (1 of 2)*	0	1	1	0	7.57	4.80	54.23	18.18
ENSGACG00000020821	*GALNT10 (2 of 2)*	0	1	1	0	0.69	0.26	3.14	1.32
ENSGACG00000020823	*FAM114A2*	0	1	0	0	14.87	13.55	20.73	28.21
ENSGACG00000020827	*NCF1*	0	0	1	1	97.22	224.90	32.17	10.11

ENSGACG00000020828	-	0	0	0	1	15.80	24.71	3.58	1.62
ENSGACG00000020829	*ST6GAL1*	1	0	0	0	1.94	8.36	1.91	0.98
ENSGACG00000020873	-	1	0	1	1	25.62	2.48	9.96	1.34
ENSGACG00000020874	-	0	1	0	0	209.63	20.10	2.83	5.70
ENSGACG00000020885	-	0	1	0	0	17.80	16.09	26.38	21.40
ENSGACG00000020917	-	0	1	1	0	388.90	62.63	0.98	47.53
ENSGACG00000020918	*SLC23A1*	0	1	0	0	42.99	14.10	2.64	23.21

Supplementary Table 4.
560 HTN genes identified using non-GWA approaches

1	*ABCA1*	ATP-binding cassette, sub-family A (ABC1), member 1
2	*ABCB1*	ATP-binding cassette, sub-family B (MDR/TAP), member 1
3	*ACAT1*	acetyl-CoA acetyltransferase 1
4	*ACE*	angiotensin I converting enzyme (peptidyl-dipeptidase A) 1
5	*ACE2*	angiotensin I converting enzyme (peptidyl-dipeptidase A) 2
6	*ACPL2*	acid phosphatase-like 2
7	*ACSM3*	acyl-CoA synthetase medium-chain family member 3
8	*ACVRL1*	activin A receptor type II-like 1
9	*ADAMTS13*	ADAM metallopeptidase with thrombospondin type 1 motif, 13
10	*ADD1*	adducin 1 (alpha)
11	*ADD2*	adducin 2 (beta)
12	*ADD3*	adducin 3 (gamma)
13	*ADH7*	alcohol dehydrogenase 7 (class IV), mu or sigma polypeptide
14	*ADIPOQ*	adiponectin, C1Q and collagen domain containing
15	*ADORA1*	adenosine A1 receptor
16	*ADORA2A*	adenosine A2a receptor
17	*ADORA2B*	adenosine A2b receptor
18	*ADRA1A*	adrenergic, alpha-1A-, receptor
19	*ADRA1B*	adrenergic, alpha-1B-, receptor
20	*ADRA2A*	adrenergic, alpha-2A-, receptor
21	*ADRA2B*	adrenergic, alpha-2B-, receptor
22	*ADRB2*	adrenergic, beta-2-, receptor, surface
23	*ADRB3*	adrenergic, beta-3-, receptor
24	*AFM*	afamin
25	*AGTR1*	angiotensin II receptor, type 1
26	*AGTR2*	angiotensin II receptor, type 2
27	*AKT1*	v-akt murine thymoma viral oncogene homolog 1
28	*ALAD*	aminolevulinate dehydratase
29	*ALDH1A2*	aldehyde dehydrogenase 1 family, member A2
30	*ALOX12*	arachidonate 12-lipoxygenase
31	*AMPD2*	adenosine monophosphate deaminase 2
32	*ANGPT1*	angiopoietin 1
33	*ANGPT2*	angiopoietin 2
34	*ANPEP*	alanyl (membrane) aminopeptidase
35	*AOX1*	aldehyde oxidase 1
36	*APEX1*	APEX nuclease (multifunctional DNA repair enzyme) 1
37	*APLN*	apelin
38	*APLNR*	apelin receptor
39	*APOA1*	apolipoprotein A-I
40	*APOA2*	apolipoprotein A-II
41	*APOB*	apolipoprotein B (including Ag(x) antigen)
42	*APOC2*	apolipoprotein C-II
43	*APOC3*	apolipoprotein C-III
44	*APOC4*	apolipoprotein C-IV
45	*APOE*	apolipoprotein E
46	*APOH*	apolipoprotein H (beta-2-glycoprotein I)
47	*AQP2*	aquaporin 2 (collecting duct)
48	*AQP4*	aquaporin 4
49	*AR*	androgen receptor
50	*ARG1*	arginase, liver
51	*ARG2*	arginase, type II
52	*ARHGAP8*	Rho GTPase activating protein 8
53	*ARHGEF1*	Rho guanine nucleotide exchange factor (GEF) 1
54	*ARHGEF6*	Rac/Cdc42 guanine nucleotide exchange factor (GEF) 6
55	*ARSG*	arylsulfatase G
56	*ATP1A1*	ATPase, Na+/K+ transporting, alpha 1 polypeptide
57	*ATP1A2*	ATPase, Na+/K+ transporting, alpha 2 polypeptide
58	*ATP1B1*	ATPase, Na+/K+ transporting, beta 1 polypeptide
59	*ATP2A2*	ATPase, Ca++ transporting, cardiac muscle, slow twitch 2
60	*ATP5B*	ATP synthase, H+ transporting, mitochondrial F1 complex, beta polypeptide
61	*ATP5J*	ATP synthase, H+ transporting, mitochondrial Fo complex, subunit F6
62	*AVP*	arginine vasopressin

63	*AVPR1A*	arginine vasopressin receptor 1A
64	*AVPR1B*	arginine vasopressin receptor 1B
65	*AVPR2*	arginine vasopressin receptor 2
66	*BANK1*	B-cell scaffold protein with ankyrin repeats 1
67	*BDKRB1*	bradykinin receptor B1
68	*BDKRB2*	bradykinin receptor B2
69	*BDNF*	brain-derived neurotrophic factor
70	*BGN*	biglycan
71	*BHMT*	betaine--homocysteine S-methyltransferase
72	*BLVRA*	biliverdin reductase A
73	*BMP10*	bone morphogenetic protein 10
74	*BMP2*	bone morphogenetic protein 2
75	*BMP4*	bone morphogenetic protein 4
76	*BMP7*	bone morphogenetic protein 7
77	*BMPR1B*	bone morphogenetic protein receptor, type IB
78	*BMPR2*	bone morphogenetic protein receptor, type II (serine/threonine kinase)
79	*BRS3*	bombesin-like receptor 3
80	*BTN2A1*	butyrophilin, subfamily 2, member A1
81	*C1QTNF1*	C1q and tumor necrosis factor related protein 1
82	*C3*	complement component 3
83	*CACNA1C*	calcium channel, voltage-dependent, L type, alpha 1C subunit
84	*CACNA1D*	calcium channel, voltage-dependent, L type, alpha 1D subunit
85	*CALCA*	calcitonin-related polypeptide alpha
86	*CALCRL*	calcitonin receptor-like
87	*CAMK4*	calcium/calmodulin-dependent protein kinase IV
88	*CAPN10*	calpain 10
89	*CASP8*	caspase 8, apoptosis-related cysteine peptidase
90	*CAT*	catalase
91	*CAV1*	caveolin 1, caveolae protein, 22kDa
92	*CCL2*	chemokine (C-C motif) ligand 2
93	*CCL20*	chemokine (C-C motif) ligand 20
94	*CCR2*	chemokine (C-C motif) receptor 2
95	*CCR5*	chemokine (C-C motif) receptor 5
96	*CD36*	CD36 molecule (thrombospondin receptor)
97	*CD40*	CD40 molecule, TNF receptor superfamily member 5
98	*CD40LG*	CD40 ligand
99	*CDKN2A*	cyclin-dependent kinase inhibitor 2A (melanoma, p16, inhibits CDK4)
100	*CETP*	cholesteryl ester transfer protein, plasma
101	*CFH*	complement factor H
102	*CHEK2*	CHK2 checkpoint homolog (S. pombe)
103	*CHGA*	chromogranin A (parathyroid secretory protein 1)
104	*CHGB*	chromogranin B (secretogranin 1)
105	*CHIC2*	cysteine-rich hydrophobic domain 2
106	*CLCNKA*	chloride channel Ka
107	*CLCNKB*	chloride channel Kb
108	*CLU*	clusterin
109	*CNTN4*	contactin 4
110	*COMT*	catechol-O-methyltransferase
111	*COPS5*	COP9 constitutive photomorphogenic homolog subunit 5 (Arabidopsis)
112	*CORIN*	corin, serine peptidase
113	*CPS1*	carbamoyl-phosphate synthase 1, mitochondrial
114	*CRP*	C-reactive protein, pentraxin-related
115	*CSMD1*	CUB and Sushi multiple domains 1
116	*CST3*	cystatin C
117	*CTF1*	cardiotrophin 1
118	*CTGF*	connective tissue growth factor
119	*CTH*	cystathionase (cystathionine gamma-lyase)
120	*CTNNB1*	catenin (cadherin-associated protein), beta 1, 88kDa
121	*CX3CL1*	chemokine (C-X3-C motif) ligand 1
122	*CX3CR1*	chemokine (C-X3-C motif) receptor 1
123	*CXCL10*	chemokine (C-X-C motif) ligand 10
124	*CXCL12*	chemokine (C-X-C motif) ligand 12
125	*CXCL16*	chemokine (C-X-C motif) ligand 16
126	*CYBA*	cytochrome b-245, alpha polypeptide
127	*CYP11A1*	cytochrome P450, family 11, subfamily A, polypeptide 1
128	*CYP11B1*	cytochrome P450, family 11, subfamily B, polypeptide 1

129	*CYP19A1*	cytochrome P450, family 19, subfamily A, polypeptide 1
130	*CYP21A2*	cytochrome P450, family 21, subfamily A, polypeptide 2
131	*CYP2C19*	cytochrome P450, family 2, subfamily C, polypeptide 19
132	*CYP2C8*	cytochrome P450, family 2, subfamily C, polypeptide 8
133	*CYP2C9*	cytochrome P450, family 2, subfamily C, polypeptide 9
134	*CYP2D6*	cytochrome P450, family 2, subfamily D, polypeptide 6
135	*CYP2D7P1*	cytochrome P450, family 2, subfamily D, polypeptide 7 pseudogene 1
136	*CYP2J2*	cytochrome P450, family 2, subfamily J, polypeptide 2
137	*CYP3A4*	cytochrome P450, family 3, subfamily A, polypeptide 4
138	*CYP3A5*	cytochrome P450, family 3, subfamily A, polypeptide 5
139	*CYP4A11*	cytochrome P450, family 4, subfamily A, polypeptide 11
140	*CYP4A22*	cytochrome P450, family 4, subfamily A, polypeptide 22
141	*CYP4F2*	cytochrome P450, family 4, subfamily F, polypeptide 2
142	*DBH*	dopamine beta-hydroxylase (dopamine beta-monooxygenase)
143	*DCPS*	decapping enzyme, scavenger
144	*DDAH2*	dimethylarginine dimethylaminohydrolase 2
145	*DDT*	D-dopachrome tautomerase
146	*DGAT2*	diacylglycerol O-acyltransferase 2
147	*DIO2*	deiodinase, iodothyronine, type II
148	*DPEP1*	dipeptidase 1 (renal)
149	*DPP4*	dipeptidyl-peptidase 4
150	*DPT*	dermatopontin
151	*DRD1*	dopamine receptor D1
152	*DRD2*	dopamine receptor D2
153	*ECE1*	endothelin converting enzyme 1
154	*EDN1*	endothelin 1
155	*EDN2*	endothelin 2
156	*EDNRA*	endothelin receptor type A
157	*EDNRB*	endothelin receptor type B
158	*EMILIN1*	elastin microfibril interfacer 1
159	*ENG*	endoglin
160	*ENPP1*	ectonucleotide pyrophosphatase/phosphodiesterase 1
161	*EPHX2*	epoxide hydrolase 2, cytoplasmic
162	*EPO*	erythropoietin
163	*ERAP1*	endoplasmic reticulum aminopeptidase 1
164	*ESR1*	estrogen receptor 1
165	*ESR2*	estrogen receptor 2 (ER beta)
166	*F11R*	F11 receptor
167	*F2*	coagulation factor II (thrombin)
168	*F2R*	coagulation factor II (thrombin) receptor
169	*F3*	coagulation factor III (thromboplastin, tissue factor)
170	*F5*	coagulation factor V (proaccelerin, labile factor)
171	*F7*	coagulation factor VII (serum prothrombin conversion accelerator)
172	*FABP4*	fatty acid binding protein 4, adipocyte
173	*FAM98A*	family with sequence similarity 98, member A
174	*FBN1*	fibrillin 1
175	*FGA*	fibrinogen alpha chain
176	*FGB*	fibrinogen beta chain
177	*FGF2*	fibroblast growth factor 2 (basic)
178	*FGFBP1*	fibroblast growth factor binding protein 1
179	*FGG*	fibrinogen gamma chain
180	*FH*	fumarate hydratase
181	*FHL1*	four and a half LIM domains 1
182	*FHL2*	four and a half LIM domains 2
183	*FLT1*	fms-related tyrosine kinase 1 (vascular endothelial growth factor/vascular permeability factor receptor)
184	*FMO3*	flavin containing monooxygenase 3
185	*FSTL4*	follistatin-like 4
186	*FUT3*	fucosyltransferase 3 (galactoside 3(4)-L-fucosyltransferase, Lewis blood group)
187	*FUT4*	fucosyltransferase 4 (alpha (1,3) fucosyltransferase, myeloid-specific)
188	*GCG*	glucagon
189	*GCGR*	glucagon receptor
190	*GCK*	glucokinase (hexokinase 4)
191	*GCLC*	glutamate-cysteine ligase, catalytic subunit
192	*GDF15*	growth differentiation factor 15
193	*GGT1*	gamma-glutamyltransferase 1
194	*GH1*	growth hormone 1

195	*GH2*	growth hormone 2
196	*GHR*	growth hormone receptor
197	*GHRL*	ghrelin/obestatin prepropeptide
198	*GIPR*	gastric inhibitory polypeptide receptor
199	*GLP1R*	glucagon-like peptide 1 receptor
200	*GM2A*	GM2 ganglioside activator
201	*GNAI1*	guanine nucleotide binding protein (G protein), alpha inhibiting activity polypeptide 1
202	*GNB3*	guanine nucleotide binding protein (G protein), beta polypeptide 3
203	*GNB5*	guanine nucleotide binding protein (G protein), beta 5
204	*GPR39*	G protein-coupled receptor 39
205	*GPX1*	glutathione peroxidase 1
206	*GREM1*	gremlin 1
207	*GRK4*	G protein-coupled receptor kinase 4
208	*GSTA1*	glutathione S-transferase alpha 1
209	*GSTM1*	glutathione S-transferase mu 1
210	*GSTM3*	glutathione S-transferase mu 3 (brain)
211	*GSTP1*	glutathione S-transferase pi 1
212	*GSTT1*	glutathione S-transferase theta 1
213	*GSTT2*	glutathione S-transferase theta 2
214	*GUCA2B*	guanylate cyclase activator 2B (uroguanylin)
215	*GULP1*	GULP, engulfment adaptor PTB domain containing 1
216	*GYS1*	glycogen synthase 1 (muscle)
217	*GYS2*	glycogen synthase 2 (liver)
218	*HEY1*	hairy/enhancer-of-split related with YRPW motif 1
219	*HGF*	hepatocyte growth factor (hepapoietin A; scatter factor)
220	*HIF1A*	hypoxia inducible factor 1, alpha subunit (basic helix-loop-helix transcription factor)
221	*HLA-A*	major histocompatibility complex, class I, A
222	*HLA-B*	major histocompatibility complex, class I, B
223	*HLA-DRB1*	major histocompatibility complex, class II, DR beta 1
224	*HMOX1*	heme oxygenase (decycling) 1
225	*HMOX2*	heme oxygenase (decycling) 2
226	*HP*	haptoglobin
227	*HRH1*	histamine receptor H1
228	*HSD11B1*	hydroxysteroid (11-beta) dehydrogenase 1
229	*HSD11B2*	hydroxysteroid (11-beta) dehydrogenase 2
230	*HSD3B1*	hydroxy-delta-5-steroid dehydrogenase, 3 beta- and steroid delta-isomerase 1
231	*HSD3B2*	hydroxy-delta-5-steroid dehydrogenase, 3 beta- and steroid delta-isomerase 2
232	*HSPA1A*	heat shock 70kDa protein 1A
233	*HSPA1B*	heat shock 70kDa protein 1B
234	*HSPA1L*	heat shock 70kDa protein 1-like
235	*HTR2A*	5-hydroxytryptamine (serotonin) receptor 2A
236	*HYOU1*	hypoxia up-regulated 1
237	*IAPP*	islet amyloid polypeptide
238	*ICAM1*	intercellular adhesion molecule 1
239	*ID1*	inhibitor of DNA binding 1, dominant negative helix-loop-helix protein
240	*ID2*	inhibitor of DNA binding 2, dominant negative helix-loop-helix protein
241	*IER3*	immediate early response 3
242	*IGF1*	insulin-like growth factor 1 (somatomedin C)
243	*IGF1R*	insulin-like growth factor 1 receptor
244	*IGF2*	insulin-like growth factor 2 (somatomedin A)
245	*IL10*	interleukin 10
246	*IL12B*	interleukin 12B (natural killer cell stimulatory factor 2, cytotoxic lymphocyte maturation factor 2, p40)
247	*IL15*	interleukin 15
248	*IL18*	interleukin 18 (interferon-gamma-inducing factor)
249	*IL1A*	interleukin 1, alpha
250	*IL1B*	interleukin 1, beta
251	*IL1RN*	interleukin 1 receptor antagonist
252	*IL23R*	interleukin 23 receptor
253	*IL6*	interleukin 6 (interferon, beta 2)
254	*ILF3*	interleukin enhancer binding factor 3, 90kDa
255	*INHA*	inhibin, alpha
256	*INHBA*	inhibin, beta A
257	*INPPL1*	inositol polyphosphate phosphatase-like 1
258	*INS*	insulin
259	*INSR*	insulin receptor
260	*IRS1*	insulin receptor substrate 1

261	*ITGB3*	integrin, beta 3 (platelet glycoprotein IIIa, antigen CD61)
262	*JUN*	jun proto-oncogene
263	*KCNA5*	potassium voltage-gated channel, shaker-related subfamily, member 5
264	*KCNJ1*	potassium inwardly-rectifying channel, subfamily J, member 1
265	*KCNJ11*	potassium inwardly-rectifying channel, subfamily J, member 11
266	*KCNJ6*	potassium inwardly-rectifying channel, subfamily J, member 6
267	*KCNK3*	potassium channel, subfamily K, member 3
268	*KCNMA1*	potassium large conductance calcium-activated channel, subfamily M, alpha member 1
269	*KCNMB1*	potassium large conductance calcium-activated channel, subfamily M, beta member 1
270	*KDR*	kinase insert domain receptor (a type III receptor tyrosine kinase)
271	*KIF1C*	kinesin family member 1C
272	*KL*	klotho
273	*KLC1*	kinesin light chain 1
274	*KLF5*	Kruppel-like factor 5 (intestinal)
275	*KLK1*	kallikrein 1
276	*KLKB1*	kallikrein B, plasma (Fletcher factor) 1
277	*KNG1*	kininogen 1
278	*KYNU*	kynureninase
279	*LANCL1*	LanC lantibiotic synthetase component C-like 1 (bacterial)
280	*LCN2*	lipocalin 2
281	*LDLR*	low density lipoprotein receptor
282	*LEP*	leptin
283	*LEPR*	leptin receptor
284	*LGALS1*	lectin, galactoside-binding, soluble, 1
285	*LIPC*	lipase, hepatic
286	*LIPE*	lipase, hormone-sensitive
287	*LMNA*	lamin A/C
288	*LPA*	lipoprotein, Lp(a)
289	*LPL*	lipoprotein lipase
290	*LRP5*	low density lipoprotein receptor-related protein 5
291	*LTA*	lymphotoxin alpha (TNF superfamily, member 1)
292	*LY6H*	lymphocyte antigen 6 complex, locus H
293	*LYZ*	lysozyme
294	*MACROD2*	MACRO domain containing 2
295	*MAMDC2*	MAM domain containing 2
296	*MAOA*	monoamine oxidase A
297	*MAP1LC3B*	microtubule-associated protein 1 light chain 3 beta
298	*MAPK1*	mitogen-activated protein kinase 1
299	*MAPK14*	mitogen-activated protein kinase 14
300	*MAPK8*	mitogen-activated protein kinase 8
301	*MAT1A*	methionine adenosyltransferase I, alpha
302	*MAT2A*	methionine adenosyltransferase II, alpha
303	*MBL2*	mannose-binding lectin (protein C) 2, soluble
304	*MC4R*	melanocortin 4 receptor
305	*MCAM*	melanoma cell adhesion molecule
306	*MDM4*	Mdm4 p53 binding protein homolog (mouse)
307	*MEF2A*	myocyte enhancer factor 2A
308	*MEP1B*	meprin A, beta
309	*MEX3C*	mex-3 homolog C (C. elegans)
310	*MFAP3*	microfibrillar-associated protein 3
311	*MFN2*	mitofusin 2
312	*MGLL*	monoglyceride lipase
313	*MIF*	macrophage migration inhibitory factor (glycosylation-inhibiting factor)
314	*MIR204*	microRNA 204
315	*MLYCD*	malonyl-CoA decarboxylase
316	*MMP2*	matrix metallopeptidase 2 (gelatinase A, 72kDa gelatinase, 72kDa type IV collagenase)
317	*MMP3*	matrix metallopeptidase 3 (stromelysin 1, progelatinase)
318	*MMP9*	matrix metallopeptidase 9 (gelatinase B, 92kDa gelatinase, 92kDa type IV collagenase)
319	*MSRA*	methionine sulfoxide reductase A
320	*MTR*	5-methyltetrahydrofolate-homocysteine methyltransferase
321	*MYADML*	myeloid-associated differentiation marker-like
322	*MYH9*	myosin, heavy chain 9, non-muscle
323	*MYO16*	myosin XVI
324	*MYO6*	myosin VI
325	*MYOC*	myocilin, trabecular meshwork inducible glucocorticoid response
326	*NCF1C*	neutrophil cytosolic factor 1C pseudogene

327	*NCOA3*	nuclear receptor coactivator 3
328	*NDUFC2*	NADH dehydrogenase (ubiquinone) 1, subcomplex unknown, 2, 14.5kDa
329	*NEDD4L*	neural precursor cell expressed, developmentally down-regulated 4-like
330	*NET1*	neuroepithelial cell transforming 1
331	*NFKBIL1*	nuclear factor of kappa light polypeptide gene enhancer in B-cells inhibitor-like 1
332	*NISCH*	nischarin
333	*NOL3*	nucleolar protein 3 (apoptosis repressor with CARD domain)
334	*NOS1*	nitric oxide synthase 1 (neuronal)
335	*NOS2*	nitric oxide synthase 2, inducible
336	*NOTCH3*	notch 3
337	*NOV*	nephroblastoma overexpressed gene
338	*NOX1*	NADPH oxidase 1
339	*NOX3*	NADPH oxidase 3
340	*NOX4*	NADPH oxidase 4
341	*NPHS1*	nephrosis 1, congenital, Finnish type (nephrin)
342	*NPHS2*	nephrosis 2, idiopathic, steroid-resistant (podocin)
343	*NPPC*	natriuretic peptide C
344	*NPR1*	natriuretic peptide receptor A/guanylate cyclase A (atrionatriuretic peptide receptor A)
345	*NPR2*	natriuretic peptide receptor B/guanylate cyclase B (atrionatriuretic peptide receptor B)
346	*NPY*	neuropeptide Y
347	*NPY1R*	neuropeptide Y receptor Y1
348	*NQO1*	NAD(P)H dehydrogenase, quinone 1
349	*NR0B1*	nuclear receptor subfamily 0, group B, member 1
350	*NR1H4*	nuclear receptor subfamily 1, group H, member 4
351	*NR3C1*	nuclear receptor subfamily 3, group C, member 1 (glucocorticoid receptor)
352	*NR3C2*	nuclear receptor subfamily 3, group C, member 2
353	*OPA1*	optic atrophy 1 (autosomal dominant)
354	*OPTN*	optineurin
355	*OTC*	ornithine carbamoyltransferase
356	*P2RY2*	purinergic receptor P2Y, G-protein coupled, 2
357	*PAPPA*	pregnancy-associated plasma protein A, pappalysin 1
358	*PCNA*	proliferating cell nuclear antigen
359	*PCSK6*	proprotein convertase subtilisin/kexin type 6
360	*PDC*	phosducin
361	*PDE4D*	phosphodiesterase 4D, cAMP-specific
362	*PDE5A*	phosphodiesterase 5A, cGMP-specific
363	*PDGFB*	platelet-derived growth factor beta polypeptide
364	*PDX1*	pancreatic and duodenal homeobox 1
365	*PEPD*	peptidase D
366	*PGF*	placental growth factor
367	*PHACTR1*	phosphatase and actin regulator 1
368	*PHOX2A*	paired-like homeobox 2a
369	*PIGL*	phosphatidylinositol glycan anchor biosynthesis, class L
370	*PIK3R1*	phosphoinositide-3-kinase, regulatory subunit 1 (alpha)
371	*PIM1*	pim-1 oncogene
372	*PKD1*	polycystic kidney disease 1 (autosomal dominant)
373	*PKD2*	polycystic kidney disease 2 (autosomal dominant)
374	*PLA2G1B*	phospholipase A2, group IB (pancreas)
375	*PLA2G7*	phospholipase A2, group VII (platelet-activating factor acetylhydrolase, plasma)
376	*PLAT*	plasminogen activator, tissue
377	*PLOD2*	procollagen-lysine, 2-oxoglutarate 5-dioxygenase 2
378	*PMVK*	phosphomevalonate kinase
379	*PNMT*	phenylethanolamine N-methyltransferase
380	*PON1*	paraoxonase 1
381	*POU5F1*	POU class 5 homeobox 1
382	*PPARA*	peroxisome proliferator-activated receptor alpha
383	*PPARG*	peroxisome proliferator-activated receptor gamma
384	*PPARGC1A*	peroxisome proliferator-activated receptor gamma, coactivator 1 alpha
385	*PPP3CA*	protein phosphatase 3, catalytic subunit, alpha isozyme
386	*PRCP*	prolylcarboxypeptidase (angiotensinase C)
387	*PRKCE*	protein kinase C, epsilon
388	*PRKCQ*	protein kinase C, theta
389	*PRKG1*	protein kinase, cGMP-dependent, type I
390	*PRSS8*	protease, serine, 8
391	*PSMA6*	proteasome (prosome, macropain) subunit, alpha type, 6
392	*PSMB9*	proteasome (prosome, macropain) subunit, beta type, 9 (large multifunctional peptidase 2)

393	*PTGER2*	prostaglandin E receptor 2 (subtype EP2), 53kDa
394	*PTGER3*	prostaglandin E receptor 3 (subtype EP3)
395	*PTGES*	prostaglandin E synthase
396	*PTGIR*	prostaglandin I2 (prostacyclin) receptor (IP)
397	*PTGIS*	prostaglandin I2 (prostacyclin) synthase
398	*PTGS1*	prostaglandin-endoperoxide synthase 1 (prostaglandin G/H synthase and cyclooxygenase)
399	*PTGS2*	prostaglandin-endoperoxide synthase 2 (prostaglandin G/H synthase and cyclooxygenase)
400	*PTHLH*	parathyroid hormone-like hormone
401	*PTK2B*	PTK2B protein tyrosine kinase 2 beta
402	*PTPN1*	protein tyrosine phosphatase, non-receptor type 1
403	*PTPN11*	protein tyrosine phosphatase, non-receptor type 11
404	*PTPRJ*	protein tyrosine phosphatase, receptor type, J
405	*PTX3*	pentraxin 3, long
406	*RAMP1*	receptor (G protein-coupled) activity modifying protein 1
407	*RANBP3L*	RAN binding protein 3-like
408	*RASGRP3*	RAS guanyl releasing protein 3 (calcium and DAG-regulated)
409	*RBP4*	retinol binding protein 4, plasma
410	*REN*	renin
411	*RENBP*	renin binding protein
412	*RETN*	resistin
413	*RETNLB*	resistin like beta
414	*RGS2*	regulator of G-protein signaling 2, 24kDa
415	*RGS21*	regulator of G-protein signaling 21
416	*RGS5*	regulator of G-protein signaling 5
417	*RHOA*	ras homolog gene family, member A
418	*RLN1*	relaxin 1
419	*RLN2*	relaxin 2
420	*RNLS*	renalase, FAD-dependent amine oxidase
421	*ROBO4*	roundabout homolog 4, magic roundabout (Drosophila)
422	*ROCK1*	Rho-associated, coiled-coil containing protein kinase 1
423	*ROCK2*	Rho-associated, coiled-coil containing protein kinase 2
424	*ROS1*	c-ros oncogene 1 , receptor tyrosine kinase
425	*RPL30*	ribosomal protein L30
426	*RPL34P1*	ribosomal protein L34 pseudogene 1
427	*RPL6*	ribosomal protein L6
428	*RPP25*	ribonuclease P/MRP 25kDa subunit
429	*RYR2*	ryanodine receptor 2 (cardiac)
430	*S100A4*	S100 calcium binding protein A4
431	*SARS*	seryl-tRNA synthetase
432	*SCG2*	secretogranin II
433	*SCN5A*	sodium channel, voltage-gated, type V, alpha subunit
434	*SCNN1A*	sodium channel, nonvoltage-gated 1 alpha
435	*SCNN1B*	sodium channel, nonvoltage-gated 1, beta
436	*SCNN1D*	sodium channel, nonvoltage-gated 1, delta
437	*SCNN1G*	sodium channel, nonvoltage-gated 1, gamma
438	*SDF2*	stromal cell-derived factor 2
439	*SDK1*	sidekick homolog 1, cell adhesion molecule (chicken)
440	*SELE*	selectin E
441	*SELP*	selectin P (granule membrane protein 140kDa, antigen CD62)
442	*SERPINA1*	serpin peptidase inhibitor, clade A (alpha-1 antiproteinase, antitrypsin), member 1
443	*SERPINA4*	serpin peptidase inhibitor, clade A (alpha-1 antiproteinase, antitrypsin), member 4
444	*SERPINC1*	serpin peptidase inhibitor, clade C (antithrombin), member 1
445	*SERPINE1*	serpin peptidase inhibitor, clade E (nexin, plasminogen activator inhibitor type 1), member 1
446	*SF3B1*	splicing factor 3b, subunit 1, 155kDa
447	*SGK1*	serum/glucocorticoid regulated kinase 1
448	*SHBG*	sex hormone-binding globulin
449	*SLC12A1*	solute carrier family 12 (sodium/potassium/chloride transporters), member 1
450	*SLC12A2*	solute carrier family 12 (sodium/potassium/chloride transporters), member 2
451	*SLC12A3*	solute carrier family 12 (sodium/chloride transporters), member 3
452	*SLC17A1*	solute carrier family 17 (sodium phosphate), member 1
453	*SLC22A1*	solute carrier family 22 (organic cation transporter), member 1
454	*SLC22A2*	solute carrier family 22 (organic cation transporter), member 2
455	*SLC22A3*	solute carrier family 22 (extraneuronal monoamine transporter), member 3
456	*SLC22A6*	solute carrier family 22 (organic anion transporter), member 6
457	*SLC22A8*	solute carrier family 22 (organic anion transporter), member 8
458	*SLC26A4*	solute carrier family 26, member 4

459	*SLC2A12*	solute carrier family 2 (facilitated glucose transporter), member 12
460	*SLC2A2*	solute carrier family 2 (facilitated glucose transporter), member 2
461	*SLC2A3*	solute carrier family 2 (facilitated glucose transporter), member 3
462	*SLC2A4*	solute carrier family 2 (facilitated glucose transporter), member 4
463	*SLC2A5*	solute carrier family 2 (facilitated glucose/fructose transporter), member 5
464	*SLC4A1*	solute carrier family 4, anion exchanger, member 1 (erythrocyte membrane protein band 3, Diego blood group)
465	*SLC4A2*	solute carrier family 4, anion exchanger, member 2 (erythrocyte membrane protein band 3-like 1)
466	*SLC4A4*	solute carrier family 4, sodium bicarbonate cotransporter, member 4
467	*SLC5A2*	solute carrier family 5 (sodium/glucose cotransporter), member 2
468	*SLC6A18*	solute carrier family 6, member 18
469	*SLC6A19*	solute carrier family 6 (neutral amino acid transporter), member 19
470	*SLC6A2*	solute carrier family 6 (neurotransmitter transporter, noradrenalin), member 2
471	*SLC6A4*	solute carrier family 6 (neurotransmitter transporter, serotonin), member 4
472	*SLC6A9*	solute carrier family 6 (neurotransmitter transporter, glycine), member 9
473	*SLC7A1*	solute carrier family 7 (cationic amino acid transporter, y+ system), member 1
474	*SLC8A1*	solute carrier family 8 (sodium/calcium exchanger), member 1
475	*SLC8A2*	solute carrier family 8 (sodium/calcium exchanger), member 2
476	*SLC9A1*	solute carrier family 9 (sodium/hydrogen exchanger), member 1
477	*SLC9A2*	solute carrier family 9 (sodium/hydrogen exchanger), member 2
478	*SLC9A5*	solute carrier family 9 (sodium/hydrogen exchanger), member 5
479	*SLCO1B1*	solute carrier organic anion transporter family, member 1B1
480	*SLCO4C1*	solute carrier organic anion transporter family, member 4C1
481	*SMAD1*	SMAD family member 1
482	*SMAD5*	SMAD family member 5
483	*SMO*	smoothened, frizzled family receptor
484	*SOD1*	superoxide dismutase 1, soluble
485	*SOD3*	superoxide dismutase 3, extracellular
486	*SORBS1*	sorbin and SH3 domain containing 1
487	*SORL1*	sortilin-related receptor, L(DLR class) A repeats containing
488	*SPARC*	secreted protein, acidic, cysteine-rich (osteonectin)
489	*SPP1*	secreted phosphoprotein 1
490	*SQRDL*	sulfide quinone reductase-like (yeast)
491	*SREBF1*	sterol regulatory element binding transcription factor 1
492	*SRY*	sex determining region Y
493	*STAT3*	signal transducer and activator of transcription 3 (acute-phase response factor)
494	*STEAP4*	STEAP family member 4
495	*SUCNR1*	succinate receptor 1
496	*TAP1*	transporter 1, ATP-binding cassette, sub-family B (MDR/TAP)
497	*TBX2*	T-box 2
498	*TBX4*	T-box 4
499	*TBXA2R*	thromboxane A2 receptor
500	*TBXAS1*	thromboxane A synthase 1 (platelet)
501	*TCAP*	titin-cap (telethonin)
502	*TGFA*	transforming growth factor, alpha
503	*TGFB1*	transforming growth factor, beta 1
504	*TGFB3*	transforming growth factor, beta 3
505	*TGFBR1*	transforming growth factor, beta receptor 1
506	*TGFBR2*	transforming growth factor, beta receptor II (70/80kDa)
507	*TGFBR3*	transforming growth factor, beta receptor III
508	*TH*	tyrosine hydroxylase
509	*THPO*	thrombopoietin
510	*THRA*	thyroid hormone receptor, alpha
511	*THUMPD1*	THUMP domain containing 1
512	*TIMM17A*	translocase of inner mitochondrial membrane 17 homolog A (yeast)
513	*TIMP1*	TIMP metallopeptidase inhibitor 1
514	*TIMP2*	TIMP metallopeptidase inhibitor 2
515	*TMOD2*	tropomodulin 2 (neuronal)
516	*TNC*	tenascin C
517	*TNF*	tumor necrosis factor
518	*TNFAIP3*	tumor necrosis factor, alpha-induced protein 3
519	*TNFRSF11B*	tumor necrosis factor receptor superfamily, member 11b
520	*TNFRSF1B*	tumor necrosis factor receptor superfamily, member 1B
521	*TNFSF12*	tumor necrosis factor (ligand) superfamily, member 12
522	*TNNI3*	troponin I type 3 (cardiac)
523	*TPH1*	tryptophan hydroxylase 1
524	*TPM1*	tropomyosin 1 (alpha)

525	*TRAK2*	trafficking protein, kinesin binding 2
526	*TRH*	thyrotropin-releasing hormone
527	*TRHR*	thyrotropin-releasing hormone receptor
528	*TRPC3*	transient receptor potential cation channel, subfamily C, member 3
529	*TRPC4*	transient receptor potential cation channel, subfamily C, member 4
530	*TRPC6*	transient receptor potential cation channel, subfamily C, member 6
531	*TRPM6*	transient receptor potential cation channel, subfamily M, member 6
532	*TRPM7*	transient receptor potential cation channel, subfamily M, member 7
533	*TRPV5*	transient receptor potential cation channel, subfamily V, member 5
534	*TSHR*	thyroid stimulating hormone receptor
535	*TTR*	transthyretin
536	*UBE2D2*	ubiquitin-conjugating enzyme E2D 2
537	*UCP1*	uncoupling protein 1 (mitochondrial, proton carrier)
538	*UCP2*	uncoupling protein 2 (mitochondrial, proton carrier)
539	*UCP3*	uncoupling protein 3 (mitochondrial, proton carrier)
540	*UGT1A6*	UDP glucuronosyltransferase 1 family, polypeptide A6
541	*UTS2*	urotensin 2
542	*UTS2R*	urotensin 2 receptor
543	*VCAM1*	vascular cell adhesion molecule 1
544	*VDR*	vitamin D (1,25- dihydroxyvitamin D3) receptor
545	*VEGFA*	vascular endothelial growth factor A
546	*VEGFB*	vascular endothelial growth factor B
547	*VEGFC*	vascular endothelial growth factor C
548	*VIP*	vasoactive intestinal peptide
549	*VNN1*	vanin 1
550	*VWF*	von Willebrand factor
551	*WISP1*	WNT1 inducible signaling pathway protein 1
552	*WNK1*	WNK lysine deficient protein kinase 1
553	*WNK3*	WNK lysine deficient protein kinase 3
554	*WNK4*	WNK lysine deficient protein kinase 4
555	*WRB*	tryptophan rich basic protein
556	*XDH*	xanthine dehydrogenase
557	*XPNPEP1*	X-prolyl aminopeptidase (aminopeptidase P) 1, soluble
558	*XRCC4*	X-ray repair complementing defective repair in Chinese hamster cells 4
559	*XYLT1*	xylosyltransferase I
560	*YY1*	YY1 transcription factor

Supplementary Table 5.

108 HTN genes identified by GWAS. Multiple genes located in the same GWAS locus are individually listed.

1	*ABCD4*	ATP-binding cassette, sub-family D (ALD), member 4
2	*ABHD16A*	abhydrolase domain containing 16A
3	*ADM*	adrenomedullin
4	*ADRB1*	adrenergic, beta-1-, receptor
5	*AGT*	angiotensinogen (serpin peptidase inhibitor, clade A, member 8)
6	*AGTRAP*	angiotensin II receptor-associated protein
7	*ALDH2*	aldehyde dehydrogenase 2 family (mitochondrial)
8	*APOM*	apolipoprotein M
9	*ARHGAP42*	Rho GTPase activating protein 42
10	*ARID3B*	AT rich interactive domain 3B (BRIGHT-like)
11	*ARID5B*	AT rich interactive domain 5B (MRF1-like)
12	*AS3MT*	arsenic (+3 oxidation state) methyltransferase
13	*ASIC5*	
14	*ATP2B1*	ATPase, Ca++ transporting, plasma membrane 1
15	*ATP5E*	ATP synthase, H+ transporting, mitochondrial F1 complex, epsilon subunit
16	*ATXN2*	ataxin 2
17	*BAG6*	BCL2-associated athanogene 6
18	*BLK*	B lymphoid tyrosine kinase
19	*C10orf107*	chromosome 10 open reading frame 107
20	*C10orf32*	chromosome 10 open reading frame 32
21	*C4orf22*	chromosome 4 open reading frame 22
22	*CACNA1H*	calcium channel, voltage-dependent, T type, alpha 1H subunit
23	*CACNB2*	calcium channel, voltage-dependent, beta 2 subunit
24	*CAPZA1*	capping protein (actin filament) muscle Z-line, alpha 1
25	*CASZ1*	castor zinc finger 1
26	*CDH13*	cadherin 13, H-cadherin (heart)
27	*CHRM3*	cholinergic receptor, muscarinic 3
28	*CLCN6*	chloride channel 6
29	*CNNM2*	cyclin M2
30	*CPLX3*	complexin 3
31	*CSK*	c-src tyrosine kinase
32	*CSNK2B*	casein kinase 2, beta polypeptide
33	*CTSO*	cathepsin O
34	*CTSZ*	cathepsin Z
35	*CYP11B2*	cytochrome P450, family 11, subfamily B, polypeptide 2
36	*CYP17A1*	cytochrome P450, family 17, subfamily A, polypeptide 1
37	*CYP1A1*	cytochrome P450, family 1, subfamily A, polypeptide 1
38	*CYP1A2*	cytochrome P450, family 1, subfamily A, polypeptide 2
39	*EBF1*	early B-cell factor 1
40	*EDN3*	endothelin 3
41	*ENPEP*	glutamyl aminopeptidase (aminopeptidase A)
42	*FAM167A*	family with sequence similarity 167, member A
43	*FAM19A3*	family with sequence similarity 19 (chemokine (C-C motif)-like), member A3
44	*FES*	feline sarcoma oncogene
45	*FGF5*	fibroblast growth factor 5
46	*FIGN*	fidgetin
47	*FURIN*	furin (paired basic amino acid cleaving enzyme)
48	*GATA4*	GATA binding protein 4
49	*GNAS*	GNAS complex locus
50	*GOSR2*	golgi SNAP receptor complex member 2
51	*GRB14*	growth factor receptor-bound protein 14
52	*GUCY1A3*	guanylate cyclase 1, soluble, alpha 3
53	*GUCY1B3*	guanylate cyclase 1, soluble, beta 3
54	*HEXIM1*	hexamethylene bis-acetamide inducible 1
55	*HEXIM2*	hexamethylene bis-acetamide inducible 2
56	*HFE*	hemochromatosis
57	*IPO7*	importin 7
58	*ITGA9*	integrin, alpha 9
59	*JAG1*	jagged 1
60	*LMAN1L*	lectin, mannose-binding, 1 like
61	*LY6G5B*	lymphocyte antigen 6 complex, locus G5B

62	*LY6G5C*	lymphocyte antigen 6 complex, locus G5C
63	*MAP4*	microtubule-associated protein 4
64	*MECOM*	MDS1 and EVI1 complex locus
65	*MOV10*	Mov10, Moloney leukemia virus 10, homolog (mouse)
66	*MTHFR*	methylenetetrahydrofolate reductase (NAD(P)H)
67	*NEIL2*	nei endonuclease VIII-like 2 (E. coli)
68	*NELFCD*	
69	*NOS3*	nitric oxide synthase 3 (endothelial cell)
70	*NPPA*	natriuretic peptide A
71	*NPPB*	natriuretic peptide B
72	*NPR3*	natriuretic peptide receptor C/guanylate cyclase C (atrionatriuretic peptide receptor C)
73	*NT5C2*	5'-nucleotidase, cytosolic II
74	*PGR*	progesterone receptor
75	*PHB*	prohibitin
76	*PIK3CG*	phosphoinositide-3-kinase, catalytic, gamma polypeptide
77	*PLCD3*	phospholipase C, delta 3
78	*PLCE1*	phospholipase C, epsilon 1
79	*PLEKHA7*	pleckstrin homology domain containing, family A member 7
80	*PMS1*	PMS1 postmeiotic segregation increased 1 (S. cerevisiae)
81	*PRDM8*	PR domain containing 8
82	*PRRC2A*	proline-rich coiled-coil 2A
83	*RHOBTB1*	Rho-related BTB domain containing 1
84	*RHOC*	ras homolog gene family, member C
85	*RTKN2*	rhotekin 2
86	*SH2B3*	SH2B adaptor protein 3
87	*SLC22A4*	solute carrier family 22 (organic cation/ergothioneine transporter), member 4
88	*SLC39A8*	solute carrier family 39 (zinc transporter), member 8
89	*SLC4A7*	solute carrier family 4, sodium bicarbonate cotransporter, member 7
90	*SLMO2*	slowmo homolog 2 (Drosophila)
91	*SNORA38*	small nucleolar RNA, H/ACA box 38
92	*ST7L*	suppression of tumorigenicity 7 like
93	*STK39*	serine threonine kinase 39
94	*TBX3*	T-box 3
95	*TBX5*	T-box 5
96	*TDO2*	tryptophan 2,3-dioxygenase
97	*TMEM133*	transmembrane protein 133
98	*TMEM26*	transmembrane protein 26
99	*TUBB1*	tubulin, beta 1 class VI
100	*ULK3*	unc-51-like kinase 3 (C. elegans)
101	*ULK4*	unc-51-like kinase 4 (C. elegans)
102	*UMOD*	uromodulin
103	*WNT2B*	wingless-type MMTV integration site family, member 2B
104	*YWHAZ*	tyrosine 3-monooxygenase/tryptophan 5-monooxygenase activation protein, zeta polypeptide
105	*ZFAT*	zinc finger and AT hook domain containing
106	*ZNF652*	zinc finger protein 652
107	*ZNF831*	zinc finger protein 831
108	*ZP4*	zona pellucida glycoprotein 4
